# Food groups and risk of type 2 diabetes mellitus: a systematic review and meta-analysis of prospective studies

**DOI:** 10.1007/s10654-017-0246-y

**Published:** 2017-04-10

**Authors:** Lukas Schwingshackl, Georg Hoffmann, Anna-Maria Lampousi, Sven Knüppel, Khalid Iqbal, Carolina Schwedhelm, Angela Bechthold, Sabrina Schlesinger, Heiner Boeing

**Affiliations:** 1 German Institute of Human Nutrition Potsdam-Rehbruecke (DIfE), Arthur-Scheunert-Allee 114-116, 14558 Nuthetal, Germany; 2 Department of Nutritional Sciences, University of Vienna, Althanstraße 14, UZA II, 1090 Vienna, Austria; 3 German Nutrition Society, Godesberger Allee 18, 53175 Bonn, Germany; 4 Department of Epidemiology and Biostatistics, School of Public Health Imperial College London, St. Mary’s Campus, Norfolk Place, Paddington, London, W2 1PG UK; 5 Institute for Biometry and Epidemiology, Leibniz Institute for Diabetes Research, Heinrich Heine University Düsseldorf, 40225 Duesseldorf, Germany

**Keywords:** Food, Diet, Meta-analysis, Dose–response, Type 2 diabetes

## Abstract

The aim of this systematic review and meta-analysis was to synthesize the knowledge about the relation between intake of 12 major food groups and risk of type 2 diabetes (T2D). We conducted a systematic search in PubMed, Embase, Medline (Ovid), Cochrane Central, and Google Scholar for prospective studies investigating the association between whole grains, refined grains, vegetables, fruits, nuts, legumes, eggs, dairy, fish, red meat, processed meat, and sugar-sweetened beverages (SSB) on risk of T2D. Summary relative risks were estimated using a random effects model by contrasting categories, and for linear and non-linear dose–response relationships. Six out of the 12 food-groups showed a significant relation with risk of T2D, three of them a decrease of risk with increasing consumption (whole grains, fruits, and dairy), and three an increase of risk with increasing consumption (red meat, processed meat, and SSB) in the linear dose–response meta-analysis. There was evidence of a non-linear relationship between fruits, vegetables, processed meat, whole grains, and SSB and T2D risk. Optimal consumption of risk-decreasing foods resulted in a 42% reduction, and consumption of risk-increasing foods was associated with a threefold T2D risk, compared to non-consumption. The meta-evidence was graded “low” for legumes and nuts; “moderate” for refined grains, vegetables, fruit, eggs, dairy, and fish; and “high” for processed meat, red meat, whole grains, and SSB. Among the investigated food groups, selecting specific optimal intakes can lead to a considerable change in risk of T2D.

**Electronic supplementary material:**

The online version of this article (doi:10.1007/s10654-017-0246-y) contains supplementary material, which is available to authorized users.

## Background

The global prevalence of type 2 diabetes (T2D) is increasing rapidly, running parallel to the increase in obesity, the reduction in physical activity/adoption of a sedentary lifestyle, and changes in diet towards unhealthy eating behaviors. It has been estimated that 415 million persons had T2D in 2015, and the number has been projected to increase to 642 million by 2040 [1].

Therefore, implementation of effective T2D prevention strategies, as well as early detection programs is of major importance to reduce the health burden of the disease [2]. To prevent onset of T2D at an early age and to lower life-long risk of getting T2D, optimal selection of food and dietary factors have been recognized to play a critical role. Previous meta-analyses of prospective studies showed that whole grains were associated with lower T2D risk, whereas red meat, processed meat, and sugar sweetened beverages (SSB) were associated with increased risk [3–5].

Moreover, healthy eating patterns assessed by hypothesis-driven approaches such as the Mediterranean diet score, the Healthy Eating Index, the Alternate Healthy Eating Index and the DASH dietary score have been associated with reduced risk of T2D [6, 7]. The complex relation between diet and health, approached via these dietary patterns, is a reminder of the fact that humans do not consume nutrients but rather a mixture of individual foods [8, 9]. Concentrating on food groups, thus, may help to understand the role dietary factors play on the risk of developing T2D on a level which could be more easily communicated to the public and could form the basis for dietary recommendations for preventing chronic diseases.

In this context, the following 12 food groups might be of interest when analyzing diet and risk of T2D because they are the basis for most diet quality indices/scores [6, 7, 10], as previously reported [11]: whole grains/cereals, refined grains/cereals, vegetables, fruits, nuts, legumes, eggs, dairy products (milk, cheese, yogurt), fish, red meat, processed meat, and sugar-sweetened beverages.

Furthermore, the quality of evidence provided by meta-analyses of cohort studies is rarely assessed. Consequently, one of the most important questions that remain to be answered is which food groups show high quality meta-evidence of protective or detrimental effects in relation to risk of T2D using an integrative approach.

Thus, we synthesized all available data from prospective studies for investigating the associations of the 12 a priori defined food groups, including whole grains, refined grains, vegetables, fruits, nuts, legumes, eggs, dairy, fish, red meat, processed meat, and SSB with risk of T2D. We specifically aimed to clarify the strength and shape of the dose–response relationship and to find optimal food intakes for a low disease risk.

## Methods

The review was registered in PROSPERO (www.crd.york.ac.uk/prospero/index.asp, identifier CRD42016037069). This systematic review was planned and conducted according to the standards of the Meta-analysis of Observational Studies in Epidemiology [12].

## Search strategy

Queries of literature were performed using the electronic databases PubMed, Embase, Medline (Ovid), Cochrane Central, and Google Scholar until February 2017 with no restriction to calendar date and language using the following search terms (Supplementary Appendix S1).

Moreover, the reference lists from the retrieved articles, systematic reviews, and meta-analyses were checked to search for further relevant studies. The literature search was conducted by two authors (LS, AML), with disagreement resolved by consensus of another reviewer.

## Study selection

Studies were included in the meta-analysis if they met all of the following criteria: (1) prospective design studies (cohort studies, nested case–control studies, case-cohort studies, follow-up of RCTs) that were peer-reviewed and available in full-text; (2) information about the association for ≥1 of the following twelve food groups: whole grains/cereals, refined grains/cereals, vegetables, fruits, nuts, legumes, eggs, dairy products, fish, red meat, processed meat, and SSB on risk of T2D; (3) Participants ≥18 years; and (4) considering T2D as outcome (study population had to be free of T2D at the onset of the study).

## Data extraction

After determination of the study selection, two reviewers extracted the following characteristics: the first author’s last name, year of publication, study origin, cohort name, sample size, number of cases, age at entry, sex, study length, outcome, outcome assessment, assessment of food group, quantity of food, risk estimate (most adjusted measures) (hazard ratios (HR), risk ratios (RR) with their corresponding 95% confidence intervals (CIs)), and adjustment.

When a study provided several risk estimates, the multivariable adjusted model was chosen. When only separate risk estimates for male and female participants were available in a study, we combined the RRs using a fixed effects model before inclusion in the meta-analysis.

## Risk of bias

In a previous analysis of 50 randomly selected meta-analyses of cohort studies exploring the field of nutritional sciences we could show that 20 meta-analyses (40%) applied no quality assessment score, and 19 (38%) used the Newcastle Ottawa Scale [13]. However, Stang [14] commented that the Newcastle–Ottawa Scale includes quality items that are not valid (e.g., the “representativeness of the exposed cohort” item), and concluded that this score appeared to be unacceptable for the quality ranking of case–control and cohort studies in meta-analyses. Therefore we developed a risk of-bias checklist (that also take into account nutrition research–specific requirements) with 4 sub-items, awarding a maximum of 2 points (maximum of 0.5 points for each sub-item) [13]:

1. ascertainment of exposure (low risk of bias: validated, calibrated FFQ or 24-h recall, diet history, or diet records (multiple days));
2. assessment of outcome (low risk of bias: record linkage (ICD codes), accepted clinical criteria, self-reported and validated);
3. adequacy of follow-up length (low risk of bias: >5 years);
4. and adjusted basic model (low risk of bias, ≥2 factors: e.g. sex, education, ethnicity; if only one sex included, then ≥1 factor) and outcome-relevant adjustments (low risk of bias, ≥3 factors: e.g. BMI, smoking, energy intake, family history of diabetes, physical activity).

Studies were classified as being at low risk of bias (2 points) only if none of the domains established a high/unclear risk of bias, high risk of bias (if at least one sub-item was rated as high risk), and moderate/unclear risk (if at least one sub-item was rated as moderate/unclear risk).

## Statistical analysis

A random effects model was used to calculate summary RRs and 95% CIs for the associations between T2D and the highest versus the lowest intake category for each of the 12 pre-defined food groups and for the dose–response analysis [15], which incorporated both within- and between-study variability. To evaluate the weighting of each study, the standard error for the logarithm RR/HR of each study was calculated and regarded as the estimated variance of the logarithm HR/RR, using an inverse variance method [15].

The method described by Greenland and Longnecker [16, 17] was applied for the dose–response analysis and computed study-specific slopes (linear trends) and 95% CIs from the natural logs of the RRs and CIs across intake categories of the 12 pre-defined food groups. The method requires that the distribution of cases and person-years or non-cases and the RRs with the 95% CI for at least three quantitative exposure categories are known.

When studies reported only the total number of cases or total person-years and the exposure was defined in quantiles, the distribution of cases or person-years was calculated dividing the total number by the number of quantiles. Whenever reported, the mean or median intake by category was assigned to the corresponding RR. The midpoint was calculated for studies that only reported a range of intake by category. When the intake range was open-ended, we assumed that its width was the same as the adjacent category.

The dose–response was expressed in the following servings: whole grains/cereals (30 g/day), refined grains/cereals (30 g/day), vegetables (100 g/day), fruits (100 g/day), nuts (28 g/day), legumes (50 g/day), eggs (50 g/day), dairy products (200 g/day), fish (100 g/day), red meat (100 g/day), processed meat (50 g/day), and SSB (250 ml/day). For studies that reported intake only as serving size, we used recommended conversions (Supplementary Table S1).

To examine possible nonlinear associations, we calculated restricted cubic splines for each study with more than three categories of exposure, using three fixed knots at 10, 50, and 90% through the total distribution of the reported intake, and combined them using multivariable meta-analysis [18].

Moreover, the T2D risk reduction potential of foods was calculated by multiplying the RR by selecting an optimal consumption (serving category with the strongest association) of risk-decreasing foods, and risk-increasing foods, respectively.

To explore heterogeneity between studies, we used the Q test and the I^2^ statistic (with a value of I^2^ >50% considered to represent potentially important heterogeneity [19]). In addition, to identify potential sources of heterogeneity, we stratified the dose–response meta-analysis by subgroups: sex, age (mean or median ≥50 vs. <50 years), length of follow-up (mean or median ≥10 vs. <10 years), geographic location (Europe, America, Asia and Australia), number of cases (≥1000 vs. <1000), outcome assessment (self-reported vs. diagnosed by physician vs. registry), and dietary assessment methods (FFQ vs. 24 h recall/diet history). For dairy products we stratified the analysis comparing low- versus high-fat dairy products. Furthermore, we performed sensitivity analysis for studies with low risk of bias.

Potential small-study effects, such as publication bias, were explored using Egger´s test and funnel plots [20] when at least 10 studies were available, as recommended by the Cochrane Handbook [21]. Review Manager 5.3 (Nordic Cochrane Center, Copenhagen), and Stata version 14 software (StataCorp, College Station, TX) were used for the statistical analyses.

## Quality of meta-evidence

To evaluate the meta-evidence for the association between 12 pre-defined food groups and T2D (quality of evidence of meta-analyses was defined as the confidence in the estimate) we applied the NutriGrade scoring system (max 10 points), which comprises the following items: (1) risk of bias/study quality/study limitations, (2) precision, (3) heterogeneity, (4) directness, (5) publication bias, (6) funding bias, (7) study design (only for meta-analyses of randomized controlled trials), (8) effect size [13]. Based on this scoring system we recommend four categories to judge the meta-evidence: high, moderate, and low, and very low taking into account the following cut-points: ≥8 points (high meta-evidence); 6–7.99 points (moderate meta-evidence); 4–5.99 (low meta-evidence); and 0–3.99 (very low meta-evidence) [13].

## Results

**Fig. 1:**
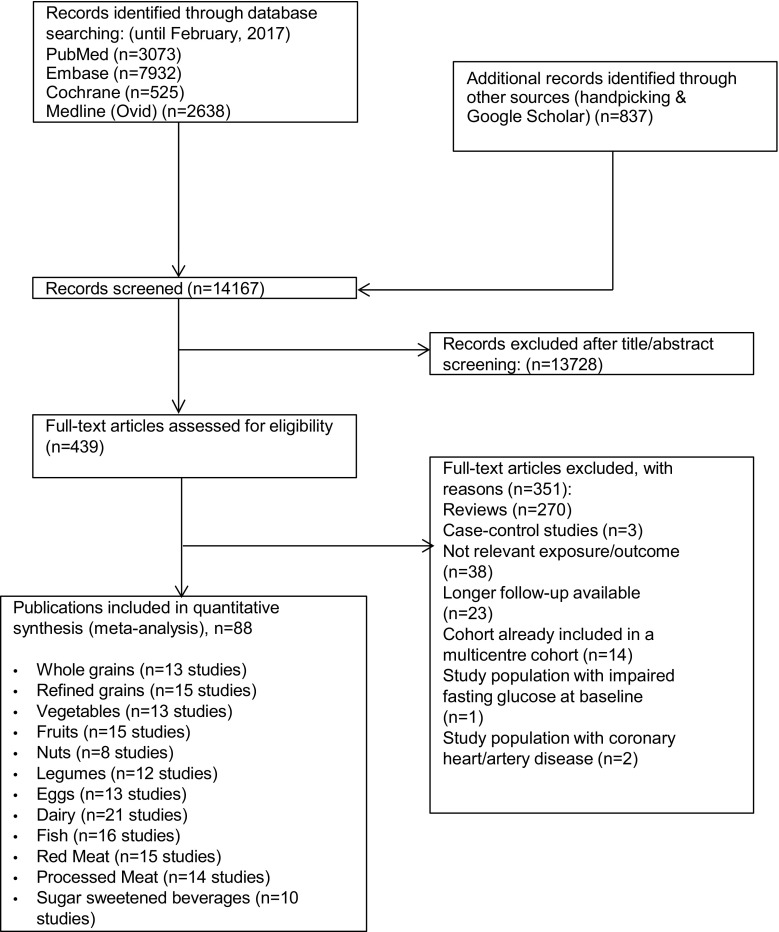
Flow chart of study selection

Out of 14,167 records identified by the literature search, 439 full text articles were assessed in detail as they reported on one or more of the twelve foods groups and T2D in the title/abstract (Fig. 1).

Thirteen prospective studies were included in the meta-analysis for consumption of whole grains (Supplementary Table S2, References S1–11), 15 for refined grains (Supplementary Table S3, References S1–8, 12–15), 13 for vegetables (Supplementary Table S4, References S1, 8, 16–25), 15 for fruits (Supplementary Table S5, References S1, 8, 16–21, 23–26), 8 for nuts (Supplementary Table S6, References S9, 27–32), 12 for legumes (Supplementary Table S7, References S1–2, 8–9, 22, 30, 33–36), 13 for eggs (Supplementary Table S8, References S9, 23, 37–46), 21 for dairy products (Supplementary Table S9, References S11, 23, 37, 41, 47–61), 16 for fish (Supplementary Table S10, References S17, 23, 37, 41, 62–70), 15 for red meat (Supplementary Table S11, References S23, 41, 71–79), 14 for processed meat (Supplementary Table S12, References S23, 41, 71–78), and 10 for consumption of SSB (Supplementary Table S13, References S80–88).

### Whole grains

**Fig. 2:**
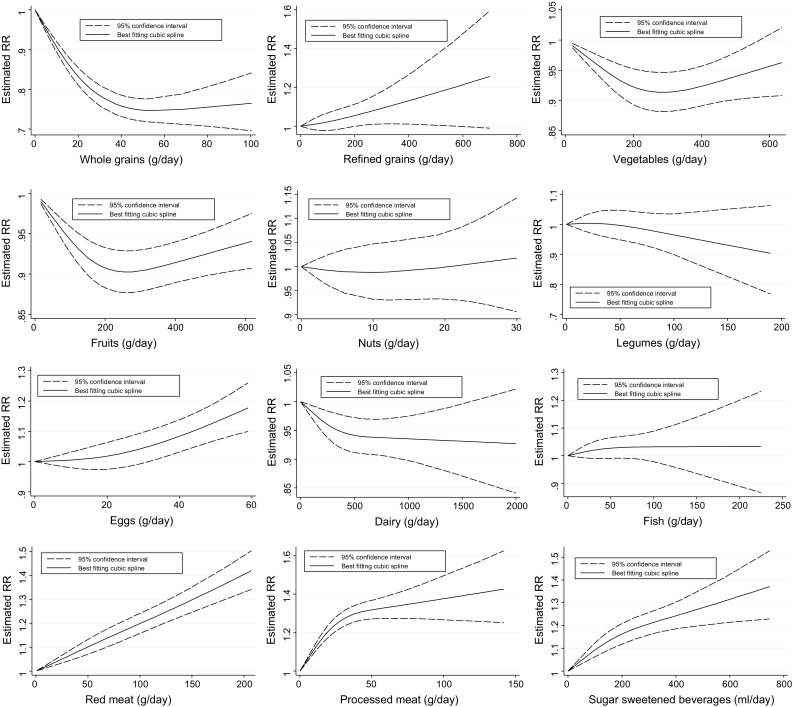
Non-linear dose–response relation between daily intakes whole grains (*p*_non-linearity_ < 0.001), refined grains (*p*_non-linearity_ = 0.07), vegetables (*p*_non-linearity_ < 0.001), fruits (*p*_non-linearity_ < 0.001), nuts (*p*_non-linearity_ = 0.67), legumes (*p*_non-linearity_ = 0.44), eggs (*p*_non-linearity_ = 0.09), dairy (*p*_non-linearity_ = 0.89), fish (*p*_non-linearity_ = 0.48), red meat (*p*_non-linearity_ = 0.30), processed meat (*p*_non-linearity_ < 0.001), and sugar sweetened beverages (*p*_non-linearity_ = 0.007) and risk of T2D

Thirteen studies with 29,633 T2D cases were included in the high vs. low intake meta-analysis (overall intake range: 0–302 g/day). Comparing extreme categories, a strong inverse association between T2D and whole grain intake was observed (RR: 0.77; 95% CI 0.71–0.84, I^2^ = 86%) (Supplementary Figure S1). Each additional daily 30 g of whole grains was inversely associated with T2D risk (RR: 0.87; 95% CI 0.82–0.93, I^2^ = 91%, n = 12 studies) (Supplementary Figure S2). The inverse associations and heterogeneity persisted in additional analyses stratified by sex, age, follow-up length, geographic location, number of cases, dietary assessment, and outcome assessment (Supplementary Table S14). Evidence of heterogeneity between subgroups in stratified analyses was observed for geographic location, dietary assessment method, and outcome assessment. There was significant evidence for small study effects in the high versus low meta-analysis, but not in the dose–response meta-analysis. Visual inspection of the funnel plot suggests that small studies showing positive association may be missing (Supplementary Figure S25). There was evidence of a non-linear dose–response association; the risk of T2D decreased by 25% with increasing intake of whole grains up to ~50 g/day. Small benefits for increasing intake above this value were observed (Fig. 2).

### Refined grains

Fifteen studies with 24,517 T2D cases were included in the high versus low intake meta-analysis (overall intake range: 0–700 g/day). No association was observed for the highest versus lowest refined grain intake category (RR: 1.01; 95% CI 0.92–1.10, I^2^ = 54%) (Supplementary Figure S3), and for each additional daily 30 g (RR: 1.01; 95% CI 0.99–1.03, I^2^ = 59%, n = 14 studies) (Supplementary Figure S4). No significant association or heterogeneity were observed within and between subgroups of the stratified analyses, except for a positive association among participants <50 years of age (Supplementary Table S15). There was no evidence for small study effects in the high vs. low and in the dose–response meta-analysis (Supplementary Figure S26). There was no evidence of a non-linear dose–response association. However, 200–400 g/day of refined grains were associated with a 6–14% increased risk of T2D (Fig. 2).

### Vegetables

Thirteen studies with 63,299 T2D cases were included in the high versus low intake meta-analysis (overall intake range: 20.5–636 g/day). A borderline inverse association was observed for the high versus low (RR: 0.95; 95% CI 0.89–1.01, I^2^ = 59%) (Supplementary Figure S5) and dose–response analysis (RR: 0.98; 95% CI 0.96–1.00, I^2^ = 62%, n = 11 studies) (Supplementary Figure S6). The inverse association was observed only in Asian and Australian studies, but not for American and European studies, and in studies with a lower number of cases (Supplementary Table S16). There was no evidence of heterogeneity between subgroups in stratified analyses. No evidence for small study effects was observed, and visual inspection of the funnel plot suggests symmetry (Supplementary Figure S27). There was evidence of a non-linear dose–response association; the risk of T2D decreased by 9% with increasing intake up to 300 g/day. No benefit for increasing intake is apparent above this value (Fig. 2).

### Fruits

Fifteen studies with 70,968 T2D cases were included in the high versus low intake meta-analyses (overall intake range: 10–618 g/day). A borderline inverse association was observed (RR: 0.96; 95% CI 0.93–1.00, I^2^ = 29%) (Supplementary Figure S7). Each additional daily 100 g of fruits was inversely associated with T2D risk (RR: 0.98; 95% CI 0.97–1.00, I^2^ = 21%, n = 13 studies) (Supplementary Figure S8). The inverse association was observed only in studies with a longer-term follow-up (≥10 years), and including participants younger than 50 years of age (Supplementary Table S17). There was no evidence of heterogeneity between subgroups in stratified analyses. No evidence for small study effects was observed, and visual inspection of the funnel plot suggests symmetry (Supplementary Figure S28). There was evidence of a non-linear dose–response association; the risk of T2D decreased by 10% with increasing intakes of fruits up to 200–300 g/day. No benefit for increasing intake is apparent above this value (Fig. 2).

### Nuts

Eight studies with 27,016 T2D cases were included in the high versus low intake meta-analysis (overall intake range: 0–27 g/day). No significant association was observed for the highest versus lowest nut intake category (RR: 0.95; 95% CI 0.85–1.05, I^2^ = 67%) (Supplementary Figure S9), and for each additional daily 28 g (RR: 0.89; 95% CI 0.71–1.12, I^2^ = 77%, n = 7 studies) (Supplementary Figure S10). We observed a significant inverse association for studies conducted in Asian countries and for studies with a shorter-term follow-up, confirmed by significant heterogeneity between subgroups (Supplementary Table S18). There was no evidence of a non-linear dose–response association (Fig. 2).

### Legumes

Twelve studies with 26,778 T2D cases were included in the high versus low intake meta-analyses (overall intake range: 0–190 g/day). No significant association was observed for the highest versus lowest legume intake category (RR: 0.96; 95% CI 0.87–1.05, I^2^ = 85%) (Supplementary Figure S11), and for each additional daily 50 g (RR: 1.00; 95% CI 0.92–1.09, I^2^ = 87%, n = 12 studies) (Supplementary Figure S12). There was no evidence of heterogeneity between subgroups in stratified analyses, except for an inverse association among participants <50 years of age (Supplementary Table S19). No evidence for small study effects was observed, but visual inspection of the funnel plot suggests asymmetry (Supplementary Figure S29). There was no evidence of a non-linear dose–response association (Fig. 2).

### Eggs

Thirteen studies with 17,629 T2D cases were included in the highest compared with the lowest intake category analysis (overall intake range: 0–60 g/day). No significant association was observed for the highest versus lowest egg intake category (RR: 1.08; 95% CI 0.95–1.22, I^2^ = 69%) (Supplementary Figure S13), and for each additional daily 30 g (RR: 1.08; 95% CI 0.95–1.22, I^2^ = 77%, n = 13 studies) (Supplementary Figure S14). We observed a strong positive association for studies conducted in America in the dose–response analysis, but not for Asian and European studies (Supplementary Table S20). Moreover, significant positive associations were observed for studies with ≥1000 diabetes cases, using FFQ, and self-reported T2D diagnosis. There was some evidence of heterogeneity between subgroups in stratified analyses (geographic location, and number of cases). No evidence for small study effects was observed, and visual inspection of the funnel plot suggests symmetry (Supplementary Figure S30). There was little evidence of a non-linear dose–response association (*p* = 0.09) (Fig. 2); the risk of T2D increased by 13% with increasing intake of eggs up to 50 g/day.

### Dairy

Twenty-one studies with 44,474 T2D cases were included in the highest compared with the lowest intake category meta-analysis (overall intake range: 0–2000 g/day). A significant inverse association was observed (RR: 0.91; 95% CI 0.85–0.97, I^2^ = 63%) (Supplementary Figure S15). Each additional daily 200 g of dairy products was inversely associated with diabetes risk (RR: 0.97; 95% CI 0.94–0.99, I^2^ = 74%, n = 21 studies) (Supplementary Figure S16). The inverse association was observed only in Asian and Australian studies, but not for American and European studies. Moreover, significant associations were observed for studies with <1000 T2D cases, participants ≥50 years of age, and a shorter follow-up (<10 years). In subgroup analyses low-fat dairy products showed a borderline inverse association, whereas no association could be observed for high-fat dairy products (Supplementary Table S21). Some evidence of heterogeneity between subgroups in stratified analyses was observed (age, length of follow-up, number of cases, and dietary assessment). There was significant evidence for small study effects in the dose–response meta-analysis, but not in the high versus low meta-analysis (Supplementary Figure S31). Visual inspection of the funnel plot suggests that small studies showing positive or null association may be missing. There was no evidence of a non-linear dose–response association between dairy products and T2D; the risk decreased by 6% with increasing intake up to 400–600 g/day. No benefit for increasing intake was apparent above this value (Fig. 2).

### Fish

Sixteen studies with 45,029 T2D cases were included in the highest compared with the lowest intake category meta-analysis (overall intake range: 0–225 g/day). No significant association was observed for the highest versus lowest fish intake category (RR: 1.04; 95% CI 0.95–1.13, I^2^ = 76%) (Supplementary Figure S17), and for each additional daily 100 g (RR: 1.09; 95% CI 0.93–1.28, I^2^ = 84%, n = 15 studies) (Supplementary Figure S18). We observed a strong positive association for studies conducted in America between fish intake and risk of T2D, with stronger associations in the dose–response analysis (Supplementary Table S22), and an inverse association in Asian studies. We found statistically significant heterogeneity between subgroups of geographic location and length of follow-up. No evidence for small study effects was observed, and visual inspection of the funnel plot suggests symmetry (Supplementary Figure S32). There was no evidence of a non-linear dose–response association (Fig. 2).

### Red meat

Fifteen studies with 45,702 T2D cases were included in the high versus low intake meta-analysis (overall intake range: 0–207 g/day). A significant positive association was observed (RR: 1.21; 95% CI 1.13–1.30, I^2^ = 65%) (Supplementary Figure S19). Each additional daily 100 g of red meat was positively associated with T2D risk (RR: 1.17; 95% CI 1.08–1.26, I^2^ = 83%, n = 14 studies) (Supplementary Figure S20). The observed positive associations and heterogeneity persisted in additional analyses stratified by age, sex, follow-up length, geographic location, number of cases, and dietary assessment method. We observed a positive association for studies conducted in America and Europe in both the high versus low and the dose–response analysis, but not in Asian studies (Supplementary Table S23). There was some evidence of heterogeneity between subgroups in stratified analyses (≥1000 vs. <1000 cases). There was no significant evidence for small study effects in the both high versus low and dose–response meta-analysis. Visual inspection of the funnel plot suggests symmetry (Supplementary Figure S33). There was no evidence of a non-linear dose–response association (Fig. 2).

### Processed meat

Fourteen studies with 43,781 T2D cases were included in the high versus low intake meta-analysis (overall intake range: 0–142 g/day). A significant positive association was observed (RR: 1.27; 95% CI 1.20–1.35, I^2^ = 55%) (Supplementary Figure S21). Each additional daily 50 g of processed meat was strongly associated with diabetes risk (RR: 1.37; 95% CI 1.22–1.55, I^2^ = 88%, n = 14 studies) (Supplementary Figure S22). The observed positive associations and heterogeneity persisted in additional stratified analyses (Supplementary Table S24). We detected evidence of heterogeneity between subgroups in stratified analyses for geographic location, dietary assessment, and outcome assessment. There was significant evidence for small study effects in the dose–response meta-analysis, but not in the high versus low meta-analysis. Visual inspection of the funnel plot suggests that small studies showing inverse or null association may be missing (Supplementary Figure S34). There was evidence of a non-linear dose–response association; the risk of T2D increased by 30% with increasing intakes up to 50 g/day. Moderate additional detrimental effects for increasing intake above this value were observed (Fig. 2).

### Sugar sweetened beverages (SSB)

Ten studies with 25,600 T2D cases were included in the high versus low intake meta-analysis (overall intake range: 0–748 ml/day). A significant positive association between T2D and SSB was observed (RR: 1.30; 95% CI 1.20–1.40, I^2^ = 34%) (Supplementary Figure S23). Each additional daily 250 ml of SSB was associated with T2D risk (RR: 1.21; 95% CI 1.12–1.31, I^2^ = 78%, n = 10 studies) (Supplementary Figure S24). The observed positive associations persisted in additional stratified analyses (Supplementary Table S25). Some evidence of heterogeneity between subgroups in stratified analyses (follow-up length) was observed. There was significant evidence for small study effects in the dose–response meta-analysis, but not in the high versus low meta-analysis (Supplementary Figure S35). Visual inspection of the funnel plot suggests that small studies showing inverse or null association may be missing. There was evidence of a non-linear dose–response association but the curve shows an increase of risk of T2D throughout all the range of SSB investigated (Fig. 2).

## Summary across food groups

Table 1 shows the risk ratio for T2D from non-linear dose–response analysis of the 12-prefinded food groups according to servings/day. Optimal consumption of risk-decreasing foods (2 servings/day of whole grains; 2–3 servings/day of vegetables; 2–3 servings/day of fruits; 3 servings/day of dairy) results in a 42% reduction compared to non-consumption of these foods. The highest reduction in risk for T2D in terms of servings could be observed for whole grains; 50 g/day was associated with a 25% reduction in risk compared to non-consumption of this food group. Furthermore, the table clearly shows that increasing the daily consumption of foods with inverse relation to risk of T2D beyond 2 servings of whole grains (60 g/day), 2–3 servings of vegetables and fruits (160–240 g/day respectively), and 3 servings of dairy (400–600 g/day) will not further reduce the risk. We could further calculate that a consumption of risk-increasing foods of 2 servings/day of red meat (170 g/day), 4 servings/day of processed meat (105 g/day), 3 servings/day of SSB (750 ml/day), and 1 serving/day of eggs (55 g/day) is associated with a threefold increased risk of T2D, compared to non-consumption. Not consuming these foods would reduce the risk of T2D by about 70%. The highest reduction of risk per serving can be obtained by reducing red and processed meat, and SSB. The T2D risk reduction potential of foods was cumulatively calculated by selecting an optimal consumption of whole grains, vegetables, fruits, and dairy, and non-consumption of red meat, processed meat, SSB, and eggs. According to these calculations, a reduction of risk of T2D of about ~80% could be achieved.

**Table 1:** Relative risks from nonlinear dose–response analysis of 12 pre-defined food groups and risk of type 2 diabetes according to servings/day

Food group	Risk ratio (RR), 95% CI							
*Inverse association*								
Servings per day	0	1	2	3	4	5	6	7
Whole grains (1 serving = 30 g/day)	1.00	0.78 (0.76–0.81)	0.75 (0.71–0.79)	0.76 (0.70–0.82)	NA	NA	NA	NA
Vegetables (1 serving = 80 g/day)	1.00	0.96 (0.94–0.98)	0.93 (0.90–0.96)	0.92 (0.89–0.95)	0.92 (0.88–0.95)	0.92 (0.89–0.96)	0.94 (0.90–0.97)	0.95 (0.90–0.99)
Fruits (1 serving = 80 g/day)	1.00	0.95 (0.94–0.96)	0.92 (0.89–0.94)	0.90 (0.88–0.93)	0.90 (0.88–0.93)	0.91 (0.89–0.94)	0.92 (0.90–0.95)	0.93 (0.90–0.96)
Dairy (1 serving = 200 g/day)	1.00	0.97 (0.95–0.99)	0.95 (0.92–0.98)	0.94 (0.91–0.97)	0.94 (0.91–0.97)	0.94 (0.90–0.97)	NA	NA
*Positive association*								
Refined grains (1 serving = 30 g/day)	1.00	1.01 (0.99–1.02)	1.01 (0.98–1.05)	1.02 (0.98–1.06)	1.03 (0.98–1.08)	1.04 (0.99–1.09)	1.05 (0.99–1.10)	1.06 (1.00–1.12)
Eggs (1 serving = 55 g/day)	1.00	1.16 (1.09–1.23)	NA	NA	NA	NA	NA	NA
Red meat (1 serving = 85 g/day)	1.00	1.18 (1.14–1.21)	1.37 (1.30–1.43)	NA	NA	NA	NA	NA
Processed meat (1 serving = 35 g/day)	1.00	1.29 (1.25–1.33)	1.35 (1.28–1.42)	1.39 (1.27–1.54)	1.43 (1.26–1.63)	NA	NA	NA
Sugar sweetened beverages (1 serving = 250 ml/day)	1.00	1.19 (1.14–1.23)	1.28 (1.20–1.38)	1.37 (1.23–1.53)	NA	NA	NA	NA
*No association*								
Nuts (1 serving = 28 g/day)	1.00	1.01 (0.92–1.12)	NA	NA	NA	NA	NA	NA
Legumes (1 serving = 100 g/day)	1.00	1.00 (0.95–1.05)	0.97 (0.90–1.03)	NA	NA	NA	NA	NA
Fish (1 serving = 100 g/day)	1.00	1.03 (0.98–1.09)	1.03 (0.89–1.20)	NA	NA	NA	NA	NA

## Risk of bias

The results varied little by methodological assumption, including only studies with a low risk of bias (Supplementary Table S14–25). Findings including studies with low risk of bias suggest stronger inverse associations for fruit and whole grain consumption, and stronger positive associations for red meat and SSB intake in the dose–response analyses.

## Quality of meta-evidence

We rated the quality of meta-evidence for the 12 food groups [13]. The NutriGrade meta-evidence grading was rated “low” for legumes and nuts; “moderate” for refined grains, vegetables, fruit, eggs, dairy and fish; and “high” for processed meat, red meat, whole grains, and SSB. (Supplementary Table S26).

## Discussion

In this meta-analysis we systematically assessed whether associations exist between 12 a priori defined food groups and T2D risk by applying high versus low, linear and non-linear dose–response analyses. In high versus low and linear dose–response meta-analysis, a link with T2D risk was identified for 6 out of the 12 food groups; for the consumption of whole grains, dairy, and fruits this association was inverse; for processed meat, red meat, and SSB consumption, the association was positive. However, we observed that 5 from the 12 food groups showed also a significant nonlinear association. A high confidence in the effect estimate according to the NutriGrade scoring was seen for whole grains, for red and processed meat, and for SSB. This observation is concordant with the overall importance of these food groups for T2D occurrence and prevention.

Our findings are in line with previous meta-analyses conducted mostly on single food groups and not synoptically for 12 food groups as it was done in the present systematic review.

Taken together, an inverse association between dairy products, fruits, whole grains and T2D as well as a positive association between red and processed meat, SSB and risk of T2D was reported [22–26], while no significant linear association for the intake of eggs, fish, nuts, vegetables, and refined grains could be found [22, 27–30]. However, most of these studies did not investigate non-linear relationships. By this statistical approach we detected that one serving/day of eggs was associated with increased risk of T2D in a non-linear model.

A protective effect of whole grain consumption against T2D is biologically plausible and several mechanisms may operate to reduce the risk. Thus, reduced adiposity may partly explain the beneficial effects of whole grains in the prevention of T2D [22]. In addition, in observational studies, an intake of whole grains was associated with lower fasting glucose and insulin concentrations [31, 32]. Several nutrients and phytochemicals such as soluble fiber, resistant starch, phytic acid, magnesium, zinc, selenium, and potassium could mediate the effect of whole grains [33].

There is strong evidence that SSB consumption is associated with weight gain and obesity in adults [34]. Sugars in SSB acutely increase blood glucose levels and have a high glycemic index, which represents a risk factor for T2D [35]. Fructose in SSB promotes hepatic lipogenesis and furthers insulin resistance [36]. There might also be a specific role of this type of beverage for impairment of the otherwise working regulation of hunger and satiety [37].

There is consistent evidence that red and processed meat is associated with T2D risk. The positive association for red meat could not be confirmed pooling two Asian studies. Whereas one of these studies showed a clear positive association between red meat and T2D risk [38], the Shanghai Women’s Health Study showed an inverse association between red meat and T2D among normal weight women and an increased risk among obese women [39]. An important issue when interpreting these observations is the fact that in European and US- studies the mean intake of red meat is approximately 1.5 higher compared to Asian studies. It is possible that the red meat intake (especially among normal weight individuals) is not high enough to put participants at risk of T2D.

A recent meta-analysis showed that processed meat was associated with higher fasting glucose, and unprocessed red meat was associated with both higher fasting glucose and fasting insulin concentrations [40]. Whereas red and processed meat is positively associated with obesity [41], which may contribute to the causal pathway of T2D, the mechanism by which the consumption of meat may influence fasting glucose and insulin concentrations is more complex.

Similar to a previous meta-analysis we observed an inverse association of fruit and vegetable consumption and T2D [26]. Potential mechanistic evidence is mainly based on fiber content, which has been shown to improve insulin sensitivity and insulin secretion to overcome insulin resistance [32]. Moreover, vegetable and fruit intake indirectly influence T2D risk by preventing weight gain and risk of adiposity [42].

Several potential mechanisms are known for dairy products and T2D risk. There is some evidence that dairy products, especially those that are fermented, are associated with reduced risk of adiposity [43]. Moreover, in the EPIC-Interact study a strong inverse relation between saturated fatty acids, mainly present in dairy products and risk of T2D was observed [44].

In contrast to a non-significant linear association between eggs and T2D, we observed a 13% increased risk for approximately one serving/day in the non-linear meta-analysis. Moreover, there was a strong positive association between egg intake and risk of T2D among US- studies, whereas no association was observed for European and Asian studies. A possible explanation for this finding could be that in the US, eggs are frequently consumed with processed meat like bacon and sausages, which have been strongly associated with T2D risk.

Although two [45, 46] out of four US-reports have adjusted for red meat consumption, residual confounding by meat or other foods potentially associated with weight gain and risk of T2D may have affected the results.

A positive association between dietary cholesterol intake and risk of gestational diabetes was shown also in the Omega study [47]. There is some evidence that trimethylamine-N-oxide [48], a component of eggs, could be a mechanistic driver for the positive association between eggs and T2D [49]. However, there exist only limited data to support a biological mechanism that could explain this relation.

The results of the present meta-analysis add further scientific evidence supporting the inclusion of some food groups into food-based dietary guidelines for their potential role in preventing T2D. Optimal consumption of whole grains, vegetables, fruits, and dairy, and non-consumption of red meat, processed meat, SSB, and eggs was associated with an 81% reduced risk of T2D. Previous studies could show that diet plays an important role in T2D risk. A study from the EPIC-Potsdam cohort found occurrence of T2D to be particularly sensitive to lifestyle factors including diet compared to diseases such as coronary heart disease, stroke, or cancer [50]. One of the important findings of our study is that reducing the consumption of risk-increasing foods seems to have more impact on occurrence of T2D than favoring foods that reduce risk by increasing consumption. The impact on T2D risk was larger for non-consumption of red and processed meats, SSB, and eggs (threefold risk increase) compared to the consumption of whole grains, dairy, fruits, and vegetables (42% risk reduction). We also observed that the benefit of increasing consumption of risk-decreasing foods above a certain quantity is small to non-existing. With our results we can back the five a day campaign which will have manifold impact on disease occurrence [51] but could not strengthen suggestions for diets going beyond this recommendation.

Reduction of SSB offers one of the most important strategies to reduce the global burden of T2D. SSB can easily be replaced by water. In line with this approach are public health strategies to increase the cost for this food by levying tax or introducing sugar duty on SSB, which has been implemented in several countries including European countries, several states of the US, and Mexico [52]. Another strategy to reduce the incidence of T2D could be encouraging plant-based- or vegetarian diets (vegan, lacto-ovo, semi) [53]. In line with our observations the intake of red and processed meat should be as low as possible, although the mechanistic pathway is still unclear.

Specific dietary guidelines for the primary prevention of T2D by the American Diabetes Association include only one food-based recommendation: whole grains should constitute one-half of the total grain intake [54], whereas the most recent nutrition recommendations did not consider food-based recommendations for primary prevention. The results of the present study suggest that these recommendations might be expanded taking into consideration other food groups such as vegetables, fruits, and dairy whereas a reduced intake of red meat, processed meat, SSB, and eggs should be promoted in order to prevent T2D. It seems easier to put into practice guidelines based on food (groups) as compared to recommendations built upon percentage values for distinct macronutrients.

## Strengths and limitations

The present systematic review has several limitations. For some included studies, only baseline food intake was used (assuming a stable consumption over time). The included studies showed substantial heterogeneity with respect to the analyzed population size, follow-up length, baseline age, and food consumption. Moreover, only a few prospective studies reported nut intake, therefore the results should be interpreted with caution.

The strengths of the present meta-analysis include the a priori published systematic review protocol, the comprehensive literature search, the large number of included prospective studies and food groups, the different types of analyses (high vs. low, dose–response meta-analysis, and non-linear dose–response analysis), the calculated servings and its associated risk for T2D, and the application of the NutriGrade scoring system to assess the quality of meta-evidence for each food group. Because we based our analyses on prospective studies, we effectively avoided recall bias and reduced the potential for selection bias.

## Conclusion

Among the investigated food groups, selecting specific optimal intakes (by increasing whole grains, vegetables, fruits, and dairy; and reducing red and processed meats, SSB, and eggs) can lead to a considerable change in risk of T2D.

## Electronic supplementary material

Below is the link to the electronic supplementary material.

Supplementary material 1 (PDF 914 kb)
