# Supplementary material for: Food groups and risk of type 2 diabetes mellitus: a systematic review and meta-analysis of prospective studies
Source: Eur J Epidemiol. 2017 Apr 10;32(5):363–75. doi: 10.1007/s10654-017-0246-y (PMC5506108; doi:10.1007/s10654-017-0246-y)
Supplement: Supplementary file 1 — Supplementary material 1 (PDF 914 kb) [file 10654_2017_246_MOESM1_ESM.pdf]

Supplementary Appendix S1

#1 food*[tiab] OR whole grain*[tiab] OR refined grain*[tiab] OR cereal*[tiab] OR pasta*[tiab] OR rice*[tiab] OR potato*[tiab] OR vegetable*[tiab] OR fruit*[tiab] OR nut*[tiab] OR legume*[tiab] OR bean*[tiab] OR egg*[tiab] OR dairy[tiab] OR dairies[tiab] OR milk[tiab] OR yogurt[tiab] OR cheese[tiab] OR fish[tiab] OR seafood[tiab] OR meat[tiab] OR processed meat[tiab] OR sugar sweetened beverage*[tiab]

#2 diabetes

#3 prospective OR follow-up OR cohort OR longitudinal

#4 (#1 AND #2 AND #3)

Food group	Amount
Refined grains/whole grains	30 grams
Vegetables/fruits	80 grams
Nuts	28 grams
Legumes	100 grams
Eggs	55 grams
Dairy	200 grams
Fish	100 grams
Red meat	85 grams
Processed meat	30 grams
SSB	250 ml/grams

Supplementary Table S1: Conversion of 1 serving in grams

**Author**	**Year**	**Country**	**Cohort name**	**Age at entry**	**Sex**	**Sample size**	**Total cases**	**Dietary assessment**	**Outcome assessment**	**Type of whole grains**	**Adjustment factors**	**Follow up years**	**Results (high vs. low intake category), Risk ratio (RR)**
Ericson	2013	Sweden	MDC	45-74	Both	27140	1709	7 day menu book & SFFQ & interview	Registries	fiber rich soft or crisp bread and cereals	Age, season, energy intake, Body Mass Index, physical activity, smoking, Alcohol, Education	12	♀: 0.85 (0.68, 1.06)<br>♂: 0.84 (0.68, 1.04)
Hodge	2004	Australia	The Melbourne Collaborative Cohort Study	40-69	Both	31641	365	FFQ	self-reported/ doctor confirmation	whole meal bread	Sex, Age, Body Mass Index, waist: hip ratio, energy intake, education, alcohol, physical activity, weight change in the last 5 years, country of birth, family history of diabetes	4	0.86 (0.63, 1.18)
Kochar	2007	USA	PHS I	40-86	Men	21152	1958	abbreviated food questionnaire	self reported	whole grain cereals	Age, Body Mass Index, physical activity, smoking, Alcohol, vegetables, vitamin intake	19.1	0.60 (0.5, 0.71)
Lacoppidan	2015	Denmark	DCH	50-64	Both	55060	7366	SFFQ	registry	rye bread & oatmeal	Age, Study area, Body Mass Index, energy intake, smoking, alcohol,	15.3	♀:<br>Rye bread: 0.96 (0.88, 1.04)<br>Oatmeal:

											coffee, fiber, calcium, magnesium, vegetable, fruit, meat, rice, family history of diabetes, Hypertension, physical activity		0.90 (0.81, 0.99)<br>♂:<br>Rye bread: 0.89 (0.84, 0.96)<br>Oatmeal: 0.88 (0.81, 0.96)
Meyer	2000	USA	IWHS	55-69	Women	35988	1141	FFQ	self reported	whole grains	Age, energy intake, waist: hip ratio, Body Mass Index, physical activity, smoking, alcohol, education, dietary factors	6	0.93 (0.75, 1.15)
Montonen	2003	Finland	Finnish Mobile Clinic Health Examination Survey	40-69	Both	4316	156	Dietary history interview	registration to the Social Insurance Institution	whole grains	Age, Sex, area, energy intake, Body Mass Index, smoking, vegetables, fruit and berries	10	0.65 (0.36, 1.17)
Parker	2013	USA	WHI	50-79	Women	72215	3465	SFFQ	self reported	whole grains	Age, Race, energy intake, Body Mass Index, smoking, alcohol, hormone use, education income, family history of diabetes, physical activity, dairy, vegetables, fruit,	7.9	0.78 (0.64, 0.95)

											vitamin D, folic acid, Potassium, magnesium, fiber		
van Dam	2006	USA	Black Women's Health Study	21-69	Women	41186	1964	SFFQ	self reported	whole grains	Age, energy intake, Body Mass Index, Alcohol, physical activity, family history of diabetes, Smoking, Education, Coffee, Sugar-sweetened soft drink, red meat, processed meat, whole grain	8	0.69 (0.60, 0.79)
von Ruesten	2013	Germany	EPIC-Potsdam study	35-65	Both	23531	837	SFFQ	self-reported/inquiry to the physician	whole grain bread	Age, Sex, energy intake, Body Mass Index, waist: hip ratio, smoking, alcohol, physical activity, Hypertension, high blood lipid levels, education, vitamin supplementation	8	0.92 (0.85, 1.00)
Sun	2010	USA	HPFS	32-87	Men	39765	2648	SFFQ	self-reported/extra questionnaire/National Diabetes Data group criteria	whole grains	Age, ethnicity, Body Mass Index, physical activity, energy intake, smoking, alcohol, family history of diabetes,	20	0.72 (0.63, 0.82)

											multivitamin intake, red meat, fruits, vegetables, coffee		
Sun	2010	USA	NHS I	37-65	Women	69120	5500	SFFQ	self-reported/extra questionnaire/National Diabetes Data group criteria	whole grains	Age, ethnicity, Body Mass Index, physical activity, energy intake, smoking, alcohol, family history of diabetes, multivitamin intake, red meat, fruits, vegetables, coffee	22	0.70 (0.64, 0.77)
Sun	2010	USA	NHS II	26-45	Women	88343	2359	SFFQ	self-reported/extra questionnaire/National Diabetes Data group criteria	whole grains	Age, ethnicity, Body Mass Index, physical activity, energy intake, smoking, alcohol, family history of diabetes, multivitamin intake, red meat, fruits, vegetables, coffee	14	0.73 (0.68, 0.78)
Wirström	2013	Sweden	NA	35-56	Both	5206	165	SFFQ	OGTT/WHO criteria	whole grains	Age, Sex, education, blood pressure, physical activity, smoking, family history of diabetes	8-10	0.59 (0.41, 0.85)

Supplementary Table S2: General study characteristics of the included studies investigating the association between whole grain intake and risk of T2D;

Author	Year	Country	Cohort name	Age at entry	Sex	Sample size	Total cases	Dietary assessment	Outcome assessment	Type of refined grains	Adjustment factors	Follow up years	Results (high vs. low intake category) Risk ratio (RR)
Ericson	2013	Sweden	MDC	45-74	Both	27140	1709	7 day menu book & SFFQ & interview	Registries	refined cereals	Age, season, energy intake, Body Mass Index, physical activity, smoking, Alcohol, Education	12	♀: 1.07 (0.87, 1.32) ♂: 1.02 (0.82, 1.27)
Fung	2002	USA	HPFS	40-75	Men	42898	1197	SFFQ	self-reported/extra questionnaire/National Diabetes Data group criteria	refined grains	Age, period, missing food frequency questionnaire, energy intake, Body Mass Index, physical activity, smoking, Alcohol, family history of diabetes, fruit, vegetables	≤12	1.08 (0.87, 1.34)
Golozar	2017	Iran	GCS	40-87	Both	50045	902	FFQ	Participants who had FPG ≥126 mg/dl, HbA1c >6.5% or were receiving treatment for diabetes	white rice	Age, sex, wealth score, education, matrial status, opium, alcohol, physical activity, smoking, energy	5	0.90 (0.73, 1.12)

											intake, meat, employment status,		
Golozar	2017	Iran	TLGS	39	Both	2887	81	FFQ	Participants who had FPG ≥126 mg/dl, or 2h plasma glucose ≥ 200 mg/dl during OGTT receiving treatment anti-diabetic medications	white rice	Age, sex, family history of diabetes, education, matrial status, physical activity, smoking, energy intake, meat, employment status	3	2.28 (1.19, 4.37)
Hodge	2004	Australia	The Melbourne Collaborative Cohort Study	40-69	Both	31641	365	FFQ	self-reported/ doctor confirmation	white bread, white pasta	Sex, Age, body Mass Index, waist: hip ratio, energy intake, education, alcohol, physical activity, weight change in the last 5 years, country of birth, family history of diabetes	4	white bread: 1.13 (0.86, 1.5) white pasta: 0.86 (0.6, 1.23)
Liu	2000	USA	NHS	38-63	Women	75521	1879	SFFQ	self-reported/extra questionnaire/National Diabetes Data group criteria	refined grains	Age, Body Mass Index, physical activity, energy intake, smoking, alcohol, family history of diabetes, vitamin E or multivitamin intake	10	1.11 (0.94, 1.31)
Meyer	2000	USA	IWHS	55-69	Women	35988	1141	FFQ	self reported	refined grains	Age, energy inatke, waist: hip ratio, Body Mass	6	0.87 (0.70, 1.08)

											Index, physical activity, smoking, alcohol, education		
Montonen	2003	Finland	Finnish Mobile Clinic Health Examination Survey	40-69	Both	4316	156	Dietary history interview	registration to the Social Insurance Institution	refined grains	Age, Sex, Area, energy intake, Body Mass Index, smoking, vegetables, fruit and berries	10	0.62 (0.36, 1.07)
Nanri	2010	Japan	JPHC	45-75	Both	59288	1103	SFFQ	self reported	rice cooked	Age, Area, energy intake, Body Mass Index, smoking, alcohol, family history of diabetes, physical activity, hypertension, occupation, vegetables, fruit, coffee, calcium, magnesium, fish, fiber, bread, noodle	5	♂: Rice cooked: 1.19 (0.85, 1.68) Bread: 0.85 (0.64, 1.14) Noodle: 0.89 (0.68, 1.17)<br><br>♀: Rice cooked: 1.65 (1.06, 2.57) Bread: 0.99 (0.73, 1.34) Noodle:

													1.15 (0.83, 1.58)
Parker	2013	USA	WHI	50-79	Women	72215	3465	SFFQ	self reported	refined grains	Age, Race, energy intake, Body Mass Index, smoking, alcohol, hormone use, education income, family history of diabetes, physical activity, dairy, vegetables, fruit, vitamin D, folic acid, potassium, magnesium, fiber	7.9	0.77 (0.61, 0.97)
Soriguer	2013	Spain	The Pizzara study		Both	605	54	FFQ	Fasting blood sample and OGTT/WHO criteria	White rice	Age, Sex, abnormal glucose regulation at baseline, obesity, carbohydrate consumption	6	0.41 (0.17, 0.99)
Sun	2010	USA	HPFS	32-87	Men	39765	2648	SFFQ	self-reported/extra questionnaire/National Diabetes Data group criteria	white rice (uncooked)	Age, ethnicity, Body Mass Index, physical activity, energy intake, smoking, alcohol, family history of diabetes, multivitamin intake, red meat, fruits, vegetables, whole grains,	20	1.02 (0.77, 1.35)

											coffee		
Sun	2010	USA	NHS I	37-65	Women	69120	5500	SFFQ	self-reported/extra questionnaire/National Diabetes Data group criteria	white rice (uncooked)	Age, ethnicity, Body Mass Index, physical activity, energy intake, smoking, alcohol, family history of diabetes, multivitamin intake, red meat, fruits, vegetables, whole grains, coffee, postmenopausal status, hormone use, oral contraceptive	22	1.11 (0.87, 1.42)
Sun	2010	USA	NHS II	26-45	Women	88343	2359	SFFQ	self-reported/extra questionnaire/National Diabetes Data group criteria	white rice (uncooked)	Age, ethnicity, Body Mass Index, physical activity, energy intake, smoking, alcohol, family history of diabetes, multivitamin intake, red meat, fruits, vegetables, whole grains, coffee, postmenopausal status, hormone use, oral contraceptive	14	1.40 (1.09, 1.80)

Supplementary Table S3: General study characteristics of the included studies investigating the association between refined grain intake and risk of T2D.

Author	Year	Country	Cohort name	Age at entry	Sex	Sample size	Total cases	Dietary assessment	Outcome assessment	Type of vegetables	Adjustment factors	Follow up years	Results (high vs. low intake category) Risk ratio (RR)
Bazzano	2008	USA	NHS	38-63	Women	71346	4529	SFFQ	self-reported/extra questionnaire/National Diabetes Data group criteria	vegetables	Age, energy intake, Body Mass Index, family history of diabetes, postmenopausal hormone use, smoking, alcohol, physical activity, whole grains, nuts, processed meats, coffee, potatoes, sugar sweetened soft drinks	18	1.05 (0.94, 1.17)
Cooper	2013	Europe	EPIC-InterAct	52.3	Both	16154	10821	Country specific questionnaires	self reported or registers or mortality data	vegetables	Age, Sex, country, center, education, energy intake, Body Mass Index, smoking, alcohol, physical activity, vegetables	3.99 million person-years	0.94 (0.84, 1.05)
Hodge	2004	Australia	The Melbourne Collaborative Cohort Study	40-69	Both	31641	365	FFQ	self-reported/ doctor confirmation	vegetables	Sex, Age, Body Mass Index, waist: hip ratio, energy intake, education, alcohol, physical activity, weight change in the last 5	4	0.88 (0.60, 1.29)

											years, country of birth, family history of diabetes		
Kurotani	2013	Japan	JPHC	45-75	Both	48437	896	SFFQ	self reported	vegetables	Age, energy intake, Body Mass Index, smoking, alcohol, physical activity, area, hypertension, family history of diabetes, coffee, magnesium, calcium	5	♂ 0.81 (0.59, 1.11) ♀ 0.99 (0.66, 1.49)
Lacoppidan	2015	Denmark	DCH	50-64	Both	55060	7366	SFFQ	registry	root vegetable, cabbage	Age, Study area, Body Mass Index, energy intake, smoking, alcohol, coffee, fiber, calcium, magnesium, vegetable, fruit, meat, rice, family history of diabetes, Hypertension, physical activity	15.3	♀: Cabbage: 0.99 (0.92, 1.06) Root vegetables: 0.94 (0.87, 1.02) ♂: Cabbage: 0.92 (0.86, 0.99) Root vegetables: 0.98 (0.91, 1.05)
Liu	2004	USA	WHS	≥45	Women	38018	1614	SFFQ	self-reported/ADA criteria	vegetables	Age, energy intake, Body Mass Index, smoking, alcohol, physical activity, hypertension, hypercholesterolemia, family history of diabetes	8.8	1.03 (0.86, 1.23)

Mamluk	2016	USA	NIH-AARP	>50	Both	401909	22782	FFQ	self-administered questionnaires or in interviews	vegetables	age, sex, Body mass Index, physical activity, energy intake, alcohol consumption, education, smoking	10.6	0.92 (0.87, 0.97)
Mamluk	2016	Europe	EPIC Greece	>50	Both	20629	1077	24 h recall	self-administered questionnaires or in interviews	vegetables	age, sex, Body Mass Index, physical activity, energy intake, alcohol consumption, education, smoking	10	2.15 (0.93, 4.97)
Meyer	2000	USA	IWHS	55-69	Women	35988	1141	FFQ	self reported	vegetables	Age, energy intake, Body Mass Index, waist: hip ratio, education, smoking, alcohol, physical activity	6	1.07 (0.86, 1.33)
Montonen	2005	Finland	Finnish Mobile Clinic Health Examination Survey	40-69	Both	4304	383	Dietary history interview	registration to the Social Insurance Institution	vegetables	Age, Sex, energy intake, Body Mass Index, smoking, family history of diabetes, Geographic area	23	0.77 (0.57, 1.04)
Mursu	2014	Finland	KIHD	42-60	Men	2332	432	4 day food recording	self reported and diabetes register and blood glucose measurements and OGTT	vegetables	Age, examination years, waist: hip ratio, education, energy intake, Body Mass Index, family history of diabetes, smoking, alcohol, physical activity	19.3	0.81 (0.61, 1.08)
Qiao	2014	USA	WHI	50-79	Women	154493	10285	FFQ	self reported	vegetables	Age, energy intake, Body Mass Index,	7.6	1.10 (0.96, 1.26)

											Waist: hip ratio, education, smoking, physical activity, family history of diabetes, study arms, hormone therapy use		
Villegas	2008	China	SWHS	40-70	Women	64191	1608	FFQ	self-reported/ADA criteria	vegetables	Age, energy intake, Body Mass Index, waist: hip ratio, education, smoking, alcohol, physical activity, vegetable and fiber intake, income, occupation, hypertension	4.6	0.72 (0.61, 0.85)

Supplementary Table S4: General study characteristics of the included studies investigating the association between vegetable intake and risk of T2D.

**Author**	**Year**	**Country**	**Cohort name**	**Age at entry**	**Sex**	**Sample size**	**Total cases**	**Dietary assessment**	**Outcome assessment**	**Type of fruits**	**Adjustment factors**	**Follow up years**	**Results (high vs. low intake category) Risk ratio (RR)**
Cooper	2013	Europe	EPIC-InterAct	52.3	Both	16154	10821	Country specific questionnaires	self reported or registers or mortality data	fruit	Age, Sex, country, center, education, energy intake, Body Mass Index, smoking, alcohol, physical activity, vegetables	3.99 million person-years	0.89 (0.76, 1.04)
Hodge	2004	Australia	The Melbourne Collaborative Cohort Study	40-69	Both	31641	365	FFQ	self-reported/ doctor confirmation	fruit	Sex, Age, Body Mass Index, waist: hip ratio, energy intake, education, alcohol, physical activity, weight change in the last 5 years, country of birth, family history of diabetes	4	0.85 (0.59, 1.22)
Kurotani	2013	Japan	JPHC	45-75	Both	48437	896	SFFQ	self reported	fruit	Age, energy intake, Body Mass Index, smoking, alcohol, physical activity, area, hypertension, family history of diabetes, coffee, magnesium, calcium	5	♂: 0.94 (0.71, 1.24)<br><br>♀ 1.04 (0.73, 1.48)

Lacoppidan	2015	Denmark	DCH	50-64	Both	55060	7366	SFFQ	registry	apples and bears	Age, Study area, Body Mass Index, energy intake, smoking, alcohol, coffee, fiber, calcium, magnesium, vegetable, fruit, meat, rice, family history of diabetes, Hypertension, physical activity	15.3	♀: 1.03 (0.96, 1.11) ♂: 0.97 (091, 1.04)
Liu	2004	USA	WHS	≥45	Women	38018	1614	SFFQ	self-reported/ADA criteria	fruit	Age, energy intake, Body Mass Index, smoking, alcohol, physical activity, hypertension, hypercholesterolemia, family history of diabetes	8.8	0.97 (0.82, 1.15)
Mamluk	2016	USA	NIH-AARP	>50	Both	401909	22782	FFQ	self-administered questionnaires or in interviews	fruit	age, sex, Body Mass Index, physical activity, energy intake, alcohol consumption, education, smoking	10.6	0.95 (0.91, 0.99)
Mamluk	2016	Europe	EPIC Greece	>50	Both	20629	1077	26 h recall	self-administered questionnaires or in interviews	fruit	age, sex, Body Mass Index, physical activity, energy intake, alcohol consumption, education, smoking	10	1.09 (0.77, 1.54)

Meyer	2000	USA	IWHS	55-69	Women	35988	1141	FFQ	self reported	fruit	Age, energy intake, Body Mass Index, waist: hip ratio, education, smoking, alcohol, physical activity	6	1.14 (0.93, 1.40)
Montonen	2005	Finland	Finnish Mobile Clinic Health Examination Survey	40-69	Both	4304	383	Dietary history interview	registration to the Social Insurance Institution	fruit & berries	Age, Sex, energy intake, Body Mass Index, smoking, family history of diabetes, Geographic area	23	0.69 (0.51, 0.93)
Muraki	2013	USA	NHS	48.2	Women	66105	6358	SFFQ	self reported/extra questionnaire/National Diabetes Data group criteria or death reports	whole fruit	Age, ethnicity, energy intake, Body Mass Index, smoking, multivitamin use, fruit juice, physical activity, area, family history of diabetes, modified alternate healthy index score, menopausal status, post-menopausal hormone use	1394127 person years	0.99 (0.82, 1.20)
Muraki	2013	USA	NHS II	35.9	Women	85104	3153	SFFQ	self-reported/extra questionnaire/National Diabetes Data group criteria or death reports	whole fruit	Age, ethnicity, energy intake, Body Mass Index, smoking, multivitamin use, fruit juice, physical activity, area, family history of diabetes, modified alternate healthy index score,	1416111 person years	0.82 (0.72, 0.93)

											menopausal status, post-menopausal hormone use, oral contraceptive		
Muraki	2013	USA	HPFS	49.9	Men	36173	2687	SFFQ	self-reported/extra questionnaire/National Diabetes Data group criteria or death reports	whole fruit	Age, ethnicity, energy intake, Body Mass Index, smoking, multivitamin use, fruit juice, physical activity, area, family history of diabetes, modified alternate healthy index score	654403 person yaers	0.92 (0.78, 1.09)
Mursu	2014	Finland	KIHD	42-60	Men	2332	432	4 day food recording	self reported and diabetes register and blood glucose measurements and OGTT	whole fruit	Age, examination years, waist: hip ratio, education, energy intake, Body Mass Index, family history of diabetes, smoking, alcohol, physical activity	19.3	0.98 (0.75, 1.28)
Qiao	2014	USA	WHI	50-79	Women	154493	10285	FFQ	self reported	fruit	Age, energy intake, Body Mass Index, waist: hip ratio, education, smoking, physical activity, family history of diabetes, study arms, hormone therapy use	7.6	0.99 (0.93, 1.05)
Villegas	2008	China	SWHS	40-70	Women	64191	1608	FFQ	self-reported/ADA criteria	fruit	Age, energy intake, Body Mass Index,	4.6	1.05 (0.90,

											waist: hip ratio, education, smoking, alcohol, physical activity, vegetable and fiber intake, income, occupation, hypertension		1.22)

Supplementary Table S5: General study characteristics of the included studies investigating the association between fruit intake and risk of T2D.

Author	Year	Country	Cohort name	Age at entry	Sex	Sample size	Total cases	Dietary assessment	Outcome assessment	Type of nuts	Adjustment factors	Follow up years	Results (high vs. low intake category) Risk ratio (RR)
Asghari	2017	Iran	TLGS	≥20	Both	1984	150	FFQ	Participants who had FPG ≥126 mg/dl, or 2h plasma glucose ≥ 200 mg/dl during OGTT receiving treatment anti-diabetic medications	nuts	Sex, education, smoking, BMI, blood pressure, cholesterol, triacylglycerols, vegetables, fruits, red meat, fish, poultry, total sugar, fiber, fats, carbohydrates, protein	6.2	0.47 (0.25, 0.90)
Buijsse	2015	Europe	EPIC-InterAct	52.3	Both	16835	12403	24h recall	self reported or registers or mortality data	nuts & seeds	Centre, Sex, education, energy intake, Body Mass Index, smoking, alcohol, physical activity	3.99 million years of follow-up	0.88 (0.75, 1.03)
Kochar	2010	USA	PHS I	40.7-87.1	Men	20224	1828	SFFQ	self reported	nuts	Age, randomization arm, Body Mass Index, smoking, alcohol, physical activity, breakfast cereal, dairy, red meat, hypertension	19.2	0.87 (0.61, 1.24)
Pan	2013	USA	NHS	52-77	Women	59259	5121	SFFQ	self-reported/extra questionnaire/National	nuts	Age, energy intake, Body	10	1.00 (0.87, 1.15)

									Diabetes Data group criteria		Mass Index, race, family history of diabetes, smoking, alcohol, physical activity, postmenopausal status, menopausal hormone use, whole grains, multivitamin use, whole grains, vegetables, fruits, fish, red meat, coffee, sugar sweetened beverages		
Pan	2013	USA	NHS II	35-52	Women	91799	4098	SFFQ	self-reported/extra questionnaire/National Diabetes Data group criteria	nuts	Age, energy intake, Body Mass Index, race, family history of diabetes, smoking, alcohol, physical activity, postmenopausal status, menopausal hormone use, whole grains, multivitamin use, whole grains, vegetables, fruits, fish, red meat,	10	1.02 (0.85, 1.22)

											coffee, sugar sweetened beverages		
Parker	2003	USA	IWHS	11	Women	35988	1831	FFQ	self-reported/ criteria	nuts	Age, Body Mass Index, waist: hip ratio, physical activity Smoking, Alcohol, Dairy, Education, Estrogen use, fiber, polyunsaturated fat, saturated fat, monounsaturated fat, trans, fruit, vegetables, whole grains, fish, sea food, magnesium	11	1.51 (1.13, 2.02)
Villegas	2008	China	SWHS	40-70	Women	64227	1605	FFQ	self-reported/ criteria	peanuts	Age, energy intake, Body Mass Index, waist: hip ratio, education, smoking, alcohol, physical activity, vegetable and fiber intake, income, occupation, hypertension	4.6	0.80 (0.68, 0.94)
von Ruesten	2013	Germany	The EPIC-	35-65	Both	23531	837	SFFQ	self-reported/inquiry to the physician	nuts	Age, Sex, energy intake, Body	8	0.95 (0.87, 1.06)

			Potsdam study								Mass Index, waist: hip ratio, smoking, alcohol, physical activity, Hypertension, high blood lipid levels, education, vitamin supplementation		

Supplementary Table S6: General study characteristics of the included studies investigating the association between nut intake and risk of T2D.

Author	Year	Country	Cohort name	Age at entry	Sex	Sample size	Total cases	Dietary assessment	Outcome assessment	Type of legumes	Adjustment factors	Follow up years	Results (high vs. low intake category) Risk ratio (RR)
Bazzano	2008	USA	NHS	38-63	Women	71346	4529	SFFQ	self-reported/extra questionnaire/National Diabetes Data group criteria	legumes	Age, energy intake, Body Mass Index, family history of diabetes, postmenopausal hormone use, smoking, alcohol, physical activity, whole grains, nuts, processed meats, coffee, potatoes, sugar sweetened soft drinks	18	1.14 (1.03, 1.26)
Ding	2016	USA	NHS	30-54	Women	63115	4519	FFQ	Self-reported incident T2D was mailed a validated supplementary questionnaire regarding symptoms, diagnostic tests and hypoglycemic therapy to confirm the diagnosis of diabetes. Cases were ascertained using the American Diabetes Association criteria	Soy	race, family history of diabetes, baseline disease status: hypertension, hypercholesterolemia; Body Mass Index, physical activity, overall dietary pattern (alternate Healthy eating Index), total energy intake, coffee consumption, smoking status, menopausal status, and postmenopausal	1966321 person years	0.88 (0.66, 1.17)

											hormone use		
Ding	2016	USA	NHS II	30-54	Women	79061	3920	FFQ	Self-reported incident T2D was mailed a validated supplementary questionnaire regarding symptoms, diagnostic tests and hypoglycemic therapy to confirm the diagnosis of diabetes. Cases were ascertained using the American Diabetes Association criteria	Soy	race, family history of diabetes, baseline disease status: hypertension, hypercholesterolemia, body mass index, physical activity, overall dietary pattern (alternate Healthy eating Index), total energy intake, coffee consumption, smoking status, menopausal status, and postmenopausal hormone use	1966321 person years	0.97 (0.84, 1.12)
Ding	2016	USA	HPFS	30-54	Men	21281	742	FFQ	Self-reported incident T2D was mailed a validated supplementary questionnaire regarding symptoms, diagnostic tests and hypoglycemic therapy to confirm the diagnosis of diabetes. Cases were ascertained using the American Diabetes Association criteria	Soy	race, family history of diabetes, baseline disease status: hypertension, hypercholesterolemia, Body Mass Index, physical activity, overall dietary pattern (alternate Healthy eating Index), total energy intake, coffee consumption, smoking status, menopausal status, and postmenopausal hormone use	1966321 person years	0.92 (0.80, 1.06)
Meyer	2004	USA	WHS	55-69	Women	35988	1141	FFQ	self reported	mature beans	Age, energy intake, Body Mass Index,	6	0.96 (0.76,

											waist: hip ratio, education, smoking, alcohol, physical activity		1.21)
Morimoto	2011	USA	MEC	45-75	Both	75344	8564	FFQ	Registries & Self reported	soy	Body Mass Index, Ethnicity physical activity, education, total energy, smoking status, alcohol, dietary fiber and processed red meat intake	14	♂: 1.18 (1.09, 1.28)<br><br>♀: 1.18 (1.09, 1.28)
Mueller	2012	Australia	SCHS	45-74	Both	43176	2252	SFFQ	Self reported	unsweetened soy	Body Mass Index, age, sex, dialect, year of interview, educational level, smoking status , alcohol, physical activity , baseline hypertensive status, rice, noodles, other grains, red meat, green vegetable, soybean drink, and energy intake	5.7	0.72 (0.59, 0.88)
Nanri	2010	Japan	JPHS	45-75	Both	59791	1114	SFFQ	self reported	soy products	age, study area, Body Mass Index, smoking, alcohol, family history of diabetes mellitus, leisure time physical activity, history of hypertension, coffee consumption, green tea consumption, magnesium intake,	5	♂: 1.02 (0.75, 1.39)<br><br>♀: 0.98 (0.70, 1.37)

											calcium intake, vegetable intake, fiber intake, fish intake, and energy intake		
Villegas	2008	China	SWHS	40-70	Women	64227	1605	FFQ	self reported	legumes	Age, energy intake, Body Mass Index, waist: hip ratio, education, smoking, alcohol, physical activity, vegetable and fiber intake, income, occupation, hypertension	4.6	0.62 (0.51, 0.75)
von Ruesten	2013	Germany	The EPIC-Potsdam study	35-65	Both	23531	837	SFFQ	self-reported/inquiry to the physician	legumes	Age, Sex, energy intake, Body Mass Index, waist: hip ratio, smoking, alcohol, physical activity, Hypertension, high blood lipid levels, education, vitamin supplementation	8	1.01 (0.96, 1.06)

Supplementary Table S7: General study characteristics of the included studies investigating the association between legume intake and risk of T2D.

Author	Year	Country	Cohort name	Age at entry	Sex	Sample size	Total cases	Dietary assessment	Outcome assessment	Type of eggs	Adjustment factors	Follow up years	Results (high vs. low intake category) Risk ratio (RR)
Djousse	2009	USA	PHS I	≥40	Men	20703	1921	SFFQ	Self reported	eggs	Age, energy intake, Body Mass Index, smoking, alcohol, physical activity, Hypertension, Hypercholesterolemia, family history of diabetes, red meat, fruits, vegetables, saturated fat, trans, polyunsaturated	20	1.58 (1.25, 2.00)
Djousse	2009	USA	WHS	≥45	Women	36295	2112	SFFQ	Self reported	eggs	Age, energy intake, Body Mass Index, smoking, alcohol, physical activity, Hypertension, Hypercholesterolemia, family history of diabetes, red meat, fruits, vegetables, saturated fat, trans, polyunsaturated	11.7	1.77 (1.28, 2.45)
Djousse	2010	USA	CHS	≥65	Both	3898	313	picture sorted food questionnaire	annual medication assessment and fasting glucose measurement	eggs	Age, race, Body Mass Index, smoking, alcohol, physical	11.3	♂ 1.81 (0.77,

											activity, cereal-fiber, field center		4.25)<br>♀<br>0.38<br>(0.10,<br>1.44)
Djousse	2015	USA	Jackson Heart Study	55±13	Both	4568	1004	FFQ	NA	eggs	age, sex, smoking, alcohol, Body Mass Index, physical activity score, education, energy intake, red meat, fiber, dietary magnesium, fruit/vegetables, trans fat, waist circumference, history of hypertension, history of cardiovascular disease	7.3	1.17<br>(0.81,<br>1.69)
Ericson	2015	Sweden	MDC	45-74	Both	26930	2860	7 day menu book & SFFQ & interview	Registries or follow up examination	eggs	Age, Sex, season, method version, energy intake, Body Mass Index, education, smoking, alcohol, physical activity	14	1.14<br>(1.02,<br>1.27)
Kurotani	2014	Japan	JPHC	45-75	Both	63466	1165	SFFQ	self reported	eggs	Age, center area, energy intake, smoking, alcohol, Hypertension, family history of diabetes, physical activity, Body Mass Index, Magnesium, Calcium, Coffee, rice, fish, shellfish, meat,	5	♂<br>1.06<br>(0.85,<br>1.32)<br><br>♀<br>0.82<br>(0.63,<br>1.07)

											vegetables, soft drinks		
Lajous	2015	France	E3N	43-68	Women	65364	1803	FFQ	selfreported/supplementary questionnaire or drug information	eggs	Age, energy intake, education, Body Mass Index, smoking, alcohol, physical activity, hormone replacement therapy, Hypertension, Hypercholesterolemia, family history of diabetes, processed red meat, fruits, vegetables, coffee, sugar sweetened drinks, artificially sweetened drinks	14	1.00 (0.78, 1.28)
Montonen	2005	Finland	Finnish Mobile Clinic Health Examination Survey	40-69	Both	4304	383	Dietary history interview	registration to the Social Insurance Institution	eggs	Age, Sex, energy intake, Body Mass Index, smoking, family history of diabetes, geographic area	23	0.91 (0.67, 1.24)
Vang	2008	USA	AMS & AHS	45-88	Both	8401	535	FFQ	self-reported	eggs	Age, Sex	17	1.15 (0.85, 1.56)
Virtanen	2015	Finland	KIHD	42-60	Men	2332	432	4-d food records	selfreported or hospital and insurance registration/ WHO criteria	eggs	Age, energy intake, examination year, education, Body Mass Index, smoking, alcohol, physical activity, hypertension family history of diabetes, serum long chain omega-3 polyunsaturated fatty	19.3	0.55 (0.38, 0.80)

											acids, fiber, linoleic acid, berries, fruits, vegetables, cholesterol intake		
von Ruesten	2013	Germany	The EPIC-Potsdam study	35-65	Both	23531	837	SFFQ	selfreported/inquiry to the physician	eggs	Age, Sex, energy intake, Body Mass Index, waist: hip ratio, smoking, alcohol, physical activity, Hypertension, high blood lipid levels, education, vitamin supplementation	8	0.97 (0.75, 1.25)
Wallin	2016	Sweden	COSM	45-79	Men	39610	4173	FFQ	registry	eggs	Age, Body Mass Index, energy intake, education, smoking, alcohol, physical activity, cardiovascular disease, coffee, red meat, processed meat, fish, fruit, vegetables, white bread, caviar, sweet buns/biscuits, fiber	15	1.11 (0.95, 1.30)
Zazpe	2013	Spain	SUN project	20-90	Both	15956	91	SFFQ	selfreported/medical records confirmation	eggs	Age, Sex, energy intake, Mediterranean food pattern, Alcohol, Body Mass Index, Smoking, physical activity, family history of diabetes, history of hypertension , history of	6.6	0.70 (0.30, 1.21)

											hypercholesterolemia		

Supplementary Table S8: General study characteristics of the included studies investigating the association between egg intake and risk of T2D.

Author	Year	Country	Cohort name	Age at entry	Sex	Sample size	Total cases	Dietary assessment	Outcome assessment	Type of dairy	Adjustment factors	Follow up years	Results (high vs. low intake category) Risk ratio (RR)
Brouwer-Brolsma	2016	Netherlands	Rotterdam study	>55	Both	7983	393	SFFQ	ascertained using records of general practioners, hospital discharge letters, and serum glucose measurements from Rotterdam study visits taking place every 4 years	Total dairy	age, sex, alcohol, smoking, education, physical activity, Body Mass Index, total energy intake, energy adjusted meat intake, energy adjusted fish intake, and potential intermediates: total cholesterol, HDL-cholesterol, C-reactive protein, hypertension	9.5	0.93 (0.69, 1.25)
Chen	2014	USA	HPFS	40-75	Men	41436	3364	SFFQ	self-reported/extra questionnaire/National Diabetes Data group criteria or death reports	Total dairy	Age, energy intake, Body Mass Index, Alcohol, physical activity, family history of diabetes, Smoking, race, history of Hypertension and/or Hypercholesterolemia, oral contraceptive use, trans fat, glycemic load, red & processed meat, nuts sugar sweetened beverages,	24	0.99 (0.77, 1.11)

											coffee		
Chen	2014	USA	NHS I	30-55	Women	67138	7841	SFFQ	self-reported/extra questionnaire/National Diabetes Data group criteria or death reports	Total dairy	Age, energy intake, Body Mass Index, Alcohol, physical activity, family history of diabetes, Smoking, race, history of hypertension and/or hypercholesterolemia, Menopausal status, menopausal hormone use, oral contraceptive use, trans fat, glycemic load, red & processed meat, nuts sugar sweetened beverages, coffee	30	1.05 (0.97, 1.14)
Chen	2014	USA	NHS II	25-42	Women	85884	3951	SFFQ	self-reported/extra questionnaire/National Diabetes Data group criteria or death reports	Total dairy	Age, energy intake, Body Mass Index, Alcohol, physical activity, family history of diabetes, Smoking, race, history of hypertension and/or hypercholesterolemia ,Menopausal status, menopausal hormone use, oral contraceptive use, trans fat, glycemic load, red & processed meat, nuts sugar sweetened beverages, coffee	16	1.00 (0.89, 1.12)
Diaz-Lopez	2015	Spain	PREDIMED	55-80	Both	3454	270	SFFQ	Medical records review or routine	Total dairy	Age, Sex, Body Mass Index, intervention,	4.1	0.68 (0.47,

									glucose test		education, physical activity, hypertension or antihypertensive use, fasting glucose, HDL-C, triacylglycerol's, smoking, alcohol, vegetables, legumes, fruits, cereals, meat, fish, olive oil, nuts		0.98)
Elwood	2007	UK	Caerphilly prospective study	45-59	Men	640	41	7-day weighed record	self reported	milk	Age, Body Mass Index, Smoking, social class	20	0.57 (0.20, 1.62)
Ericson	2015	Sweden	MDC	45-74	Both	26930	2860	7 day menu book & SFFQ & interview	Registries or follow up examination	Total dairy	Age, Sex, season, method version, energy intake, Body Mass Index, education, smoking, alcohol, physical activity	14	0.90 (0.80, 1.01)
Grantham	2012	Australia	AusDiab	25-88	Both	5582	209	SFFQ	treatment reporting or fasting plasma glucose or 2h post-load plasma glucose	Total dairy	Age, Sex, energy intake, education, family history of diabetes, smoking, triacylglycerol's, physical activity, systolic blood pressure, waist circumference, HDL-C	5	0.71 (0.48, 1.05)
Kirrii	2009	Japan	JPHC	40-69	Both	59796	1114	SFFQ	self reported	Total dairy	Age, energy intake, Body Mass Index, smoking, alcohol, family history of diabetes,	5	♂ 1.18 (0.90, 1.55)

											Hypertension, physical activity, area, coffee, magnesium		♀ 0.71 (0.51, 0.99)
Liu	2006	USA	WHS	NA	Women	37183	1603	SFFQ	self-reported/ phone call or extra questionnaire/ADA diagnostic criteria or contacting physician	Total dairy	Age, energy intake, Body Mass Index, Alcohol, physical activity, family history of diabetes, Hypercholesterolemia, Hypertension, Hormones, Smoking, fiber, magnesium, glycemic load, total fat, calcium, vitamin D	10	0.68 (0.52, 0.89)
Louie	2013	Australia	BMES	≥49	Both	1824	145	SFFQ	self reported	Total dairy	Age, Sex, Body Mass Index, systolic blood pressure, HDL-C, total-cholesterol, triacylglycerol's, calcium	10	1.50 (0.47, 4.79)
Margolis	2011	USA	WHI-OS	50-79	Women	82076	3946	SFFQ	self reported	Total dairy	Age, energy intake, Body Mass Index, race, education, income, family history of diabetes, postmenopausal hormone therapy, smoking, alcohol, physical activity, blood pressure, glycemic load, fat, fiber, magnesium	7,9	0.93 (0.83, 1.04)
Montonen	2005	Finland	Finnish	40-	Both	4304	383	Dietary	registration to the	Total	Age, Sex, energy	23	0.90

			Mobile Clinic Health Examination Survey	69				history interview	Social Insurance Institution	dairy	intake, Body Mass Index, smoking, family history of diabetes, Geographic area		(0.74, 1.09)
Moslehi	2015	Iran	TLGS	≥20	Both	520	178	FFQ	Participants who had FPG ≥126 mg/dl, or 2h plasma glucose ≥ 200 mg/dl during OGTT receiving treatment anti-diabetic medications	Total dairy	Age, sex, family history of diabetes, BMI, waist circumference, energy intake, blood pressure, triacyglycerols, cholesterol	NA	0.73 (0.47, 1.16)
Sluijs	2012	Europe	EPIC-Interact Study	52	Both	16835	12405	SFFQ or quantitative dietary questionnaire	self reported or care registers or medication use, mortality data	Total dairy	Age, Sex, Body Mass Index, energy intake, education, smoking, alcohol, physical activity, fruit, vegetables, red meat, processed meat, sugar sweetened soft drinks, coffee, cereals, calcium, magnesium , vitamin D	3.99 million person years	0.97 (0.82, 1.15)
Soedamah-Muthu	2012	UK	Whitehall II	NA	Both	4186	273	FFQ	self reported or glucose tolerance test	Total dairy	Sex, Age, energy intake, Body Mass Index, ethnicity, employment grade, smoking, alcohol, family history of coronary heart disease and/or hypertension, physical activity, coffee, fruit, vegetables, bread, meat, fish, tea	10	1.30 (0.95, 1.78)

Struijk	2013	Denmark	Inter99	30-60	Both	5232	214	FFQ	health examination/WHO definition	Total dairy	Age, Sex, Intervention group, family history of diabetes, waist: hip ratio, energy intake, education, smoking, alcohol, physical activity, wholegrain cereal, meat, fish, fruit, vegetables, tea, coffee, change in diet during follow-up	5	0.95 (0.86, 1.05)
van Dam	2006	USA	Black Women's Health Study	21-69	Women	41186	1964	SFFQ	selfreported	Total dairy	Age, energy intake, Body Mass Index, Alcohol, physical activity, family history of diabetes, Smoking, Education, Coffee, Sugar-sweetened soft drink, red meat, processed meat, whole grain	8	0.93 (0.75, 1.15)
Vang	2008	USA	AMS & AHS	45-88	Both	8401	543	FFQ	selfreported	milk	Age, Sex	17	0.91 (0.75, 1.10)
Villegas	2009	China	SWHS	40-70	Women	64191	2270	FFQ	selfreported/ADA criteria	fresh milk	Age, energy intake, Body Mass Index, WHR, smoking, alcohol, education, income, occupation, Hypertension, physical activity	6,9	0.72 (0.65, 0.80)
Zong	2014	China	Nutrition and Health of Aging Population	50-70	Both	2091	507	SFFQ	health examination	Total dairy	Age, Sex, region, residence, family history of diabetes, Body Mass Index,	6	0.81 (0.63, 1.04)

			in China study								smoking, fiber, changes in Body Mass Index, waist circumference and glucose		

Supplementary Table S9: General study characteristics of the included studies investigating the association between dairy intake and risk of T2D.

Author	Year	Country	Cohort name	Age at entry	Sex	Sample size	Total cases	Dietary assessment	Outcome assessment	Type of fish	Adjustment factors	Follow up years	Results (high vs. low intake category) Risk ratio (RR)
Djousse	2011	USA	WHS	≥45	Women	36328	2370	SFFQ	selfreported	fish	Age, Body Mass Index, energy intake, smoking, alcohol, menopausal status, coffee, cereal fiber, red meat, a-linolenic and linoleic acid, magnesium, trans fat, saturated fat, family history of diabetes, physical activity, glycemic index	12.4	1.49 (1.30, 1.71)
Ericson	2015	Sweden	MDC	45-74	Both	26930	2860	7 day menu book & SFFQ & interview	Registries or follow up examination	fish& shellfish, high fat	Age, Study area, Body Mass Index, energy intake, smoking, alcohol, coffee, fiber, calcium, magnesium, vegetable, fruit, meat, rice, family history of diabetes, Hypertension,	14	High-fat: 1.05 (0.94, 1.18)<br><br>Low-fat: 0.97 (0.86, 1.09)

											physical activity		
Kaushik	2009	USA	NHS II	26-46	Wo men	91669	2728	SFFQ	self-reported/extra questionnaire/Natio nal Diabetes Data group criteria	fish	Age, energy intake, Body Mass Index, smoking, alcohol, family history of diabetes, physical activity, saturated, trans fat, linolenic acid, linoleic acid, caffeine, cereal fiber, glycemic index, hormone replacement therapy, contraceptive use	14-18	1.16 (0.96, 1.40)
Kaushik	2009	USA	NHS	30-55	Wo men	61031	4159	SFFQ	self-reported/extra questionnaire/Natio nal Diabetes Data group criteria	fish	Age, energy intake, Body Mass Index, smoking, alcohol, family history of diabetes, physical activity, saturated, trans fat, linolenic acid, linoleic acid, caffeine, cereal fiber, glycemic index, menopausal status, postmenopausal hormone use	14-18	1.29 (1.05, 1.58)
Kaushik	2009	USA	HPFS	39-78	Men	42504	2493	SFFQ	self-reported/extra questionnaire/Natio nal Diabetes Data	fish	Age, energy intake, Body Mass Index, smoking,	14-18	1.32 (0.99, 1.76)

									group criteria		alcohol, family history of diabetes, physical activity, saturated, trans fat, linolenic acid, linoleic acid, caffeine, cereal fiber, glycemic index		
Krishnan	2010	USA	BWHS	30-69	Wo men	44072	2737	SFFQ	self reported	fried fish	Age, time period, education, TV watching, energy intake, Body Mass Index, smoking, alcohol, coffee, cereal fiber, sugar sweetened cola, calcium, vitamin D, family history of diabetes, physical activity, glycemic index	10	0.89 (0.64, 1.24)
Lacoppidan	2015	Denmark	DCH	50-64	Wo men	55060	7366	SFFQ	registry	fish	Age, Study area, Body Mass Index, energy intake, smoking, alcohol, coffee, fiber, calcium, magnesium, vegetable, fruit, meat, rice, family history of diabetes,	15.3	♀: 0.98 (0.91, 1.06) ♂: 1.02 (0.95, 1.08)

											Hypertension, physical activity		
Montonen	2005	Finland	Finnish Mobile Clinic Health Examination Survey	40-69	Both	4304	383	Dietary history interview	registration to the Social Insurance Institution	Unprocessed; salted or smoked	Age, Sex, energy intake, Body Mass Index, smoking, family history of diabetes, Geographic area	23	0.92 (0.77, 1.10)
Nanri	2011	Japan	JPHC	45-75	Men	52680	971	SFFQ	self reported	fish& seafood	Age, Study area, Body Mass Index, energy intake, smoking, alcohol, coffee, fiber, Ca, Mg, vegetable, fruit, meat, rice, family history of diabetes, Hypertension, physical activity	5	♂: 0.73 (0.54, 0.99) ♀: 1.01 (0.69
Patel	2012	Europe	EPIC-InterAct Study	50,7	Both	24813	10740	Quantitative questionnaire or SFFQ	self reported or care registers or medication use, mortality data	fish& shellfish	Age, Study area, Body Mass Index, energy intake, smoking, alcohol, coffee, fiber, Ca, Mg, vegetable, fruit, meat, rice, family history of diabetes, Hypertension, physical activity	NA	0.99 (0.86, 1.14)
Rylander	2014	Norway	NOWAC	30-70	Women	33740	479	FFQ	Self reported	Fish	Age, BMI, smoking, physical activity, hypertension	NA	0.66 (0.36, 1.21)

Vang	2008	USA	AMS & AHS	45-88	Both	8401	543	FFQ	self reported	fish	Age, Sex	17	1.12 (0.88, 1.43)
van Woudenbergh	2009	Netherlands	Rotterdam study	≥55	Both	4472	463	SFFQ	Health authorities	fish	Age, Sex, education, energy intake, smoking, alcohol, saturated, trans fat , fiber	15	1.32 (1.02, 1.71)
Villegas	2011	China	SWHS	40-70	Women	64193	2262	FFQ	self reported	fish& shellfish	Age, Body Mass Index, WHR, energy intake, smoking, alcohol, income, education, occupation, family history of diabetes, hypertension, physical activity, dietary pattern	8.9	0.86 (0.76, 0.79)
Villegas	2011	China	SMHS	40-74	Men	51936	833	FFQ	self reported	fish& shellfish	Age, Body Mass Index, WHR, energy intake, smoking, alcohol, income, education, occupation, family history of diabetes, hypertension, physical activity, dietary pattern	4.1	0.92 (0.73, 1.16)
Wallin	2015	Sweden	The Cohort of Swedish Men	45-79	Men	35583	3624	FFQ	registry	total fish	Age, Study area, Body Mass Index, energy intake, smoking, alcohol,	15	0.79 (0.60, 1.04)

											coffee, fiber, Ca, Mg, vegetable, fruit, meat, rice, family history of diabetes, Hypertension, physical activity		

Supplementary Table S10: General study characteristics of the included studies investigating the association between fish intake and risk of T2D.

**Author**	**Year**	**Country**	**Cohort name**	**Age at entry**	**Sex**	**Sample size**	**Total cases**	**Dietary assessment**	**Outcome assessment**	**Type of red meat**	**Adjustment factors**	**Follow up years**	**Results (high vs. low intake category)**
EPIC-Interact	2013	Europe	EPIC-InterAct	20-80	Both	16835	11559	Country specific dietary questionnaire	Self-reported/medication/ registers linkage/hospital records	red meat	sex, Body Mass Index, energy intake, smoking status, alcohol consumption, physical activity, educational level	11.7	1.20 (1.07, 1.35
Ericson	2015	Sweden	MDC	45-74	Both	26930	2860	7 day menu book & SFFQ & interview	Registries or follow up examination	red meat low-fat non processed	Age, Sex, season, method version, energy intake, Body Mass Index, education, smoking, alcohol, physical activity	14	Low-fat: 1.24 (1.1, 1.39)<br><br>High-fat: 1.01 (0.9, 1.14)
Fretts	2012	USA	SHFS		Both	2001	243	SFFQ	Medication or fasting plasma glucose concentration	unprocessed red meat	age, sex, site, energy intake, Body Mass Index, education, smoking, alcohol, family history of diabetes, physical activity,<br>fiber from grains and glycemic load	5	0.88 (0.57, 1.36)
Kurotani	2013	Japan	JPHC	45-75	Both	63849	1160	FFQ	self reported	red meat	Age, public health center area, Body Mass Index, smoking status, alcohol consumption, total physical activity,	5	♂:<br>1.58 (1.14, 2.19)<br><br>♀:<br>0.99 (0.67, 1.46)

											the history of hypertension, coffee consumption, the family history of diabetes, magnesium intake, calcium intake, rice intake, fish intake, vegetable intake, soft drink consumption energy intake, saturated fat		
Lee	2004	USA	IWHS	55-69	Wo men	35698	NA	SFFQ	self reported	red meat non drinkers	Age, energy intake, waist-hip ratio, Body Mass Index, physical activity, Cigarette, smoking, Alcohol, Education, Marital status, Residential area, Hormone replacement therapy, Animal fat, Vegetable fat, Cereal fiber, magnesium	11	Non-drinker: 1.11 (0.86, 1.42)<br><br>1-14g/d: 1.19 (0.84, 1.67)<br><br>≥15g/d 4.62 (1.5, 14.21)
Männistö	2010	Finland	ATBC	50-69	Men	25943	1098	picture FFQ	registration to the Social Insurance Institution	red meat	Age, energy intake, Body Mass Index, smoking, alcohol, physical activity, blood pressure, total	12	1.22 (0.97, 1.53)

											cholesterol, HDL-C, fruit, vegetable, rye, milk and coffee consumption		
Mari-Sanchis	2016	Spain	SUN	20-90	Both	18527	146	validated semi-quantitative FFQ	diagnosis of diabetes diagnosed by a doctor, patients were asked to confirm diagnosis with additional confirmation questionnaires and their medical records	Red meat	age, sex, physical activity, energy intake, Body Mass Index, family history of diabetes, , hypercholesterolemia, hypertension, dietary fiber intake, sugar sweetened beverages, smoking status, caffeine, glycemic index, adherence to Mediterranean dietary pattern, prevalent cardiovascular disease, prevalent cancer	8.7	0.95 (0.62, 1.46)
Montonen	2005	Finland	Finnish Mobile Clinic Health Examination Survey	40-69	Both	4304	383	Dietary history interview	registration to the Social Insurance Institution	red meat	Age, Sex, energy intake, Body Mass Index, smoking, family history of diabetes, Geographic area	23	0.99 (0.72, 1.36)
Pan	2011	USA	HPFS	40-75	Men	37083	2438	SFFQ	self-reported/extra questionnaire/medic	red meat	Age, energy intake, Body Mass	20	1.44 (1.23, 1.69)

									al records /National Diabetes Data group criteria or death reports		Index, Alcohol, physical activity, family history of diabetes, Smoking, race, history of hypertension and/or hypercholesterolemia, Menopausal status, Hormone use in women, dietary score		
Pan	2011	USA	NHS I	30-55	Women	79570	8253	SFFQ	self-reported/extra questionnaire/medical records /National Diabetes Data group criteria or death reports	red meat	Age, energy intake, Body Mass Index, Alcohol, physical activity, family history of diabetes, Smoking, race, history of hypertension and/or hypercholesterolemia, Menopausal status, Hormone use in women, dietary score	28	1.31 (1.21, 1.42)
Pan	2011	USA	NHS II	25-42	Women	87504	3068	SFFQ	self-reported/extra questionnaire/medical records /National Diabetes Data group criteria or death reports	red meat	Age, energy intake, Body Mass Index, Alcohol, physical activity, family history of diabetes, Smoking, race,	16	1.37 (1.19, 1.58)

											history of hypertension and/or hypercholesterolemia, Menopausal status, Hormone use in women, dietary score		
Song	2004	USA	WHS	≥45	Women	37309	1558	SFFQ	self-reported/blood sample/ extra questionnaire/ADA diagnostic criteria	red meat	Age, energy intake, Body Mass Index, Alcohol, physical activity, family history of diabetes, Smoking, fiber, magnesium, glycemic load, total fat	8.8	1.24 (1.00, 1.54)
Steinbrecher	2011	USA	MEC	45-75	Both	75512	8587	FFQ	self reported or with medication history or with the health plans	red meat	Age, energy intake, Body Mass Index, physical activity, Education, Ethnicity	14	♂ 1.43 (1.29, 1.59)<br>♀ 1.30 (1.17, 1.44)
van Woudenbergh	2012	Netherlands	Rotterdam Study	≥55	Both	4366	456	self administrated questionnaire and SFFQ	general practitioner registries	red meat	age, Body Mass Index, sex, smoking, diet prescription, and family history of diabetes, intake of energy, energy-adjusted carbohydrates, energy-adjusted	12.4	1.18 (0.88, 1.58)

											polyunsaturated fatty acids, energy-adjusted fiber, energy-adjusted milk, energy-adjusted cheese, soya, fish, alcohol, and tea, processed meat, poultry, CRP		
Villegas	2006	China	SWHS	40-70	Wo men	70609	1972	SFFQ	self reported	red meat	Age, WHR, energy intake, Body Mass Index, smoking, alcohol, physical activity, vegetable intake, income, education, occupation status, hypertension, chronic disease	4.6	0.94 (0.80, 1.10)

Supplementary Table S11: General study characteristics of the included studies investigating the association between red meat intake and risk of T2D.

Author	Year	Country	Cohort name	Age at entry	Sex	Sample size	Total cases	Dietary assessment	Outcome assessment	Type of processed meat	Adjustment factors	Follow up years	Results (high vs. low intake category) Risk ratio (RR)
EPIC-Interact	2013	Europe	EPIC-InterAct	20-80	Both	16835	11559	Country specific dietary questionnaire	Self-reported/medication/registers linkage/hospital records	processed meat	sex, Body Mass Index, energy intake, smoking status, alcohol consumption, physical activity, educational level	11.7	1.16 (1.04, 1.04, 1.29)
Ericson	2015	Sweden	MDC	45-74	Both	26930	2860	7 day menu book & SFFQ & interview	Registries or follow up examination	meat products low-fat processed	Age, Sex, season, method version, energy intake, Body Mass Index, education, smoking, alcohol, physical activity	14	Low fat: 1.16 (1.04, 1.30)<br><br>High-fat: 1.15 (1.01, 1.30)
Fretts	2012	USA	SHFS	35	Both	2001	243	SFFQ	Medication or fasting plasma glucose concentration	processed meat	age, sex, site, energy intake, Body Mass Index, education, smoking, alcohol, family history of diabetes, physical	5	1.35 (0.81, 2.25)

										activity, fiber from grains and glycemic load		
Kurotani	2013	Japan	JPHC	45-75	Both	63849	1160	FFQ	self reported	processed red meat	Age, public health center area, Body Mass Index, smoking status, alcohol consumption, total physical activity, the history of hypertension, coffee consumption, the family history of diabetes, magnesium intake, calcium intake, rice intake, fish intake, vegetable intake, soft drink consumption energy intake, saturated fat	5	♂: 1.15 (0.90, 1.47) ♀: 1.05 (0.79, 1.40)
Mari-Sanchis	2016	Spain	SUN	20-90	Both	18527	146	validated semi-quantitative FFQ	diagnosis of diabetes diagnosed by a doctor, patients	Processed meat	age, sex, physical activity, energy intake, Body	8.7	1.60 (0.78, 3.28)

									were asked to confirm diagnosis with additional confirmation questionnaires and their medical records		Mass Index, family history of diabetes, , hypercholesterolemia, hypertension, dietary fiber intake, sugar sweetened beverages, smoking status, caffeine, glycemic index, adherence to		
Männistö	2014	Finland	ATBC	50-69	Men	25943	1098	picture FFQ	registration to the Social Insurance Institution	Processed meat	Age, energy intake, Body Mass Index, smoking, alcohol, physical activity, BP, Serum cholesterol, Serum HDL, fruit, vegetable, rye, milk and coffee consumption	12	1.37 (1.11, 1.71)
Montonen	2005	Finland	Finnish Mobile Clinic Health Examination Survey	40-69	Both	4304	383	Dietary history interview	registration to the Social Insurance Institution	processed meat	Age, Sex, energy intake, Body Mass Index, smoking, family history of diabetes, Geographic	23	1.22 (0.89, 1.67)

											area		
Pan	2011	USA	HPFS	40-75	Men	37083	2438	SFFQ	self-reported/extra questionnaire/medical records /National Diabetes Data group criteria or death reports	processed red meat	Age, energy intake, Body Mass Index, Alcohol, physical activity, family history of diabetes, Smoking, race, history of hypertension and/or hypercholesterolemia, Menopausal status, Hormone use in women, dietary score	20	1.55 (1.33, 1.81)
Pan	2011	USA	NHS I	30-55	Women	79570	8253	SFFQ	self-reported/extra questionnaire/medical records /National Diabetes Data group criteria or death reports	processed red meat	Age, energy intake, Body Mass Index, Alcohol, physical activity, family history of diabetes, Smoking, race, history of hypertension and/or Hypercholesterolemia, Menopausal	28	1.30 (1.20, 1.41)

											status, Hormone use in women, dietary score		
Pan	2011	USA	NHS II	25-42	Wom en	87504	3068	SFFQ	self-reported/extra questionnaire/m edical records /National Diabetes Data group criteria or death reports	processed red meat	Age, energy intake, Body Mass Index, Alcohol, physical activity, family history of diabetes, Smoking, race, history of hypertension and/or hypercholestero lemia, Menopausal status, Hormone use in women, dietary score	16	1.21 (1.07, 1.37)
Song	2004	USA	WHS	≥45	Wom en	37309	1558	SFFQ	self-reported/blood sample/ extra questionnaire/A DA diagnostic criteria	processed meat	Age, energy intake, Body Mass Index, Alcohol, physical activity, family history of diabetes, Smoking, fiber, Mg, GL, total fat	8.8	1.19 (1.00, 1.42)
Steinbreche	2011	USA	MEC	45-75	Both	75512	8587	FFQ	self reported or	processed	Age, energy	14	♂:

r									with medication history or with the health plans	red meat	intake, Body Mass Index, physical activity, Education, Ethnicity		1.44 (1.33, 1.56) ♀ 1.34 (1.24, 1.45)
van Woudenbergh	2012	Netherlands	Rotterdam Study	≥55	Both	4366	456	self administrated questionnaire and SFFQ	general practitioner registries	processed meat	age, Body Mass Index, sex, smoking, diet prescription, and family history of diabetes, intake of energy, energy-adjusted carbohydrates, energy-adjusted polyunsaturated fatty acids, energy-adjusted fiber, energy-adjusted milk, energy-adjusted cheese, soya, fish, alcohol, and tea, red meat, poultry, CRP	12.4	1.73 (1.16 2.58)
Villegas	2006	China	SWHS	40-70	Women	70609	1972	SFFQ	self reported	processed meat	Age, WHR, energy intake, Body Mass Index, smoking, alcohol, physical activity,	4.6	1.18 (0.99, 1.41)

											vegetable intake, income, education, occupation status, hypertension, chronic disease		

Supplementary Table S12: General study characteristics of the included studies investigating the association between processed meat intake and risk of T2D.

Author	Year	Country	Cohort name	Age at entry	Sex	Sample size	Total cases	Dietary assessment	Outcome assessment	Type of SSB	Adjustment factors	Follow up years	Results (high vs. low intake category) Risk ratio (RR)
Bhupathiraju	2013	USA	NHS	38-63	Women	74749	7370	SFFQ	self-reported/extra questionnaire/ National Diabetes Data group and ADA criteria	Caffeinated carbonated SSB	Age, time interval, AHEI, adherence to low caloric diet, reported weight gain, energy intake, Body Mass Index, Alcohol, physical activity, family history of diabetes, Smoking, history of hypertension and/or hypercholesterolemia, Postmenopausal hormone use	24	Caffeinated carbonated SSB: 1.29 (1.14, 1.47)<br><br>Caffeine-free carbonated SSB: 1.20 (1.01, 1.42)
Bhupathiraju	2013	USA	HPFS	40-75	Men	39059	2865	SFFQ	self-reported/extra questionnaire/ National Diabetes Data group and ADA criteria	Caffeinated carbonated SSB	Age, time interval, AHEI, adherence to low caloric diet, reported weight gain, energy intake, Body Mass Index, Alcohol, physical activity, family history of diabetes, Smoking, history of hypertension and/or hypercholesterolem	22	Caffeinated carbonated SSB: 1.33 (1.10, 1.60)<br><br>Caffeine-free carbonated SSB: 1.37 (1.08, 1.74)

											ia		
EPIC-Interact	2013	Europe	EPIC-InterAct	20-80	Both	16154	12403	country specific dietary questionnaires	self reported or care registers or medication use, mortality data	SSB	Sex, Body Mass Index, energy intake, education, smoking, alcohol, physical activity, artificially sweetened beverages, juice	NA	1.29 (1.02 1.63)
Eshak	2013	Japan	JPHC	40-59	Both	27585	824	FFQ	self-reported/extra questionnaire/ National Diabetes Data group criteria	SSB	Age, Body Mass Index, family history of diabetes mellitus, education, occupation, alcohol intake, history of hypertension, leisure-time physical activity, consumption of coffee, consumption of green tea, energy adjusted intakes of dietary magnesium, calcium, vitamin D, rice and total dietary fiber, and total energy intake	10	♂: 0.98 (0.68, 1.41)<br><br>♀: 1.79 (1.11, 2.89)
Montonen	2007	Finland	Finnish Mobile Clinic Health Examination Survey	40-60	Both	2360	91	dietary history interview	registration to the Social Insurance Institution	SSB	Age, Sex, energy intake, Body Mass Index, smoking, family history of diabetes, Geographic area, physical activity,	12	1.60 (0.93, 2.75)

											prudent dietary pattern score, conservative pattern score, serum cholesterol, blood pressure, history of Infraction, Angina pectoris or cardiac failure		
Odegaard	2010	China	The Singapore Chinese Health Study	45-74	Both	43580	2273	SFFQ	self reported	SSB	Age, Sex, dialect, year of interview, education, energy intake, Body Mass Index, smoking, alcohol, saturated fat, dietary fiber, dairy, juice, coffee, physical activity	5.7	1.34 (1.17, 1.53)
Palmer	2008	USA	BWHS	21-69	Women	43960	2713	SFFQ	self reported	SSB	Age, family history of diabetes, education, smoking, red meat, processed meat, cereal fiber, GI, coffee, physical activity	10	1.24 (1.06, 1.45)
Paynter	2006	USA	ARIC	45-64	Both	12204	1437	SFFQ	self reported	SSB	Age, race, family history of diabetes, Hypertension, education, energy intake, Body Mass Index, WHR, smoking, alcohol, dietary fiber,	12	♂ 1.02 (0.76, 1.37)<br>♀ 1.07 (0.79, 1.45)

											physical activity, using diagnosis/medication for outcome classification		
Sakurai	2014	Japan	NA	35-55	Men	2037	170	diet history questionnaire	HbA1c measurement/ ADA criteria	SSB	Age, family history of diabetes, Hypertension, Dyslipidemia, diet treatment for chronic disease, energy intake, Body Mass Index, smoking, alcohol, dietary fiber, physical activity, diet soda, fruit juice, vegetable juice, coffee	5.5	1.34 (0.72, 2.49)
Schulze	2004	USA	NHS II	26-46	Women	91249	741	SFFQ	self-reported/extra questionnaire/ National Diabetes Data group criteria	SSB	Age, smoking, alcohol, family history of diabetes, physical activity, polyunsaturated to saturated ratio, trans fat, magnesium, cereal fiber, diet soft drinks, fruit juice, fruit punch, postmenopausal hormone use, contraceptive use	8	1.83 (1.42, 2.36)

Supplementary Table S13: General study characteristics of the included studies investigating the association between sugar sweetened beverages intake and risk of T2D.

Dietary factor	No of studies	RR	95% CI	I² (%)<br>95% CI
Whole grains				
High vs. low	13	0.77	0.71, 0.84	86 (78, 91)
Dose-response	12	0.87	0.82, 0.93	91 (86, 94)
Low risk of bias	8	0.85	0.79, 0.92	92
Gender				
Women	5	0.85	0.77, 0.93	92
Men	2	0.78	0.70, 0.88	58
Men and women	5	0.95	0.91, 0.99	48
Age				
<50	3	0.78	0.68, 0.91	87
≥50	9	0.90	0.84, 0.96	91
Follow-up				
<10 years	6	0.92	0.88, 0.98	71
≥10 years	6	0.82	0.74, 0.91	93
Geographic location				
Europe	4	0.94	0.90, 0.98	53
America	7	0.83	0.76, 0.90	89
Asia & Australia	1	1.00	0.92, 1.08	NA
Number of cases				
<1000	3	0.92	0.84, 1.02	73
≥1000	9	0.86	0.80, 0.92	92
Dietary assessment method				
FFQ	10	0.85	0.79, 0.92	90
24h-recall/Food records	2	0.96	0.92, 0.99	0
Outcome assessment				

Self-reported	7	0.83	0.76, 0.90	89
Diagnosed by physician	3	0.93	0.84, 1.02	73
Registry	2	0.96	0.92, 0.99	0

Supplementary Table S14. Dose-response meta-analysis for each 30 g/d increase in whole grains and risk of type 2 diabetes, stratified by low risk of bias studies, high vs. low intake, gender, follow-up, geographic location, number of cases, dietary assessment method, and outcome assessment

Dietary factor	No of studies	RR	95% CI	I² (%)<br>95% CI
Refined grains				
High vs. low	15	1.01	0.92, 1.10	54 (16, 74)
Dose-response	14	1.01	0.99, 1.03	59 (23, 78)
Low risk of bias	8	1.02	1.00, 1.04	32
Gender				
Women	5	1.01	0.98, 1.04	68
Men	3	1.03	1.00, 1.07	12
Men and women	6	1.01	0.98, 1.04	74
Age				
<50	4	1.04	1.01, 1.06	0
≥50	10	1.00	0.98, 1.02	60
Follow-up				
<10 years	6	1.00	0.97, 1.02	70
≥10 years	8	1.02	1.00, 1.04	37
Geographic location				
Europe	2	0.98	0.95, 1.02	0
America	8	1.02	0.99, 1.04	67
Asia & Australia	4	1.01	0.98, 1.04	63
Number of cases				
<1000	4	1.01	0.96, 1.05	66
≥1000	10	1.01	0.99, 1.03	58
Dietary assessment method				
FFQ	12	1.01	1.00, 1.03	61
24h-recall/Food records	2	0.98	0.95, 1.02	0
Outcome assessment				

Self-reported	9	1.01	0.99, 1.03	63
Diagnosed by physician	3	1.03	0.96, 1.10	71
Registry	2	0.98	0.95, 1.02	0

Supplementary Table S15. Dose-response meta-analysis for each daily 30 g/d increase in refined grains and risk of type 2 diabetes, stratified by low risk of bias studies, high vs. low intake, gender, follow-up, geographic location, number of cases, dietary assessment method, and outcome assessment

Dietary factor	No of studies	RR	95% CI	I² (%)<br>95% CI
Vegetables				
High vs. low	13	0.95	0.89, 1.01	59 (24, 78)
Dose-response	11	0.98	0.96, 1.00	62 (29, 81)
Low risk of bias	5	0.99	0.97, 1.02	42
Gender				
Women	4	0.99	0.95, 1.03	82
Men	1	0.90	0.78, 1.05	NA
Men and women	6	0.98	0.97, 0.99	0
Age				
≥50	11	0.98	0.96, 1.00	62
Follow-up				
<10 years	5	0.98	0.94, 1.01	70
≥10 years	6	0.99	0.96, 1.02	62
Geographic location				
Europe	4	0.97	0.89, 1.05	44
America	4	1.00	0.98, 1.02	71
Asia & Australia	3	0.95	0.91, 0.99	44
Number of cases				
<1000	4	0.96	0.93, 1.00	0
≥1000	7	0.99	0.97, 1.01	74
Dietary assessment method				
FFQ	7	0.99	0.96, 1.01	71
24h-recall/Food records	4	0.97	0.89, 1.05	44
Outcome assessment				
Self-reported	7	0.99	0.96, 1.01	75

Diagnosed by physician	2	0.98	0.95, 1.01	0
Registry	2	0.89	0.80, 1.00	0

Supplementary Table S16. Dose-response meta-analysis for each 100 g/d increase in vegetables and risk of type 2 diabetes, stratified by low risk of bias studies, high vs. low intake, gender, follow-up, geographic location, number of cases, dietary assessment method, and outcome assessment

Dietary factor	No of studies	RR	95% CI	I² (%)<br>95% CI
Fruits				
High vs. low	15	0.96	0.93, 1.00	29 (0, 62)
Dose-reponse	13	0.98	0.97, 1.00	21 (0, 58)
Low risk of bias	7	0.96	0.95, 0.98	0
Gender				
Women	5	0.98	0.96, 1.01	50
Men	2	0.97	0.93, 1.02	0
Men and women	6	0.98	0,97, 1.00	23
Age				
<50	2	0.96	0.93, 0.98	0
≥50	11	0.99	0.97, 1.00	1
Follow-up				
<10 years	5	1.00	0.98, 1.02	0
≥10 years	8	0.97	0.96, 0.99	35
Geographic location				
Europe	4	0.97	0.92, 1.01	31
America	6	0.98	0.96, 1.00	40
Asia & Australia	3	0.99	0.97, 1.02	0
Number of cases				
<1000	4	0.97	0.92, 1.02	35
≥1000	9	0.98	0.97, 1.00	22
Dietary assessment method				
FFQ	9	0.99	0.97, 1.00	16
24h-recall/Food records	4	0.97	0.92, 1.01	31
Outcome assessment				

Self-reported	9	0.99	0.97, 1.00	15
Diagnosed by physician	2	0.97	0.94, 1.00	0
Registry	2	0.92	0.80, 1.06	0

Supplementary Table S17. Dose-response meta-analysis for each 100 g/d increase in fruits and risk of type 2 diabetes, stratified by low risk of bias studies, high vs. low intake, gender, follow-up, geographic location, number of cases, dietary assessment method, and outcome assessment

Dietary factor	No of studies	RR	95% CI	I^2 (%) 95% CI
Nuts				
High vs. low	8	0.95	0.85, 1.05	67 (30, 84)
Dose-reponse	7	0.89	0.71, 1.12	77 (52, 89)
Low risk of bias	4	0.93	0.81, 1.05	38
Gender				
Women	4	1.06	0.81, 1.38	76
Men	1	0.82	0.65, 1.04	NA
Men and women	2	0.54	0.24, 1.22	71
Age				
<50	3	0.45	0.15, 1.34	84
≥50	4	0.98	0.76, 1.25	78
Follow-up				
<10 years	3	0.42	0.18, 0.97	74
≥10 years	4	1.04	0.86, 1.25	70
Geographic location				
Europe	1	0.75	0.55, 1.01	NA
America	4	1.04	0.86, 1.25	70
Asia & Australia	2	0.27	0.13, 0.56	0
Number of cases				
<1000	2	0.54	0.24, 1.22	71
≥1000	5	1.00	0.80, 1.25	75
Dietary assessment method				
FFQ	7	0.89	0.71, 1.12	77
Outcome assessment				
Self-reported	5	1.00	0.80, 1.25	75

Diagnosed by physician	2	0.54	0.24, 1.22	71

Supplementary Table S18. Dose-response meta-analysis for each 28 g/d increase in nuts and risk of type 2 diabetes, stratified by low risk of bias studies, high vs. low intake, gender, follow-up, geographic location, number of cases, dietary assessment method, and outcome assessment

Dietary factor	No of studies	RR	95% CI	I^2 (%)<br>95% CI
Legumes				
High vs. low	12	0.96	0.87, 1.05	85 (75, 91)
Dose-response	12	1.00	0.92, 1.09	87 (79, 92)
Low risk of bias	8	1.08	0.97, 1.22	83
Gender				
Women	5	0.87	0.65, 1.15	91
Men	1	0.42	0.04, 4.01	NA
Men and women	6	1.05	0.96, 1.14	86
Age				
<50	2	0.65	0.57, 0.74	0
≥50	10	1.05	0.98, 1.12	78
Follow-up				
<10 years	6	0.93	0.85, 1.01	89
≥10 years	6	1.19	0.91, 1.54	78
Geographic location				
Europe	2	1.01	0.98, 1.03	0
America	6	1.17	0.90, 1.51	82
Asia & Australia	4	0.89	0.75, 1.14	91
Number of cases				
<1000	3	1.01	0.98, 1.03	0
≥1000	9	1.01	0.88, 1.16	90
Dietary assessment method				
FFQ	11	1.00	0.91, 1.10	88
24h-recall/Food records	1	1.04	0.90, 1.20	NA
Outcome assessment				

Self-reported	8	0.92	0.80, 1.05	86
Diagnosed by physician	2	1.01	0.98, 1.03	0
Registry	2	1.41	0.76, 2.60	95

Supplementary Table S19. Dose-response meta-analysis for each 50 g/d increase in legumes and risk of type 2 diabetes, stratified by low risk of bias studies, high vs. low intake, gender, follow-up, geographic location, number of cases, dietary assessment method, and outcome assessment

Dietary factor	No of studies	RR	95% CI	I^2 (%)<br>95% CI
Eggs				
High vs. low	13	1.08	0.95, 1.22	69 (45, 83)
Dose-response	13	1.08	0.95, 1.22	77 (61, 86)
Low risk of bias	8	1.04	0.87, 1.25	85
Gender				
Women	2	1.29	0.96, 1.72	82
Men	3	0.98	0.65, 1.47	94
Men and women	8	1.04	0.93, 1.17	29
Age				
<50	1	0.54	0.21, 1.40	NA
≥50	12	1.09	0.96, 1.24	78
Follow-up				
<10 years	3	0.96	0.84, 1.08	0
≥10 years	10	1.12	0.97, 1.30	79
Geographic location				
Europe	6	0.96	0.82, 1.12	77
America	5	1.43	1.28, 1.61	0
Asia & Australia	1	0.96	0.82, 1.12	NA
Number of cases				
<1000	5	0.91	0.65, 1.27	80
≥1000	8	1.17	1.05, 1.31	70
Dietary assessment method				
FFQ	10	1.17	1.03, 1.32	65
24h-recall/Food records	3	0.83	0.53, 1.30	91
Outcome assessment				

Self-reported	5	1.29	1.03, 1.62	77
Diagnosed by physician	4	1.06	0.93, 1.20	5
Registry	4	0.92	0.72, 1.18	87

Supplementary Table S20. Dose-response meta-analysis for each 50 g/d increase in eggs and risk of type 2 diabetes, stratified by low risk of bias studies, high vs. low intake, gender, follow-up, geographic location, number of cases, dietary assessment method, and outcome assessment

Dietary factor	No of studies	RR	95% CI	I² (%)<br>95% CI
Dairy				
High vs. low	21	0.91	0.85, 0.97	63 (41, 77)
Dose-reponse	21	0.97	0.94, 0.99	74 (60, 83)
Low risk of bias	12	0.97	0.95, 1.00	82
Low fat	14	0.97	0.94, 1.00	71
High fat	14	1.00	0.96, 1.04	69
Gender				
Women	6	0.93	0.88, 0.99	91
Men	2	0.99	0.96, 1.02	0
Men and women	13	0.98	0.95, 1.00	34
Age				
<50	5	0.99	0.96, 1.02	47
≥50	16	0.95	0.93, 0.98	77
Follow-up				
<10 years	10	0.89	0.83, 0.96	79
≥10 years	11	0.99	0.98, 1.01	40
Geographic location				
Europe	8	0.98	0.95, 1.01	36
America	7	0.98	0.96, 1.00	58
Asia & Australia	6	0.84	0.71, 1.01	85
Number of cases				
<1000	11	0.90	0.84, 0.96	79
≥1000	10	0.99	0.98, 1.01	45
Dietary assessment method				
FFQ	18	0.95	0.92, 0.98	78

24h-recall/Food records	3	0.99	0.98, 1.00	0
Outcome assessment				
Self-reported	13	0.96	0.93, 1.00	81
Diagnosed by physician	5	0.91	0.83, 0.99	59
Registry	3	0.99	0.98, 1.00	0

Supplementary Table S21. Dose-response meta-analysis for each 200 g/d increase in dairy and risk of type 2 diabetes, stratified by low risk of bias studies, high vs. low-fat dairy products, high vs. low intake, gender, follow-up, geographic location, number of cases, dietary assessment method, and outcome assessment

Dietary factor	No of studies	RR	95% CI	I² (%)<br>95% CI
Fish				
High vs. low	16	1.04	0.95, 1.13	76 (62, 86)
Dose-response	15	1.09	0.93, 1.28	84 (76, 90)
Low risk of bias	9	1.20	0.99, 1.46	83
Gender				
Women	6	1.22	0.86, 1.73	93
Men	2	1.07	0.79, 1.46	78
Men and women	7	0.97	0.86, 1.09	85
Age				
<50	4	0.99	0.65, 1.52	84
≥50	11	1.11	0.93, 1.33	84
Follow-up				
<10 years	4	0.87	0.79, 0.94	0
≥10 years	11	1.20	0.99, 1.45	81
Geographic location				
Europe	6	0.94	0.78, 1.14	48
America	6	1.44	1.19, 1.74	66
Asia & Australia	3	0.87	0.80, 0.95	0
Number of cases				
<1000	6	0.90	0.69, 1.18	35
≥1000	9	1.14	0.93, 1.39	89
Dietary assessment method				
FFQ	12	1.12	0.91, 1.39	87
24h-recall/Food records	3	0.98	0.88, 1.10	0
Outcome assessment				

Self-reported	10	1.15	0.93, 1.42	88
Diagnosed by physician	1	1.00	0.82, 1.22	5
Registry	4	0.96	0.70, 1.32	64

Supplementary Table S22. Dose-response meta-analysis for each 100 g/d increase in fish and risk of type 2 diabetes, stratified by low risk of bias studies, high vs. low intake, gender, follow-up, geographic location, number of cases, dietary assessment method, and outcome assessment

Dietary factor	No of studies	RR	95% CI	I² (%)<br>95% CI
Red meat				
High vs. low	15	1.21	1.13, 1.30	65 (41, 80)
Dose-response	14	1.17	1.08, 1.26	83 (72, 89)
Low risk of bias	10	1.22	1.13, 1.32	83
Gender				
Women	4	1.11	1.01, 1.22	78
Men	2	1.21	1.13, 1.29	0
Men and women	8	1.18	1.01, 1.38	82
Age				
<50	4	1.14	1.06, 1.22	46
≥50	10	1.19	1.06, 1.33	84
Follow-up				
<10 years	5	1.01	0.82, 1.25	73
≥10 years	9	1.23	1.13, 1.33	85
Geographic location				
Europe	6	1.15	1.05, 1.27	38
America	6	1.23	1.11, 1.36	88
Asia & Australia	2	1.03	0.63, 1.68	89
Number of cases				
<1000	4	0.98	0.85, 1.13	0
≥1000	10	1.21	1.21, 1.32	86
Dietary assessment method				
FFQ	11	1.17	1.06, 1.28	86
24h-recall/Food records	3	1.19	1.04, 1.35	52
Outcome assessment				

Self-reported	7	1.20	1.08, 1.34	91
Diagnosed by physician	3	1.04	0.78, 1.38	70
Registry	4	1.16	1.04, 1.29	15

Supplementary Table S23. Dose-response meta-analysis for each 100 g/d increase in red meat and risk of type 2 diabetes, stratified by low risk of bias studies, high vs. low intake, gender, follow-up, geographic location, number of cases, dietary assessment method, and outcome assessment

Dietary factor	No of studies	RR	95% CI	I^2 (%)<br>95% CI
Processed meat				
High vs. low	14	1.27	1.20, 1.35	55 (18, 75)
Dose-response	14	1.37	1.22, 1.55	87 (81, 92)
Low risk of bias	10	1.39	1.21, 1.60	92
Gender				
Women	4	1.49	1.36, 1.63	0
Men	2	1.41	0.88, 2.26	96
Men and women	8	1.33	1.11, 1.59	90
Age				
<50	4	1.43	1.27, 1.61	24
≥50	10	1.36	1.18, 1.57	90
Follow-up				
<10 years	5	1.30	1.13, 1.50	0
≥10 years	9	1.39	1.20, 1.61	92
Geographic location				
Europe	6	1.13	1.09, 1.18	0
America	6	1.65	1.47, 1.85	54
Asia & Australia	2	1.35	1.01, 1.79	0
Number of cases				
<1000	4	1.32	1.13, 1.54	0
≥1000	10	1.38	1.20, 1.58	91
Dietary assessment method				
FFQ	11	1.47	1.25, 1.72	87
24h-recall/Food records	3	1.13	1.07, 1.19	0
Outcome assessment				

Self-reported	7	1.62	1.45, 1.81	53
Diagnosed by physician	3	1.11	1.04, 1.18	0
Registry	4	1.15	1.09, 1.22	2

Supplementary Table S24. Dose-response meta-analysis for each 50 g/d increase in processed meat and risk of type 2 diabetes, stratified by low risk of bias studies, high vs. low intake, gender, follow-up, geographic location, number of cases, dietary assessment method, and outcome assessment

Dietary factor	No of studies	RR	95% CI	I² (%)<br>95% CI
Sugar sweetened beverages				
High vs. low	10	1.30	1.20, 1.40	34 (0, 69)
Dose-response	10	1.21	1.12, 1.31	78 (59, 88)
Low risk of bias	8	1.24	1.14, 1.35	79
Gender				
Women	3	1.22	1.08, 1.39	88
Men	2	1.25	1.14, 1.37	0
Men and women	5	1.22	1.04, 1.42	80
Age				
<50	4	1.26	1.00, 1.58	81
≥50	6	1.20	1.09, 1.32	79
Follow-up				
<10 years	3	1.53	1.35, 1.74	0
≥10 years	7	1.14	1.07, 1.21	65
Geographic location				
Europe	2	1.65	0.70, 3.90	80
America	5	1.18	1.08, 1.29	85
Asia & Australia	3	1.33	1.10, 1.61	34
Number of cases				
<1000	4	1.45	1.10, 1.92	63
≥1000	6	1.16	1.09, 1.24	76
Dietary assessment method				
FFQ	7	1.21	1.11, 1.32	83
24h-recall/Food records	3	1.35	0.95, 1.92	62

Outcome assessment				
Self-reported	7	1.21	1.11, 1.32	83
Diagnosed by physician	2	1.15	1.07, 1.25	0
Registry	1	2.81	1.30, 6.09	NA

Supplementary Table S25. Dose-response meta-analysis for each 250 ml/d increase in sugar sweetened beverages and risk of type 2 diabetes, stratified by low risk of bias studies, high vs. low intake, gender, follow-up, geographic location, number of cases, dietary assessment method, and outcome assessment

Dietary factor	Amount	No of studies	RR	95% CI	I^2 (%)	NutriGrade grading
Whole grains	30g	12	0.87	0.82, 0.93	91	High[1]
Refined grains	30g	14	1.01	0.99, 1.03	59	Moderate[2]
Vegetables	100g	11	0.98	0.96, 1.00	62	Moderate[2]
Fruits	100g	13	0.98	0.97, 1.00	21	Moderate[2]
Nuts	28g	7	0.89	0.71, 1.12	77	Low[3]
Legumes	50g	12	1.00	0.92, 1.09	87	Low[3]
Eggs	50g	13	1.08	0.95, 1.22	77	Moderate[2]
Dairy	200g	21	0.97	0.94, 0.99	74	Moderate[2]
Fish	100g	15	1.09	0.93, 1.28	84	Moderate[2]
Red meat	100g	14	1.17	1.08, 1.26	83	High[1]
Processed meat	50g	14	1.37	1.22, 1.55	88	High[1]
Sugar sweetened beverages	250ml	10	1.21	1.12, 1.31	78	High[1]

Supplementary Table S26: Linear dose-response meta-analysis including 12 dietary factors and the risk of T2D, and NutriGrade grading.

[1]There is high confidence for the effect estimate, and further research probably will not change the confidence in the effect estimate.

[2]There is moderate confidence for the effect estimate, further research could add evidence on the confidence and may change the effect estimate.

[3]There is low confidence for the effect estimate, further research will provide important evidence on the confidence and likely change the effect estimate.

Study or Subgroup	Weight	Risk Ratio IV, Random, 95% CI
Ericson 2013	8.4%	0.84 [0.72, 0.98]
Hodge 2004	4.6%	0.86 [0.63, 1.17]
Kochar 2007	7.6%	0.60 [0.50, 0.72]
Lacoppidan 2015	11.2%	0.90 [0.87, 0.93]
Meyer 2000	6.7%	0.93 [0.75, 1.15]
Montonen 2003	1.8%	0.65 [0.36, 1.17]
Parker 2013	7.2%	0.78 [0.64, 0.95]
Sun 2010 HPFS	9.0%	0.72 [0.63, 0.82]
Sun 2010 NHS	10.1%	0.70 [0.64, 0.77]
Sun 2010 NHS II	10.6%	0.73 [0.68, 0.78]
van Dam 2006	8.8%	0.69 [0.60, 0.79]
von Ruesten 2013	10.4%	0.92 [0.85, 1.00]
Wirström 2013	3.8%	0.59 [0.41, 0.85]
**Total (95% CI)**	**100.0%**	**0.77 [0.71, 0.84]**

Heterogeneity: Tau² = 0.02; Chi² = 83.35, df = 12 (P < 0.00001); I² = 86%
Test for overall effect: Z = 5.92 (P < 0.00001)

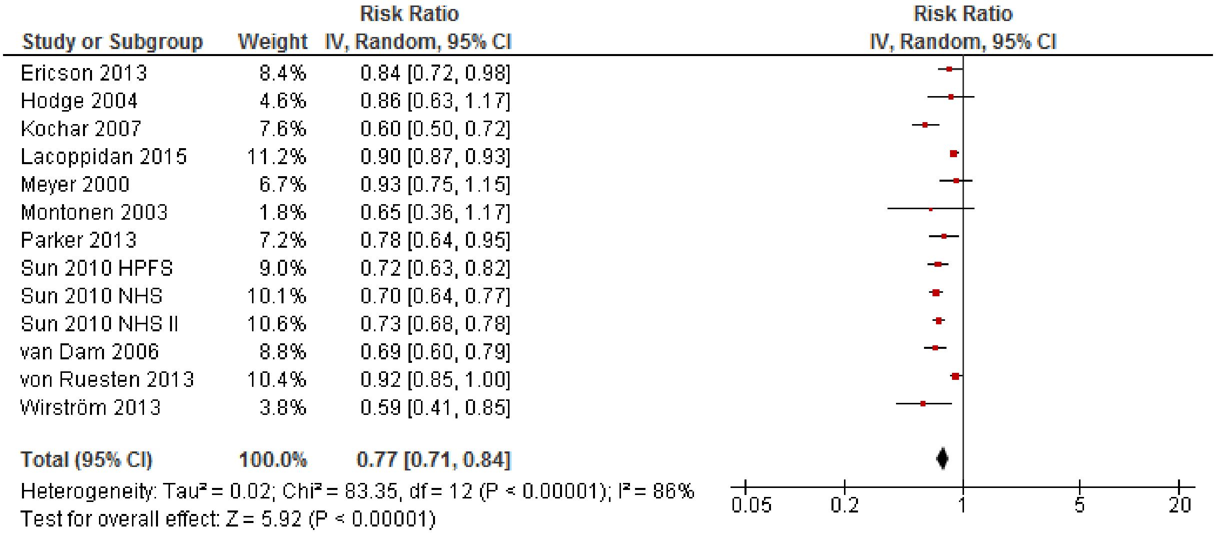

Supplementary Figure S1: Summary of relative risk of type 2 diabetes for high versus low whole grains intake. 95% CI, 95% confidence interval;

Study or Subgroup	Weight	Risk Ratio IV, Random, 95% CI
Ericson 2013	9.0%	0.96 [0.91, 1.01]
Hodge 2004	8.2%	1.00 [0.92, 1.08]
Kochar 2007	6.8%	0.73 [0.64, 0.83]
Meyer 2000	8.4%	0.97 [0.90, 1.05]
Montonen 2003	9.0%	0.95 [0.90, 1.00]
Parker 2013	8.6%	0.93 [0.87, 1.00]
Sun 2010 HPFS	8.3%	0.82 [0.76, 0.89]
Sun 2010 NHS	8.0%	0.70 [0.64, 0.77]
Sun 2010 NHS II	9.3%	0.78 [0.75, 0.81]
van Dam 2006	9.1%	0.86 [0.82, 0.91]
von Ruesten 2013	9.1%	0.95 [0.91, 1.00]
Wirström 2013	6.3%	0.79 [0.68, 0.91]
**Total (95% CI)**	**100.0%**	**0.87 [0.82, 0.93]**

Heterogeneity: Tau² = 0.01; Chi² = 117.33, df = 11 (P < 0.00001); I² = 91%
Test for overall effect: Z = 4.37 (P < 0.0001)

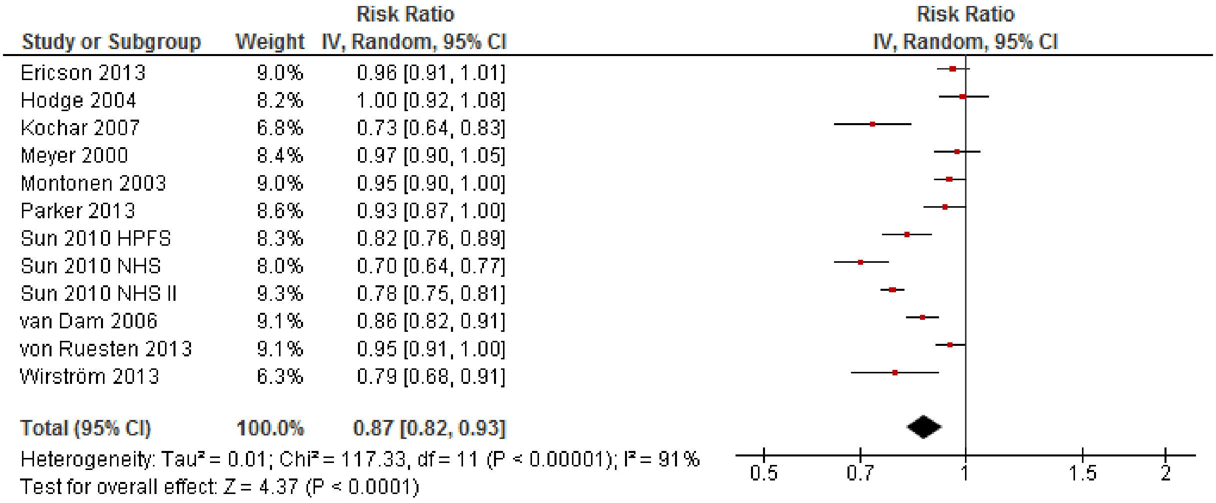

Supplementary Figure S2: Summary of relative risk of type 2 diabetes for each 30g/d increase in whole grains intake. 95% CI, 95% confidence interval;

Study or Subgroup	Weight	Risk Ratio IV, Random, 95% CI
Ericson 2013	9.9%	1.05 [0.90, 1.22]
Fung 2002	7.8%	1.08 [0.87, 1.34]
Golozar 2017 GCS	8.0%	0.90 [0.73, 1.11]
Golozar 2017 TLGS	1.7%	2.28 [1.19, 4.37]
Hodge 2004	7.7%	1.02 [0.82, 1.27]
Kochar 2007	6.4%	0.95 [0.73, 1.24]
Liu 2000	9.5%	1.11 [0.94, 1.31]
Meyer 2000	7.7%	0.87 [0.70, 1.08]
Montonen 2003	2.3%	0.62 [0.36, 1.07]
Nanri 2010	11.0%	1.02 [0.90, 1.16]
Parker 2013	7.3%	0.77 [0.61, 0.97]
Soriguer 2013 rice	1.0%	0.41 [0.17, 0.99]
Sun 2010 HPFS rice	6.0%	1.02 [0.77, 1.35]
Sun 2010 NHS II rice	6.8%	1.40 [1.09, 1.80]
Sun 2010 NHS rice	6.9%	1.11 [0.87, 1.42]
**Total (95% CI)**	**100.0%**	**1.01 [0.92, 1.10]**

Heterogeneity: Tau² = 0.02; Chi² = 30.52, df = 14 (P = 0.006); I² = 54%
Test for overall effect: Z = 0.18 (P = 0.86)

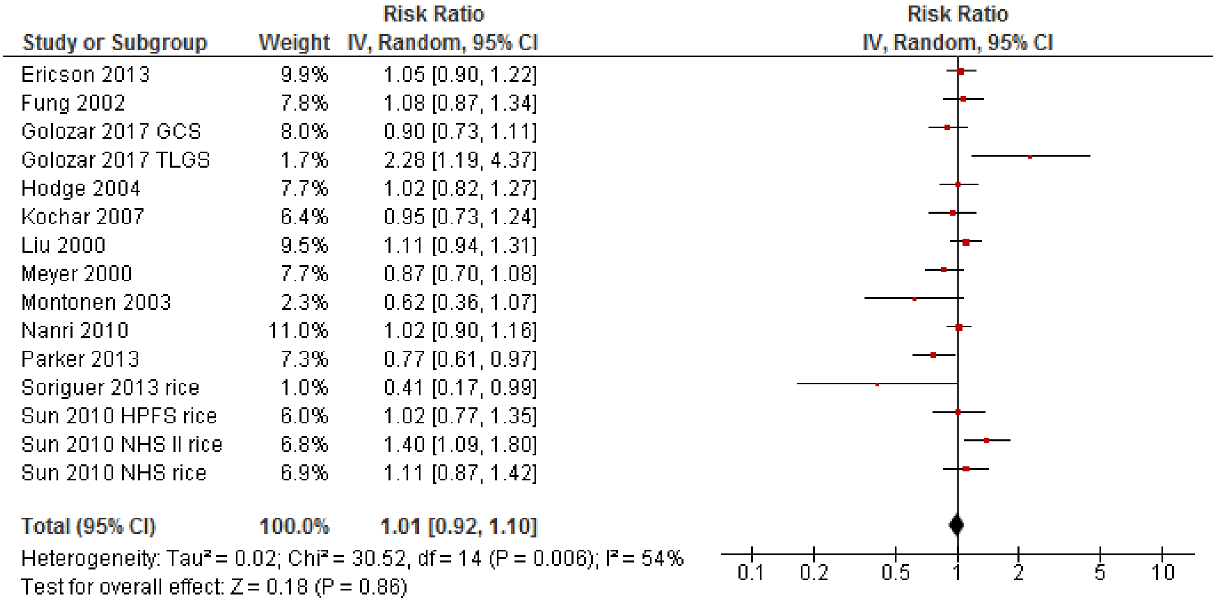

Supplementary Figure S3: Summary of relative risk of type 2 diabetes for high versus low refined grains intake. 95% CI, 95% confidence interval;

Study or Subgroup	Weight	Risk Ratio IV, Random, 95% CI
Ericson 2013	6.1%	1.00 [0.95, 1.05]
Fung 2002	6.4%	1.01 [0.96, 1.06]
Golozar 2017 GCS	10.7%	0.99 [0.96, 1.01]
Golozar 2017 TLGS	5.6%	1.05 [0.99, 1.11]
Hodge 2004	0.6%	1.20 [0.98, 1.47]
Kochar 2007	0.6%	0.97 [0.79, 1.18]
Liu 2000	8.6%	1.02 [0.98, 1.06]
Meyer 2000	6.5%	0.97 [0.93, 1.02]
Montonen 2003	6.7%	0.96 [0.92, 1.01]
Nanri 2010	14.8%	1.01 [1.00, 1.02]
Parker 2013	11.6%	0.97 [0.95, 1.00]
Sun 2010 HPFS rice	7.2%	1.06 [1.01, 1.11]
Sun 2010 NHS II rice	6.1%	1.05 [1.00, 1.11]
Sun 2010 NHS rice	8.5%	1.04 [1.00, 1.08]
**Total (95% CI)**	**100.0%**	**1.01 [0.99, 1.03]**

Heterogeneity: Tau² = 0.00; Chi² = 31.78, df = 13 (P = 0.003); I² = 59%
Test for overall effect: Z = 1.13 (P = 0.26)

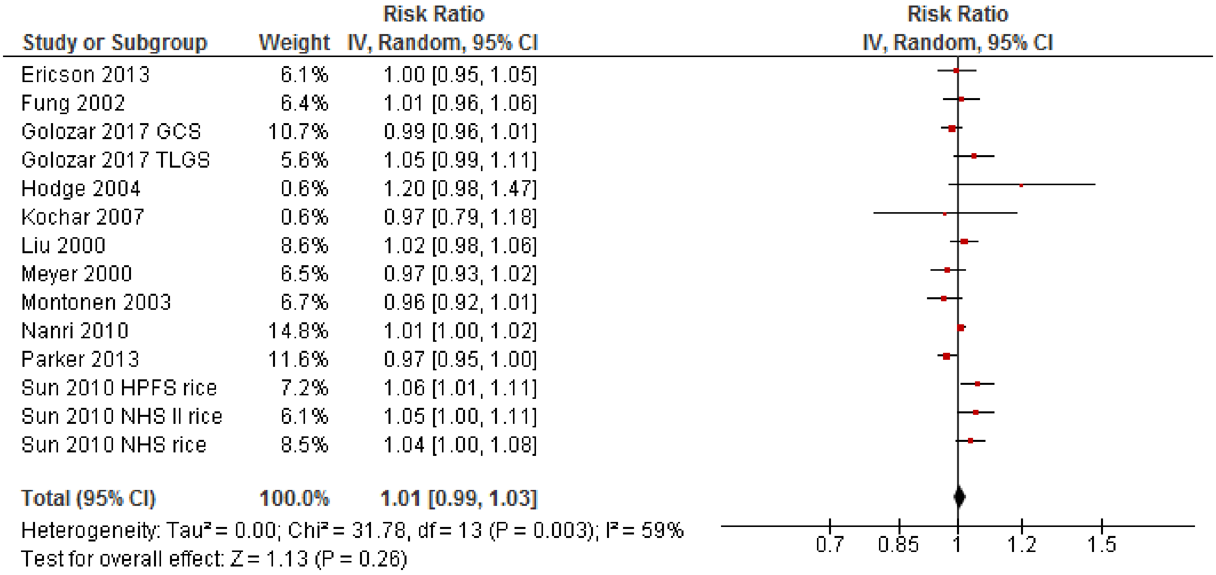

Supplementary Figure S4: Summary of relative risk of type 2 diabetes for each 30 g/d increase in refined grains intake. 95% CI, 95% confidence interval;

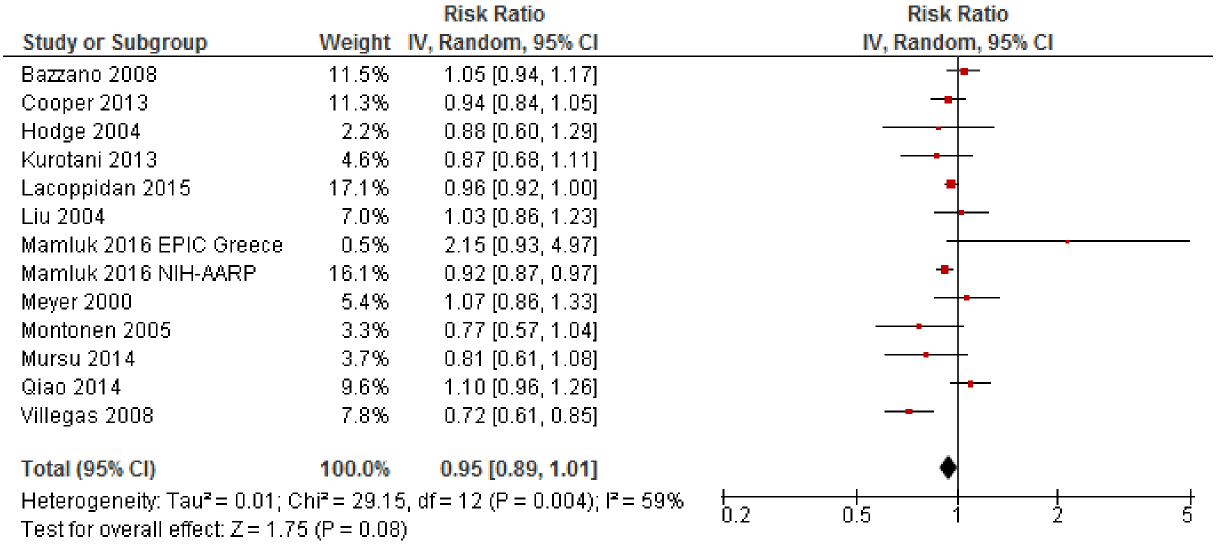

Supplementary Figure S5: Summary of relative risk of type 2 diabetes for high versus low vegetable intake. 95% CI, 95% confidence interval;

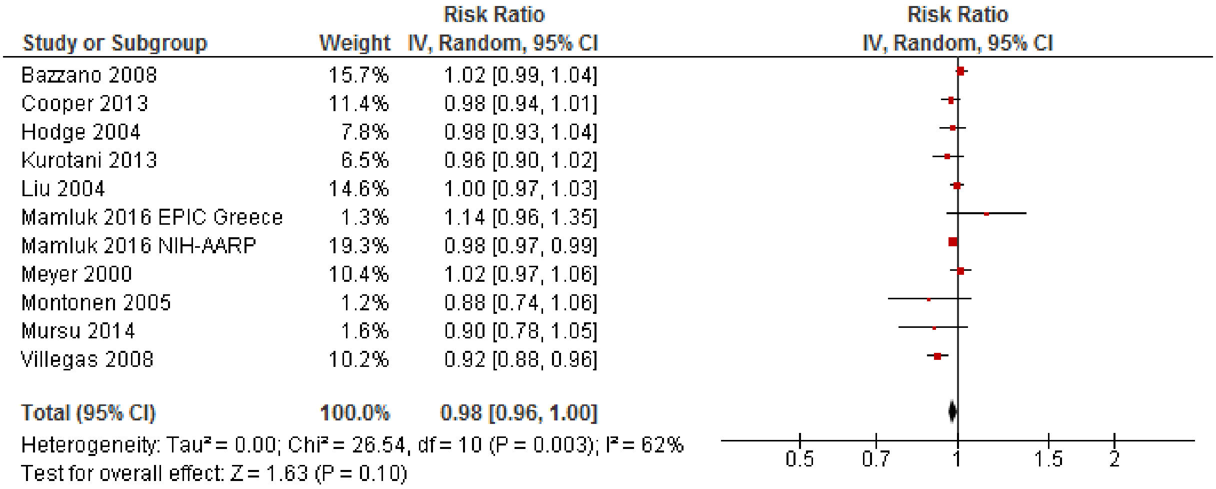

Supplementary Figure S6: Summary of relative risk of type 2 diabetes for each 100g/d increase in vegetable intake. 95% CI, 95% confidence interval;

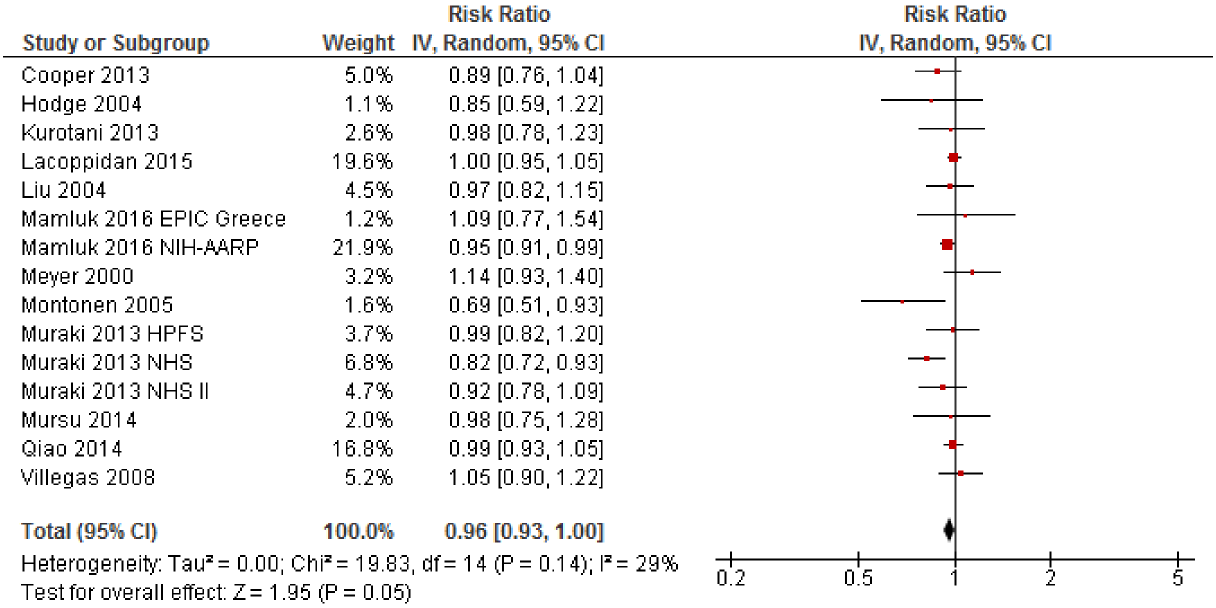

Supplementary Figure S7: Summary of relative risk of type 2 diabetes for high versus low fruit intake. 95% CI, 95% confidence interval;

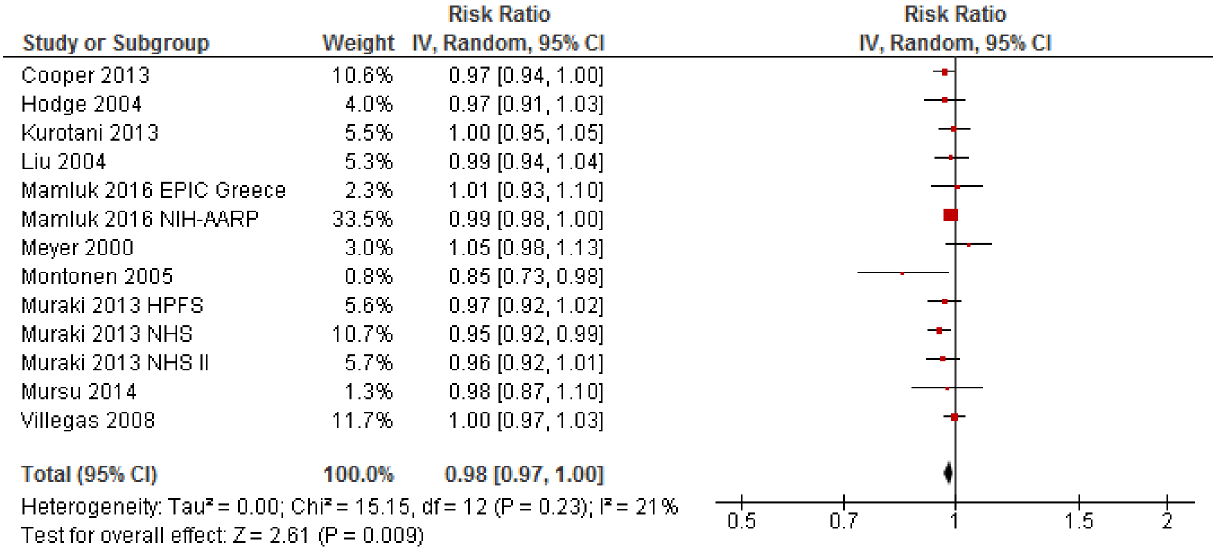

Supplementary Figure S8: Summary of relative risk of type 2 diabetes for each 100g/d increase in fruit intake. 95% CI, 95% confidence interval;

Study or Subgroup	Weight	Risk Ratio IV, Random, 95% CI
Asghari 2017	2.5%	0.47 [0.25, 0.88]
Buijsse 2015	15.2%	0.88 [0.75, 1.03]
Kochar 2010	6.5%	0.87 [0.61, 1.24]
Pan 2013 NHS I	16.6%	1.00 [0.87, 1.15]
Pan 2013 NHS II	13.8%	1.02 [0.85, 1.22]
Parker 2003	8.5%	1.51 [1.13, 2.02]
Villegas 2008	15.0%	0.80 [0.68, 0.94]
von Ruesten 2013	21.8%	0.95 [0.90, 1.00]
**Total (95% CI)**	**100.0%**	**0.95 [0.85, 1.05]**

Heterogeneity: $Tau^2 = 0.01$; $Chi^2 = 21.08$, df = 7 (P = 0.004); $I^2 = 67\%$
Test for overall effect: Z = 1.00 (P = 0.32)

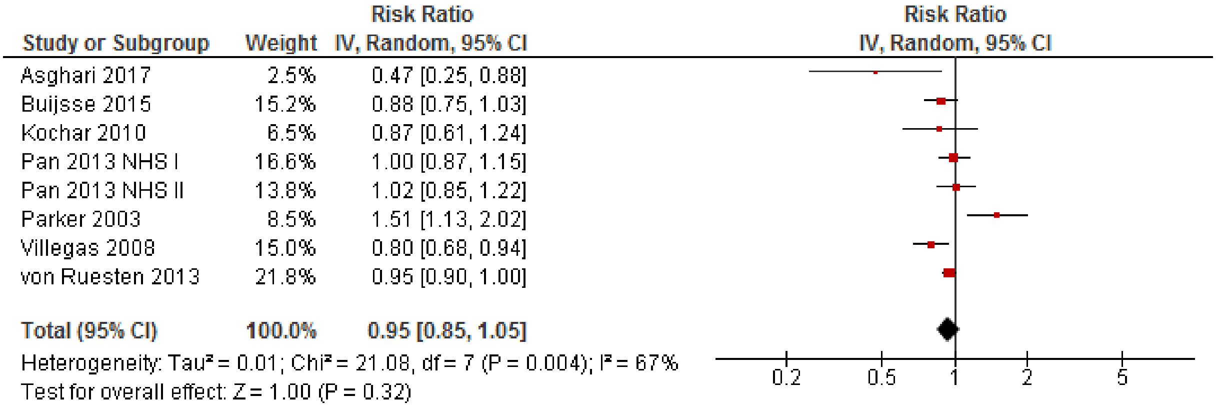

Supplementary Figure S9: Summary of relative risk of type 2 diabetes for high versus low nut intake. 95% CI, 95% confidence interval;

Study or Subgroup	Weight	Risk Ratio IV, Random, 95% CI
Asghari 2017	5.4%	0.32 [0.14, 0.75]
Kochar 2010	18.3%	0.82 [0.65, 1.04]
Pan 2013 NHS I	21.0%	1.00 [0.87, 1.15]
Pan 2013 NHS II	19.7%	1.02 [0.84, 1.23]
Parker 2003	16.8%	1.49 [1.12, 1.98]
Villegas 2008	2.6%	0.19 [0.05, 0.72]
von Ruesten 2013	16.2%	0.75 [0.55, 1.01]
**Total (95% CI)**	**100.0%**	**0.89 [0.71, 1.12]**

Heterogeneity: $Tau^2 = 0.06$; $Chi^2 = 26.15$, df = 6 (P = 0.0002); $I^2 = 77\%$
Test for overall effect: Z = 1.01 (P = 0.31)

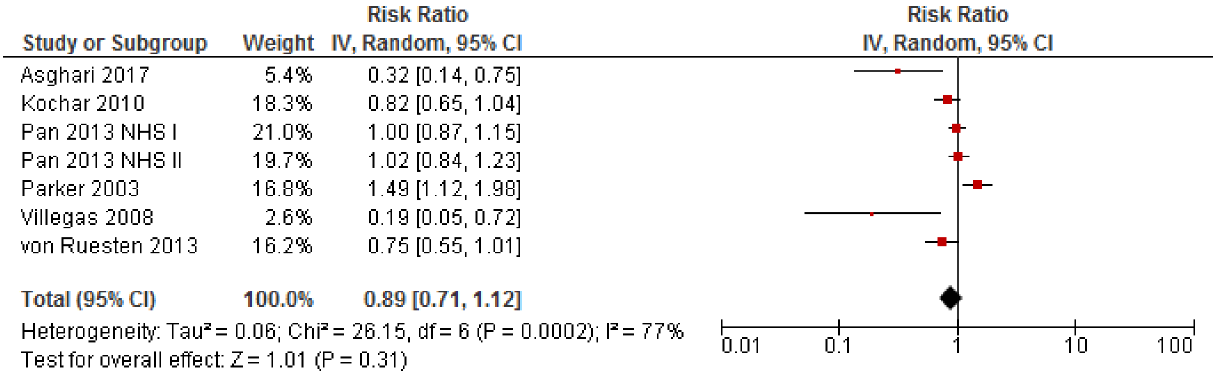

Supplementary Figure S10: Summary of relative risk of type 2 diabetes for each 28 g/d increase in nut intake. 95% CI, 95% confidence interval;

Study or Subgroup	Weight	Risk Ratio IV, Random, 95% CI
Bazzano 2008	10.4%	1.14 [1.03, 1.26]
Ding 2016 HPFS	5.6%	0.88 [0.66, 1.17]
Ding 2016 NHS	9.2%	0.97 [0.84, 1.12]
Ding 2016 NHS II	9.4%	0.92 [0.80, 1.06]
Ericson 2013	7.7%	1.00 [0.82, 1.22]
Hodge 2004	5.4%	1.09 [0.81, 1.47]
Meyer 2004	6.8%	0.96 [0.76, 1.21]
Morimoto 2011	11.4%	1.18 [1.12, 1.24]
Mueller 2012	7.7%	0.72 [0.59, 0.88]
Nanri 2010	7.1%	1.00 [0.80, 1.25]
Villegas 2008	7.8%	0.62 [0.51, 0.75]
von Ruesten 2013	11.4%	1.01 [0.96, 1.06]
**Total (95% CI)**	**100.0%**	**0.96 [0.87, 1.05]**

Heterogeneity: $Tau^2 = 0.02$; $Chi^2 = 72.82$, df = 11 (P < 0.00001); $I^2 = 85\%$
Test for overall effect: Z = 0.94 (P = 0.35)

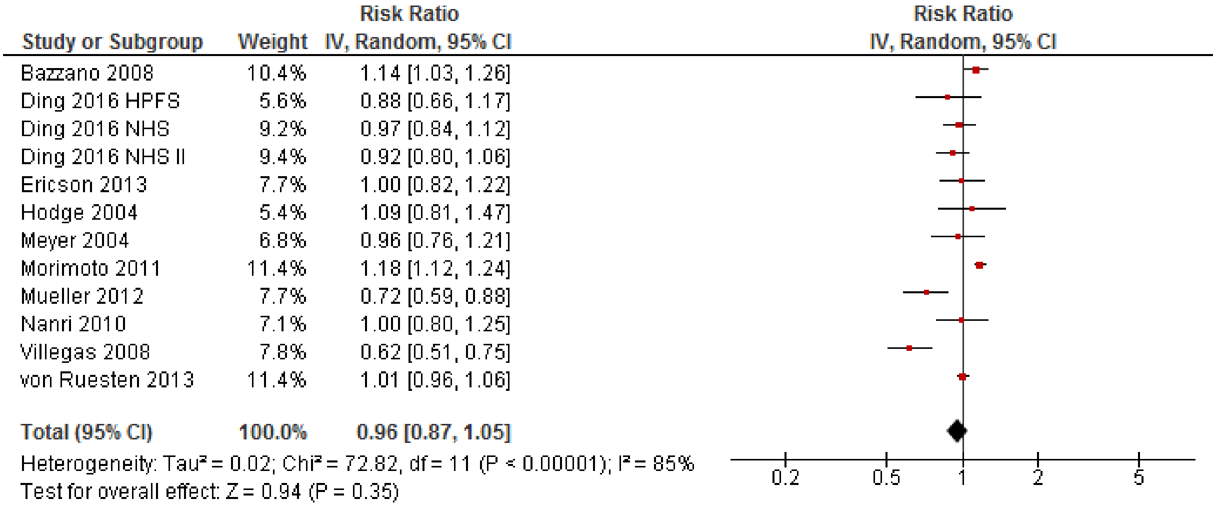

Supplementary Figure S11: Summary of relative risk of type 2 diabetes for high versus low legume intake. 95% CI, 95% confidence interval;

Study or Subgroup	Weight	Risk Ratio IV, Random, 95% CI
Bazzano 2008	11.7%	1.13 [1.02, 1.25]
Ding 2016 HPFS	0.2%	0.42 [0.04, 4.01]
Ding 2016 NHS	0.5%	0.76 [0.23, 2.51]
Ding 2016 NHS II	0.6%	0.50 [0.16, 1.56]
Ericson 2013	10.1%	1.04 [0.90, 1.20]
Hodge 2004	8.8%	1.03 [0.86, 1.23]
Meyer 2004	11.4%	0.99 [0.89, 1.11]
Morimoto 2011	6.7%	1.94 [1.52, 2.48]
Mueller 2012	12.8%	0.92 [0.86, 0.98]
Nanri 2010	13.0%	1.00 [0.94, 1.06]
Villegas 2008	10.6%	0.65 [0.57, 0.74]
von Ruesten 2013	13.7%	1.00 [0.98, 1.03]
**Total (95% CI)**	**100.0%**	**1.00 [0.92, 1.09]**

Heterogeneity: $Tau^2 = 0.01$; $Chi^2 = 83.48$, df = 11 (P < 0.00001); $I^2 = 87\%$
Test for overall effect: Z = 0.04 (P = 0.97)

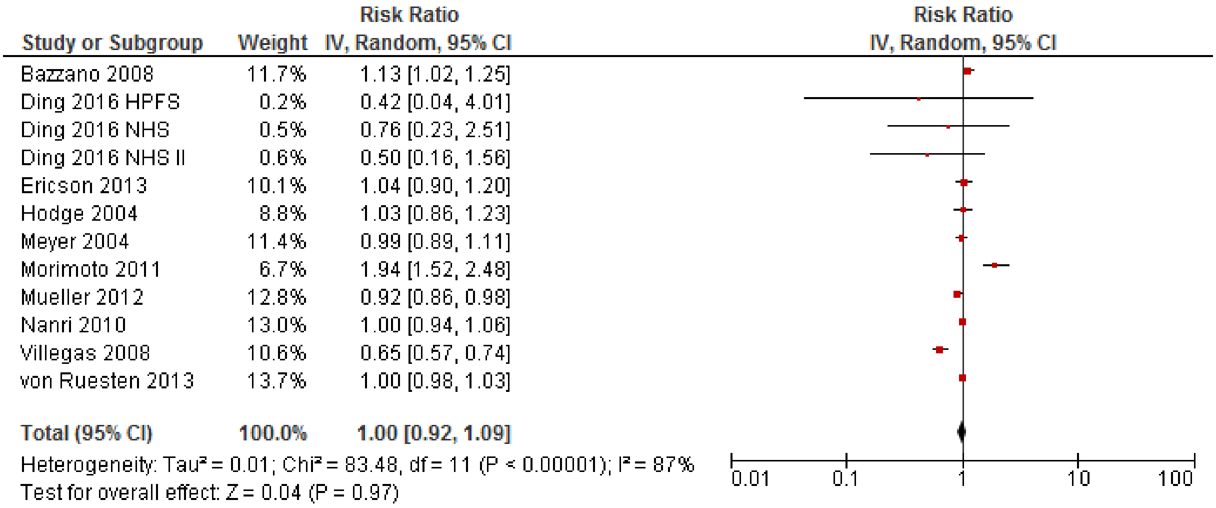

Supplementary Figure S12: Summary of relative risk of type 2 diabetes for each 50 g/d increase in legume intake. 95% CI, 95% confidence interval;

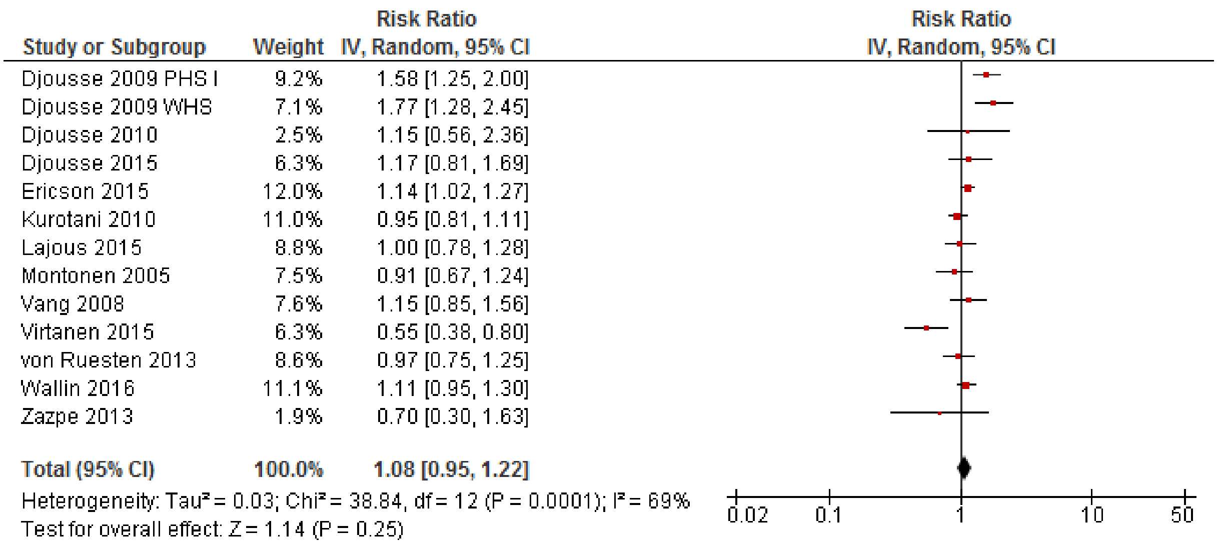

Supplementary Figure S13: Summary of relative risk of type 2 diabetes for high versus low egg intake. 95% CI, 95% confidence interval;

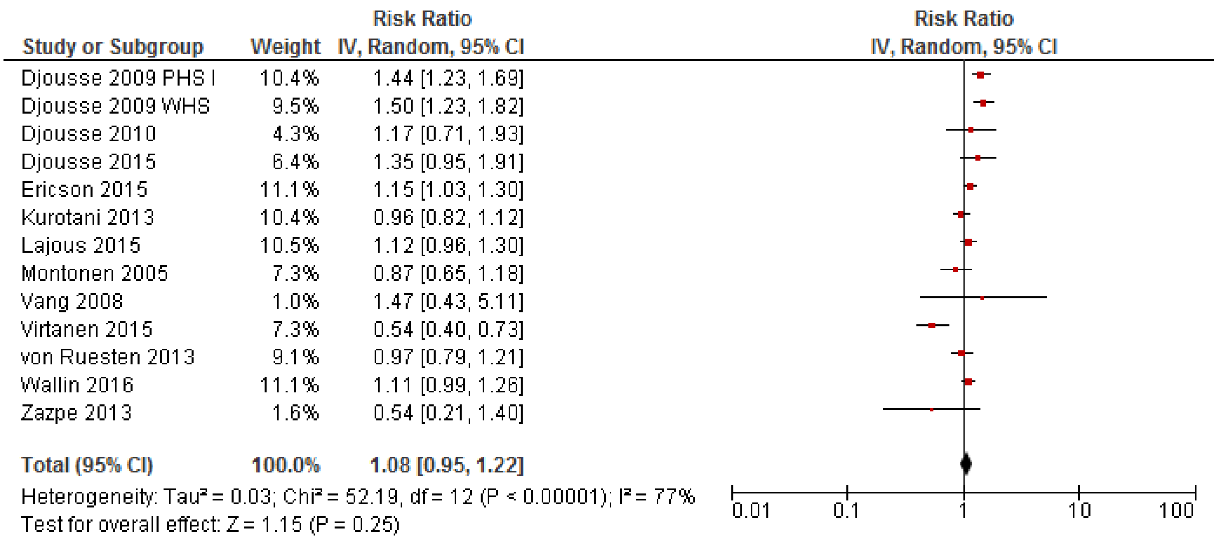

Supplementary Figure S14: Summary of relative risk of type 2 diabetes for each daily 50 g/d increase in egg intake. 95% CI, 95% confidence interval;

Study or Subgroup	Weight	Risk Ratio IV, Random, 95% CI
Brouwer-Brolsma 2016	3.2%	0.93 [0.69, 1.25]
Chen 2014 HPFS	7.3%	0.99 [0.88, 1.11]
Chen 2014 NHS	8.4%	1.05 [0.97, 1.14]
Chen 2014 NHS II	7.3%	1.00 [0.89, 1.12]
Diaz-Lopez 2015	2.4%	0.68 [0.47, 0.98]
Elwood 2007	0.4%	0.57 [0.20, 1.62]
Ericson 2015	7.3%	0.90 [0.80, 1.01]
Grantham 2012	2.2%	0.71 [0.48, 1.05]
Kirrii 2009	4.9%	0.96 [0.78, 1.18]
Liu 2006	3.7%	0.68 [0.52, 0.89]
Louie 2013	0.3%	1.50 [0.47, 4.79]
Margolis 2011	7.4%	0.93 [0.83, 1.04]
Montonen 2005	5.2%	0.90 [0.74, 1.09]
Moslehi 2015	1.8%	0.73 [0.47, 1.13]
Sluijs 2012	5.9%	0.97 [0.82, 1.15]
Soedamah-Muthu 2012	3.0%	1.30 [0.95, 1.78]
Struijk 2013	7.8%	0.95 [0.86, 1.05]
van Dam 2006	4.7%	0.93 [0.75, 1.15]
Vang 2008	5.2%	0.91 [0.75, 1.10]
Villegas 2009	7.8%	0.72 [0.65, 0.80]
Zong 2014	4.0%	0.81 [0.63, 1.04]
**Total (95% CI)**	**100.0%**	**0.91 [0.85, 0.97]**

Heterogeneity: Tau² = 0.01; Chi² = 54.56, df = 20 (P < 0.0001); I² = 63%
Test for overall effect: Z = 2.90 (P = 0.004)

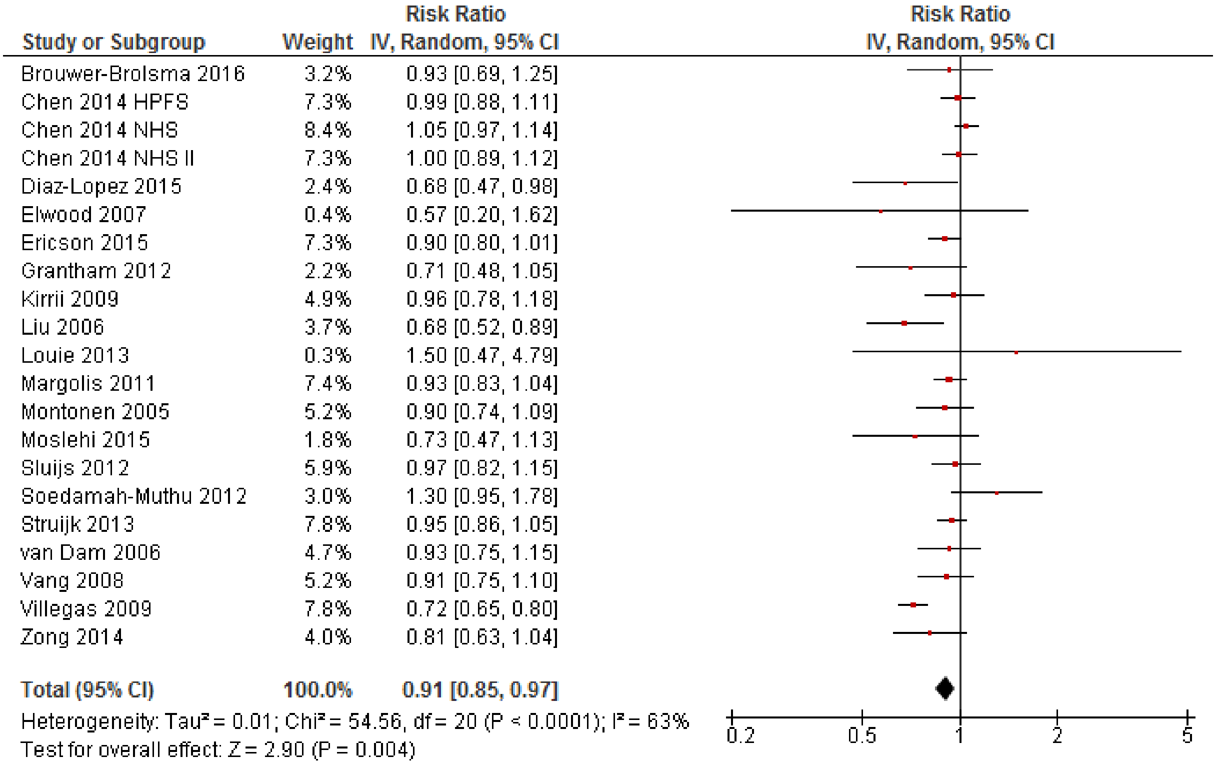

Supplementary Figure S15: Summary of relative risk of type 2 diabetes for high versus low dairy intake. 95% CI, 95% confidence interval;

Study or Subgroup	Weight	Risk Ratio IV, Random, 95% CI
Brouwer-Brolsma 2016	4.2%	0.96 [0.88, 1.05]
Chen 2014 HPFS	8.6%	0.99 [0.96, 1.02]
Chen 2014 NHS	9.2%	1.01 [0.99, 1.04]
Chen 2014 NHS II	8.9%	0.99 [0.97, 1.02]
Diaz-Lopez 2015	2.4%	0.85 [0.75, 0.97]
Elwood 2007	0.2%	0.77 [0.45, 1.31]
Ericson 2015	9.8%	0.99 [0.98, 1.00]
Grantham 2012	3.5%	0.92 [0.83, 1.01]
Kirrii 2009	3.7%	0.98 [0.89, 1.08]
Liu 2006	5.4%	0.90 [0.84, 0.96]
Louie 2013	0.4%	1.10 [0.78, 1.55]
Margolis 2011	8.5%	0.97 [0.94, 1.00]
Montonen 2005	6.7%	0.98 [0.93, 1.03]
Moslehi 2015	0.1%	0.53 [0.27, 1.04]
Sluijs 2012	6.9%	0.99 [0.94, 1.04]
Soedamah-Muthu 2012	3.2%	1.10 [0.99, 1.22]
Struijk 2013	2.4%	0.93 [0.82, 1.07]
van Dam 2006	5.7%	0.96 [0.90, 1.02]
Vang 2008	6.8%	0.99 [0.94, 1.04]
Villegas 2009	2.1%	0.61 [0.53, 0.70]
Zong 2014	1.5%	0.86 [0.72, 1.03]
**Total (95% CI)**	**100.0%**	**0.97 [0.94, 0.99]**

Heterogeneity: Tau² = 0.00; Chi² = 76.28, df = 20 (P < 0.00001); I² = 74%
Test for overall effect: Z = 2.99 (P = 0.003)

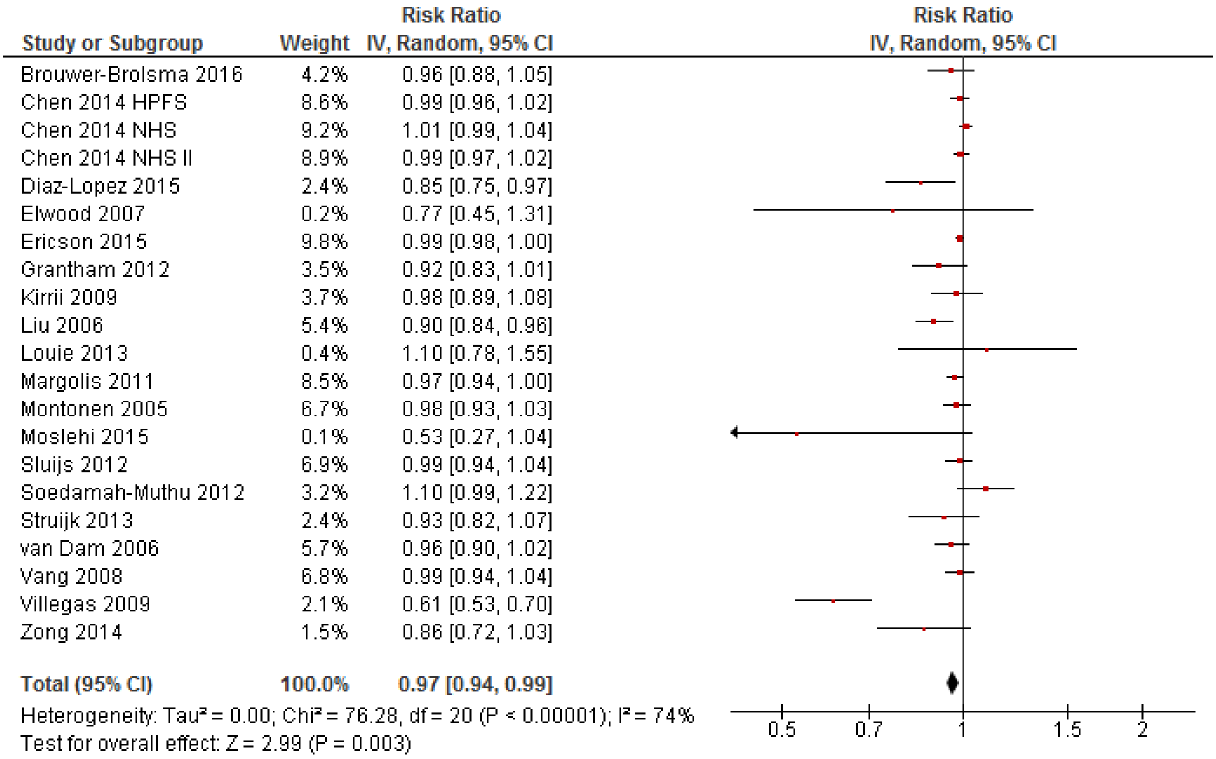

Supplementary Figure S16: Summary of relative risk of type 2 diabetes for each 200 g/d increase in dairy intake. 95% CI, 95% confidence interval;

Study or Subgroup	Weight	Risk Ratio IV, Random, 95% CI
Djousse 2011	8.2%	1.49 [1.30, 1.71]
Ericson 2015	9.4%	1.01 [0.93, 1.10]
Kaushik 2009 HPFS	6.9%	1.16 [0.96, 1.40]
Kaushik 2009 NHS	6.5%	1.29 [1.05, 1.58]
Kaushik 2009 NHS II	4.8%	1.32 [0.99, 1.76]
Krishnan 2010	4.1%	0.89 [0.64, 1.24]
Lacoppidan 2015	9.9%	1.00 [0.95, 1.05]
Montonen 2005	7.2%	0.92 [0.77, 1.10]
Nanri 2011	3.7%	0.93 [0.65, 1.33]
Patel 2012	8.1%	0.99 [0.86, 1.14]
Rylander 2015	1.7%	0.66 [0.36, 1.21]
van Woudenbergh 2009	5.4%	1.32 [1.02, 1.71]
Vang 2008	5.7%	1.12 [0.88, 1.43]
Villegas 2011 SMHS	5.0%	0.79 [0.60, 1.04]
Villegas 2011 SWHS	8.5%	0.86 [0.76, 0.97]
Wallin 2015 M	5.0%	0.79 [0.60, 1.04]
**Total (95% CI)**	**100.0%**	**1.04 [0.95, 1.13]**

Heterogeneity: Tau² = 0.02; Chi² = 62.80, df = 15 (P < 0.00001); I² = 76%
Test for overall effect: Z = 0.85 (P = 0.40)

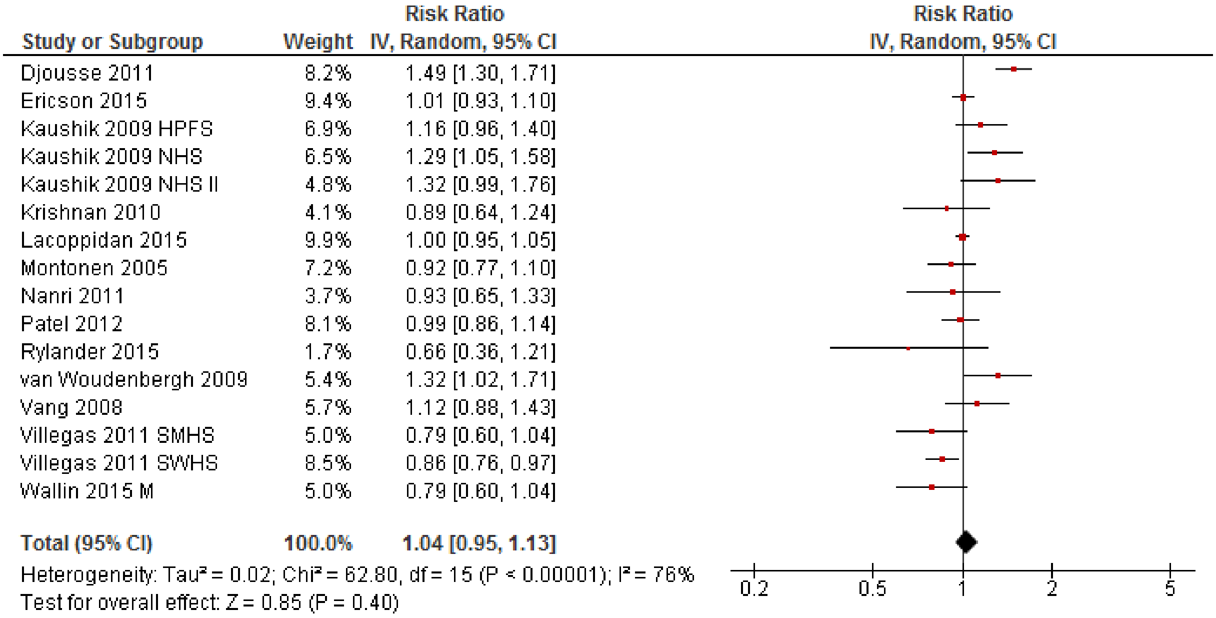

Supplementary Figure S17: Summary of relative risk of type 2 diabetes for high versus low fish intake. 95% CI, 95% confidence interval;

Study or Subgroup	Weight	Risk Ratio IV, Random, 95% CI
Djousse 2011	8.4%	2.05 [1.64, 2.56]
Ericson 2015	9.3%	0.98 [0.85, 1.13]
Kaushik 2009 HPFS	8.9%	1.25 [1.04, 1.50]
Kaushik 2009 NHS	9.1%	1.33 [1.13, 1.57]
Kaushik 2009 NHS II	8.3%	1.48 [1.18, 1.87]
Krishnan 2010	3.4%	0.91 [0.46, 1.82]
Montonen 2005	2.5%	0.71 [0.30, 1.68]
Nanri 2011	9.3%	0.89 [0.77, 1.03]
Patel 2012	8.7%	1.00 [0.82, 1.22]
Rylander 2015	1.4%	0.52 [0.15, 1.75]
van Woudenbergh 2009	3.6%	1.99 [1.02, 3.88]
Vang 2008	1.5%	1.40 [0.43, 4.53]
Villegas 2011 SMHS	8.4%	0.91 [0.73, 1.13]
Villegas 2011 SWHS	9.5%	0.84 [0.74, 0.95]
Wallin 2015 M	7.4%	0.73 [0.54, 0.98]
**Total (95% CI)**	**100.0%**	**1.09 [0.93, 1.28]**

Heterogeneity: Tau² = 0.06; Chi² = 86.74, df = 14 (P < 0.00001); I² = 84%
Test for overall effect: Z = 1.07 (P = 0.29)

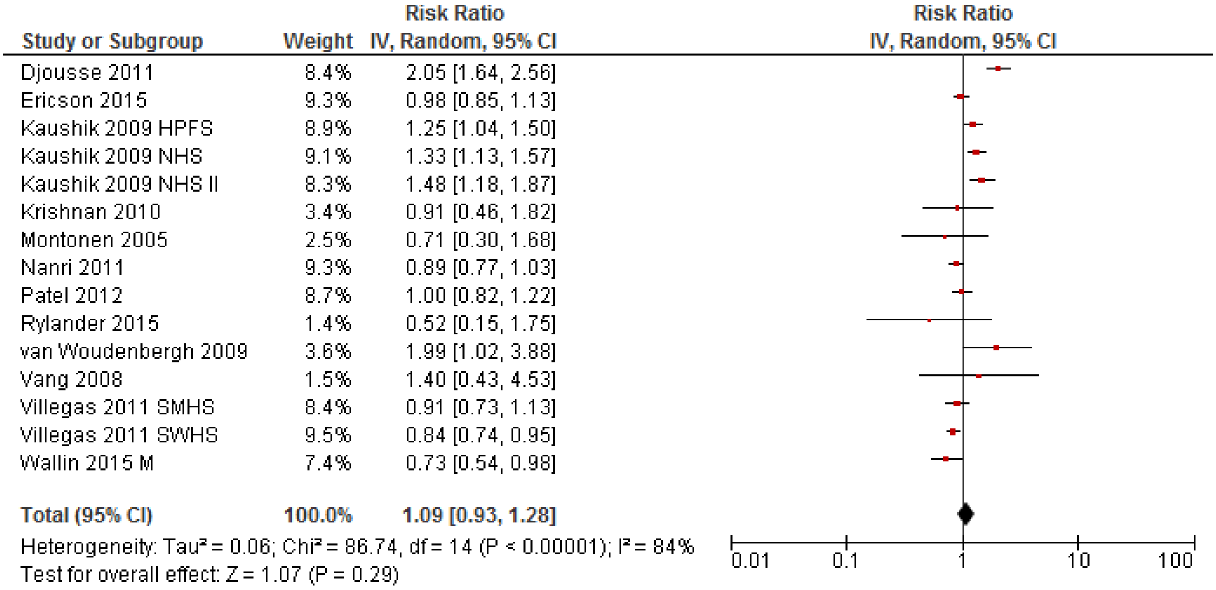

Supplementary Figure S18: Summary of relative risk of type 2 diabetes for each 100 g/d increase in fish intake. 95% CI, 95% confidence interval;

Study or Subgroup	Weight	Risk Ratio IV, Random, 95% CI
Ericson 2015	11.2%	1.11 [1.03, 1.20]
Fretts 2012	2.1%	0.88 [0.57, 1.36]
Interact 2013	9.5%	1.20 [1.07, 1.35]
Kurotani 2013	4.7%	1.30 [1.01, 1.67]
Lee 2004	6.0%	1.19 [0.97, 1.46]
Mari-Sanchis 2016	2.1%	0.95 [0.62, 1.46]
Montonen 2005	3.4%	0.99 [0.72, 1.36]
Männistö 2010	5.3%	1.22 [0.97, 1.53]
Pan 2011 HPFS	7.7%	1.44 [1.23, 1.69]
Pan 2011 NHS	11.0%	1.31 [1.21, 1.42]
Pan 2011 NHS II	8.4%	1.37 [1.19, 1.58]
Song 2004	5.7%	1.24 [1.00, 1.54]
Steinbrecher 2011	11.5%	1.36 [1.27, 1.46]
van Woudenbergh 2012	3.8%	1.18 [0.88, 1.58]
Villegas 2006	7.5%	0.94 [0.80, 1.10]
**Total (95% CI)**	**100.0%**	**1.21 [1.13, 1.30]**

Heterogeneity: Tau² = 0.01; Chi² = 40.50, df = 14 (P = 0.0002); I² = 65%
Test for overall effect: Z = 5.49 (P < 0.00001)

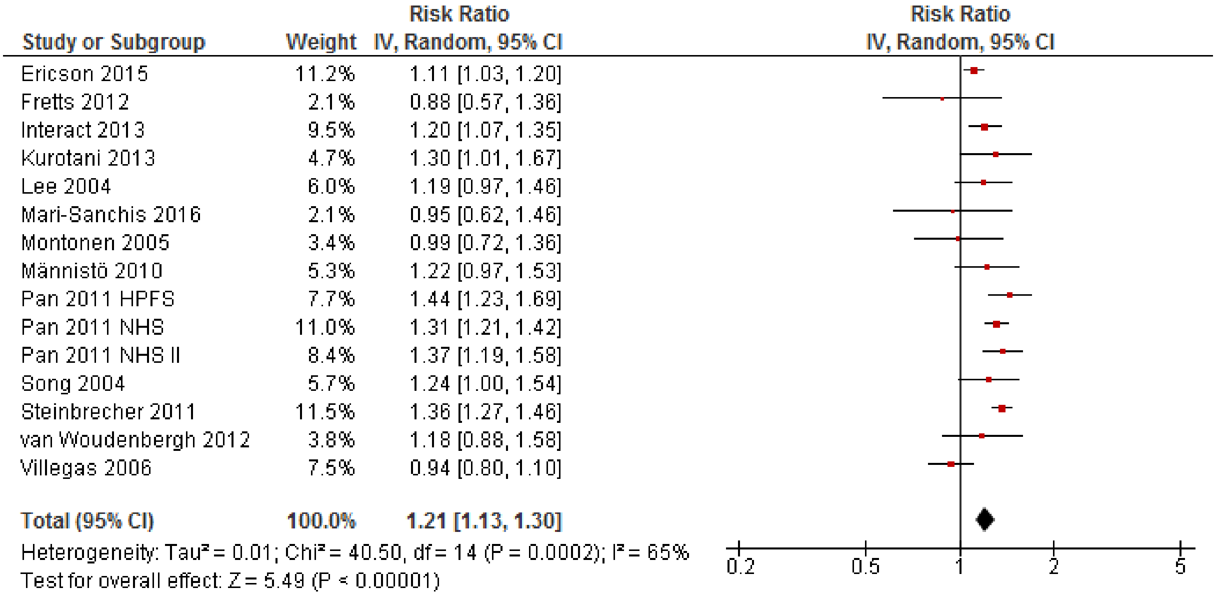

Supplementary Figure S19: Summary of relative risk of type 2 diabetes for high versus low red meat intake. 95% CI, 95% confidence interval;

Study or Subgroup	Weight	Risk Ratio IV, Random, 95% CI
Ericson 2015	8.2%	1.24 [1.08, 1.42]
Fretts 2012	1.8%	0.80 [0.47, 1.35]
Interact 2013	9.0%	1.26 [1.13, 1.41]
Kurotani 2013	4.8%	1.33 [1.02, 1.73]
Mari-Sanchis 2016	4.5%	0.92 [0.69, 1.21]
Montonen 2003	4.9%	0.94 [0.72, 1.22]
Männistö 2010	6.2%	1.19 [0.97, 1.46]
Pan 2011 HPFS	10.2%	1.21 [1.13, 1.30]
Pan 2011 NHS	10.9%	1.14 [1.10, 1.18]
Pan 2011 NHS II	10.2%	1.19 [1.11, 1.28]
Song 2004	8.0%	1.19 [1.03, 1.37]
Steinbrecher 2011	9.5%	1.64 [1.49, 1.80]
van Woudenbergh 2009	5.3%	1.12 [0.88, 1.43]
Villegas 2006	6.4%	0.81 [0.66, 0.98]
**Total (95% CI)**	**100.0%**	**1.17 [1.08, 1.26]**

Heterogeneity: Tau² = 0.01; Chi² = 74.50, df = 13 (P < 0.00001); I² = 83%
Test for overall effect: Z = 3.98 (P < 0.0001)

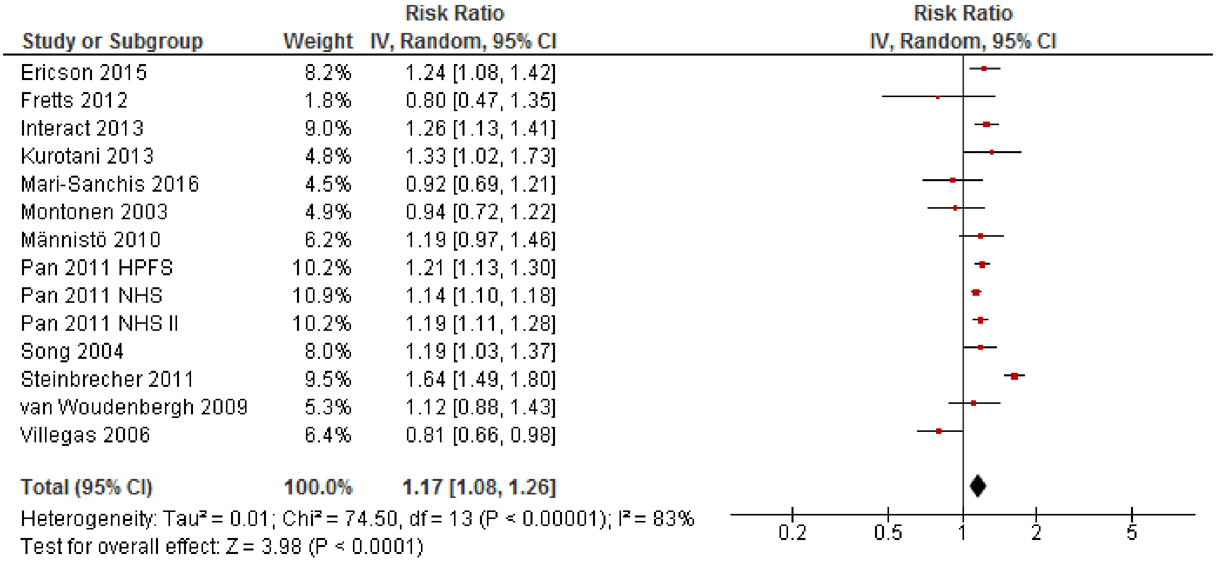

Supplementary Figure S20: Summary of relative risk of type 2 diabetes for each 100 g/d increase in red meat intake. 95% CI, 95% confidence interval;

Study or Subgroup	Weight	Risk Ratio IV, Random, 95% CI
Ericson 2015	12.1%	1.16 [1.06, 1.27]
Fretts 2012	1.3%	1.35 [0.81, 2.25]
Interact 2013	10.7%	1.16 [1.04, 1.29]
Kurotani 2013	6.3%	1.11 [0.92, 1.34]
Mari-Sanchis 2016	0.7%	1.60 [0.78, 3.28]
Montonen 2005	2.9%	1.22 [0.89, 1.67]
Männistö 2010	5.4%	1.37 [1.11, 1.69]
Pan 2011 HPFS	8.0%	1.55 [1.33, 1.81]
Pan 2011 NHS	12.8%	1.30 [1.20, 1.41]
Pan 2011 NHS II	9.8%	1.21 [1.07, 1.37]
Song 2004	6.9%	1.19 [1.00, 1.42]
Steinbrecher 2011	14.3%	1.39 [1.31, 1.47]
van Woudenbergh 2012	2.0%	1.73 [1.16, 2.58]
Villegas 2006	6.8%	1.18 [0.99, 1.41]
**Total (95% CI)**	**100.0%**	**1.27 [1.20, 1.35]**

Heterogeneity: $Tau^2 = 0.01$; $Chi^2 = 28.82$, df = 13 (P = 0.007); $I^2 = 55\%$
Test for overall effect: Z = 7.84 (P < 0.00001)

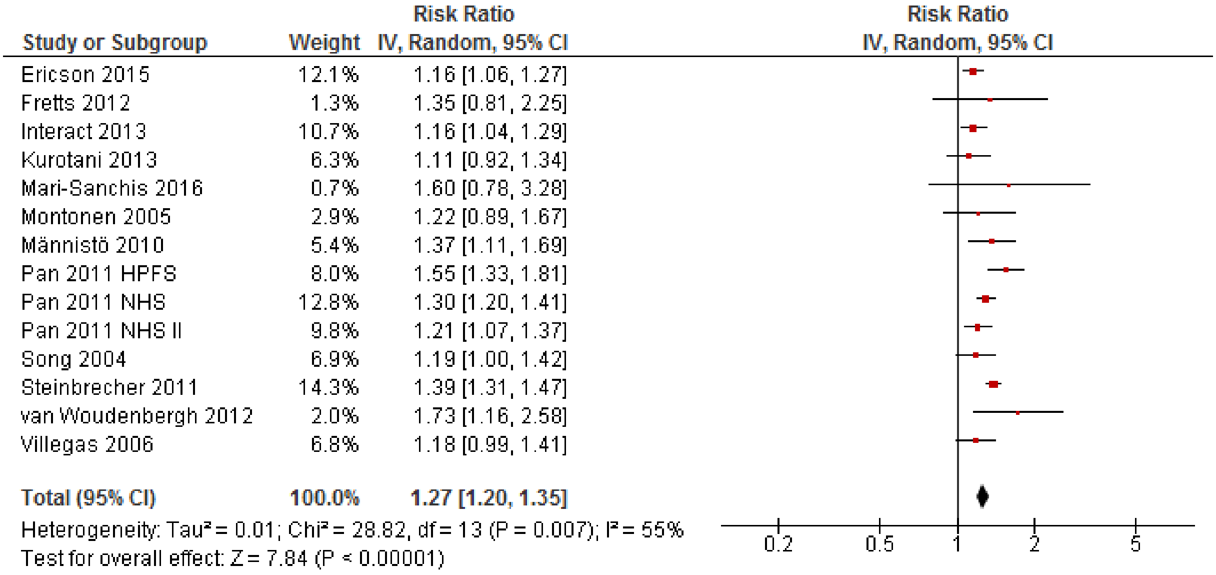

Supplementary Figure S21: Summary of relative risk of type 2 diabetes for high versus low processed meat intake. 95% CI, 95% confidence interval;

Study or Subgroup	Weight	Risk Ratio IV, Random, 95% CI
Ericson 2015	9.4%	1.19 [1.08, 1.31]
Fretts 2012	3.4%	1.37 [0.81, 2.30]
Interact 2013	9.7%	1.10 [1.03, 1.17]
Kurotani 2013	2.7%	1.51 [0.82, 2.78]
Mari-Sanchis 2016	7.5%	1.20 [0.96, 1.49]
Montonen 2003	8.1%	1.15 [0.96, 1.38]
Männistö 2010	9.7%	1.12 [1.04, 1.20]
Pan 2011 HPFS	8.1%	1.81 [1.51, 2.17]
Pan 2011 NHS	9.2%	1.50 [1.34, 1.67]
Pan 2011 NHS II	7.2%	1.59 [1.26, 2.01]
Song 2004	6.2%	1.44 [1.07, 1.93]
Steinbrecher 2011	9.1%	1.90 [1.69, 2.14]
van Woudenbergh 2009	3.9%	1.61 [1.01, 2.57]
Villegas 2006	5.8%	1.30 [0.95, 1.79]
**Total (95% CI)**	**100.0%**	**1.37 [1.22, 1.55]**

Heterogeneity: $Tau^2 = 0.04$; $Chi^2 = 107.02$, df = 13 (P < 0.00001); $I^2 = 88\%$
Test for overall effect: Z = 5.25 (P < 0.00001)

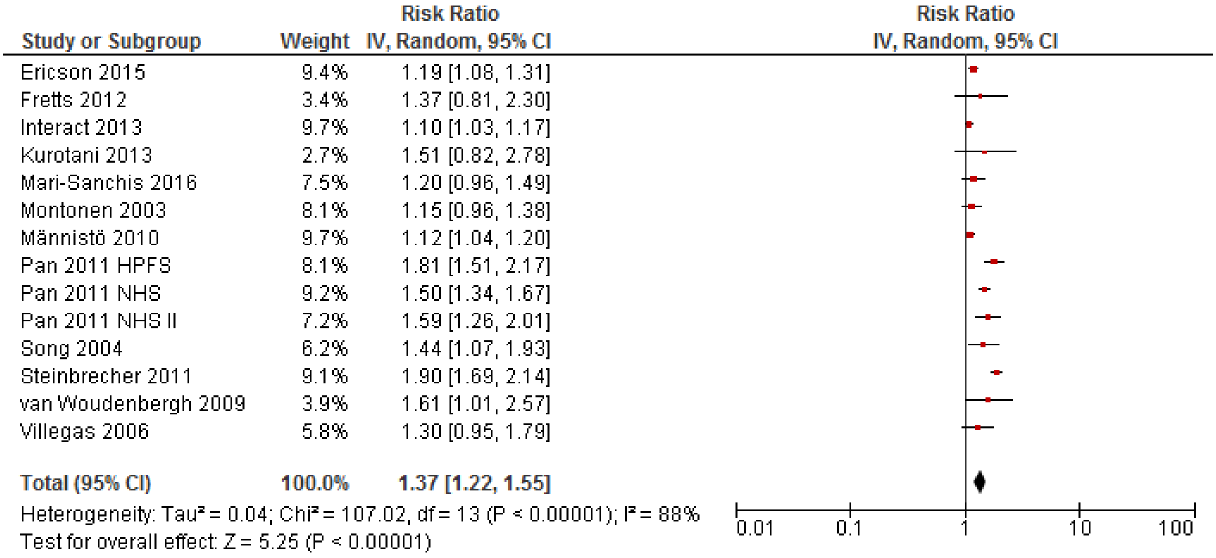

Supplementary Figure S22: Summary of relative risk of type 2 diabetes for each 50 g/d increase in processed meat intake. 95% CI, 95% confidence interval;

Study or Subgroup	Weight	Risk Ratio IV, Random, 95% CI
Bhupathiraju 2013 HPFS	14.4%	1.35 [1.16, 1.57]
Bhupathiraju 2013 NHS	21.1%	1.26 [1.14, 1.39]
Eshak 2013	6.0%	1.22 [0.92, 1.62]
Interact 2013	8.0%	1.29 [1.02, 1.63]
Montonen 2007	1.9%	1.60 [0.93, 2.75]
Odegaard 2010	16.2%	1.34 [1.17, 1.53]
Palmer 2008	13.8%	1.24 [1.06, 1.45]
Paytner 2006	10.0%	1.04 [0.85, 1.27]
Sakurai 2014	1.4%	1.34 [0.72, 2.49]
Schulze 2003	7.1%	1.83 [1.42, 2.36]
**Total (95% CI)**	**100.0%**	**1.30 [1.20, 1.40]**

Heterogeneity: $Tau^2 = 0.00$; $Chi^2 = 13.56$, df = 9 (P = 0.14); $I^2 = 34\%$
Test for overall effect: Z = 6.71 (P < 0.00001)

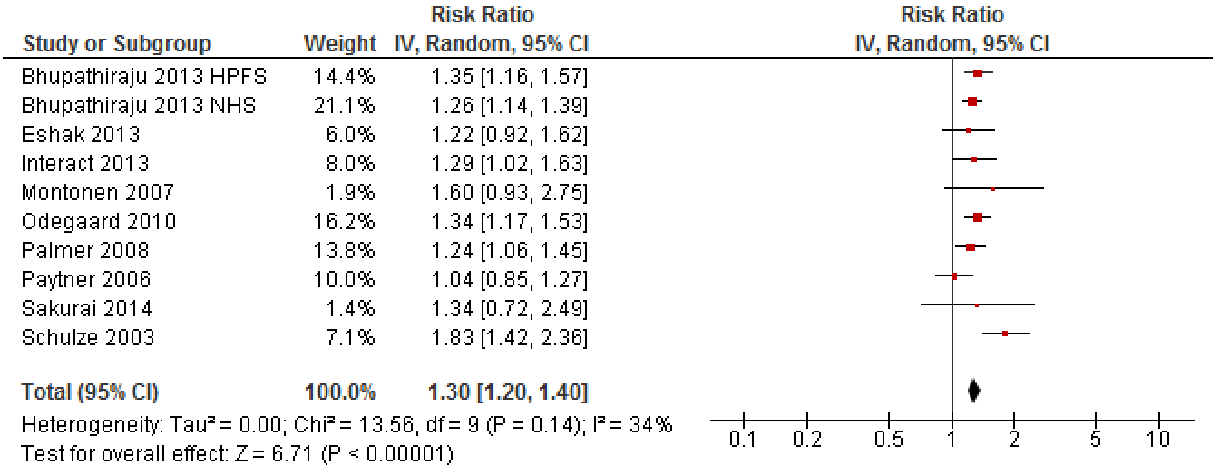

Supplementary Figure S23: Summary of relative risk of type 2 diabetes for high versus low sugar sweetened beverage intake. 95% CI, 95% confidence interval;

Study or Subgroup	Weight	Risk Ratio IV, Random, 95% CI
Bhupathiraju 2013 HPFS	13.7%	1.25 [1.14, 1.37]
Bhupathiraju 2013 NHS	15.4%	1.16 [1.09, 1.23]
Eshak 2013	5.4%	1.13 [0.86, 1.48]
Interact 2013	14.4%	1.15 [1.06, 1.25]
Montonen 2007	0.9%	2.81 [1.30, 6.09]
Odegaard 2010	8.7%	1.50 [1.25, 1.80]
Palmer 2008	16.1%	1.10 [1.05, 1.15]
Paytner 2006	14.4%	1.03 [0.95, 1.12]
Sakurai 2014	2.9%	1.25 [0.83, 1.89]
Schulze 2004	8.2%	1.64 [1.35, 1.98]
**Total (95% CI)**	**100.0%**	**1.21 [1.12, 1.31]**

Heterogeneity: $Tau^2 = 0.01$; $Chi^2 = 40.36$, df = 9 (P < 0.00001); $I^2 = 78\%$
Test for overall effect: Z = 4.92 (P < 0.00001)

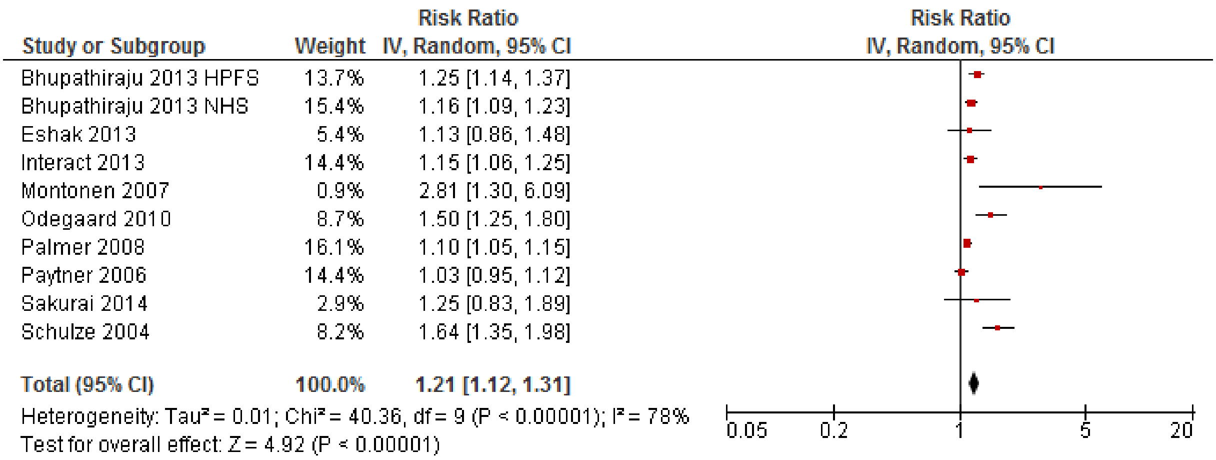

Supplementary Figure S24: Summary of relative risk of type 2 diabetes for each 250 ml/d increase in sugar sweetened beverages intake. 95% CI, 95% confidence interval;

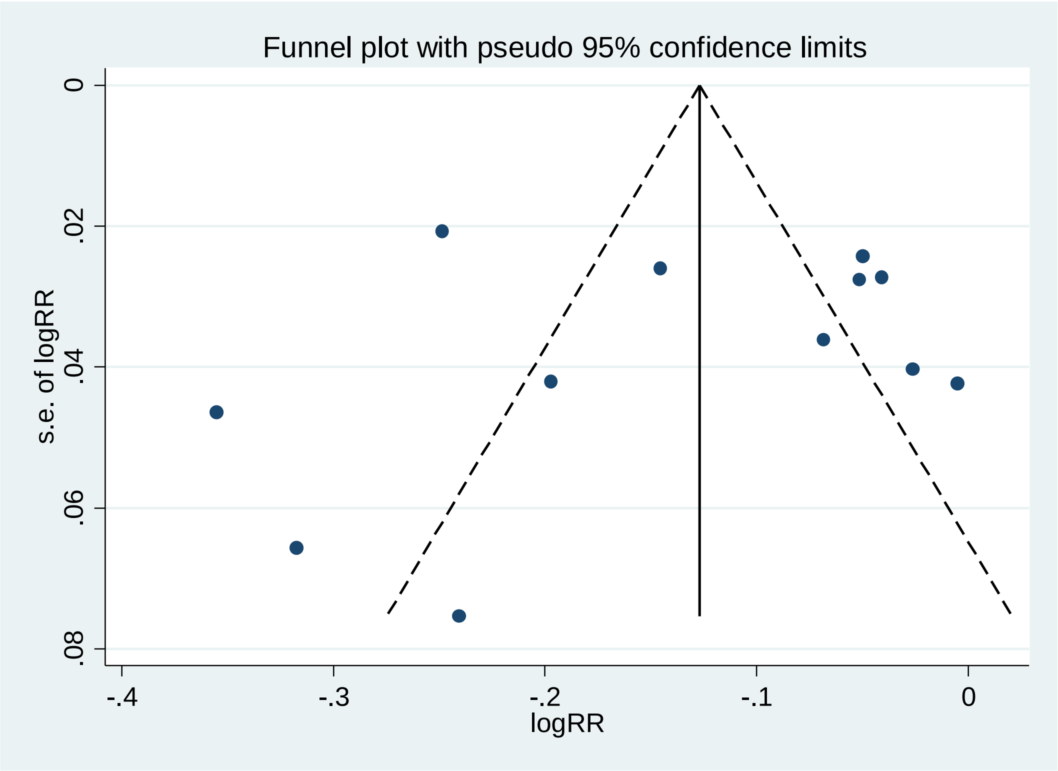

Supplementary Figure S25: Funnel plot showing study precision against the relative risk with 95% CIs for whole grain intake (dose-response meta-analysis) and risk of T2D. SE = Standard error

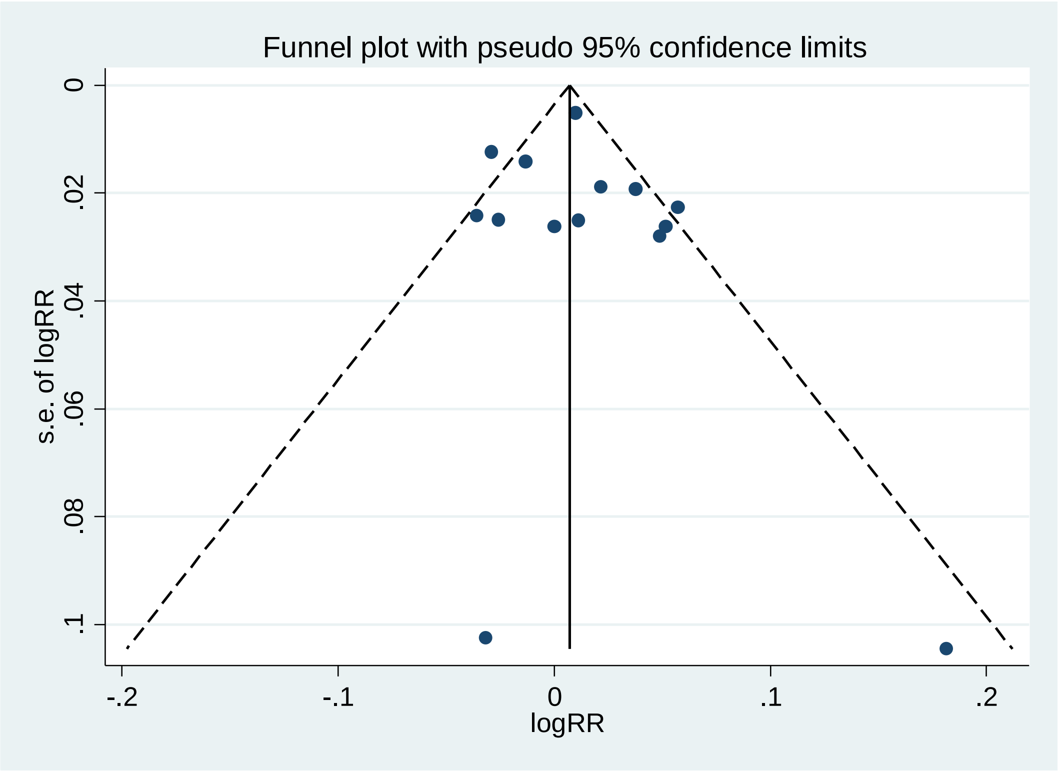

Supplementary Figure S26: Funnel plot showing study precision against the relative risk with 95% CIs for refined grain intake (dose-response meta-analysis) and risk of T2D. SE = Standard error

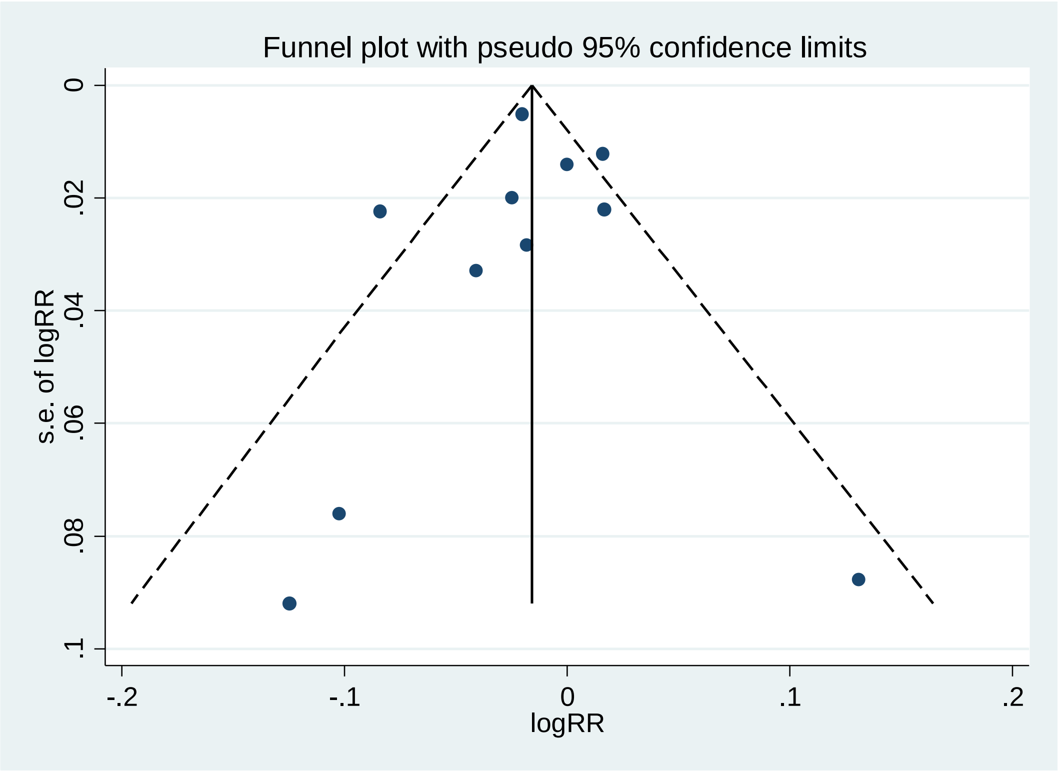

Supplementary Figure S27: Funnel plot showing study precision against the relative risk with 95% CIs for vegetable intake (dose-response meta-analysis) and risk of T2D. SE = Standard error

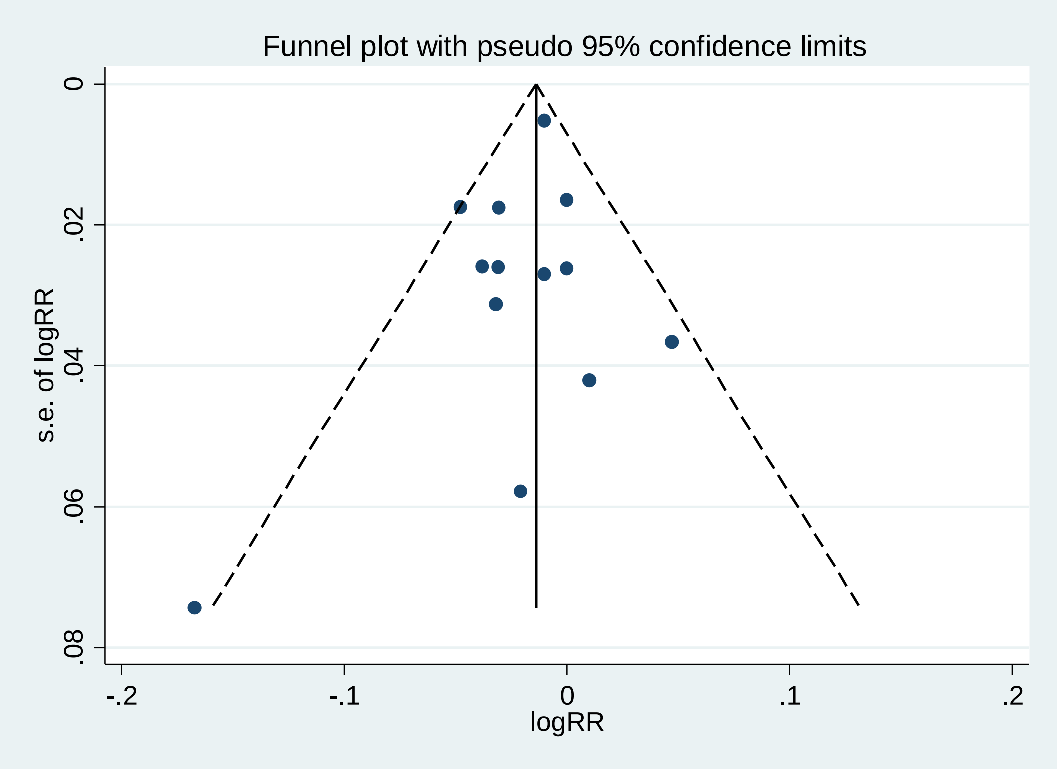

Supplementary Figure S28: Funnel plot showing study precision against the relative risk with 95% CIs for fruit intake (dose-response meta-analysis) and risk of T2D. SE = Standard error

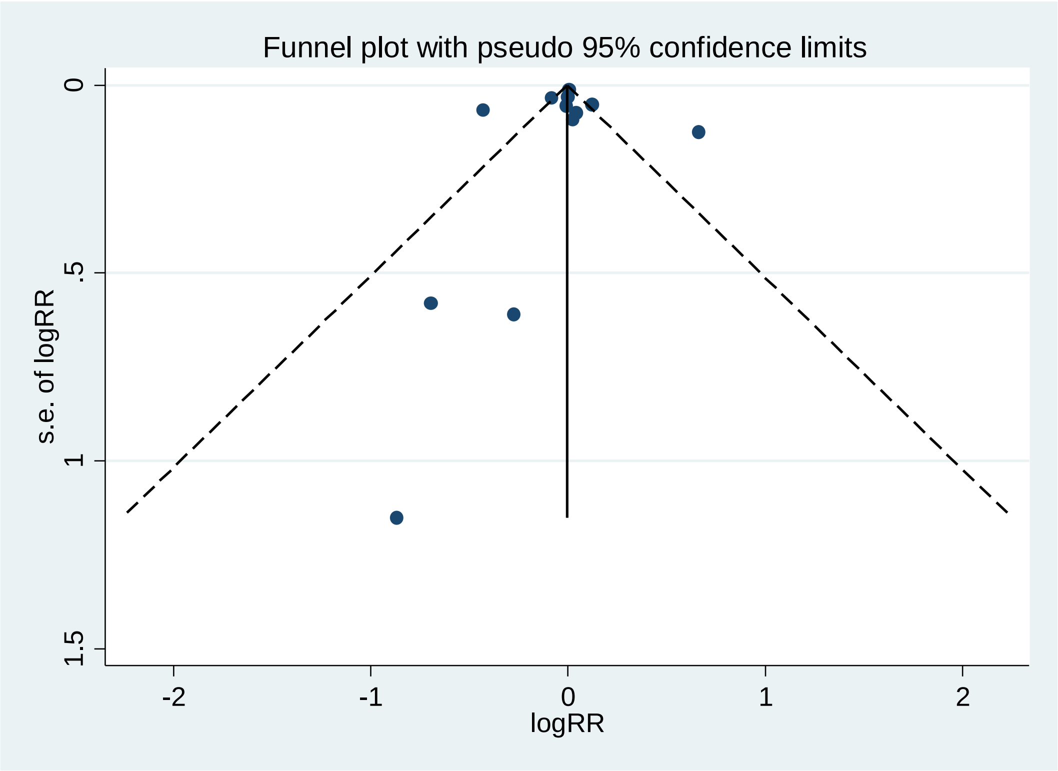

Supplementary Figure S29: Funnel plot showing study precision against the relative risk with 95% CIs for legumes intake (dose-response meta-analysis) and risk of T2D. SE = Standard error

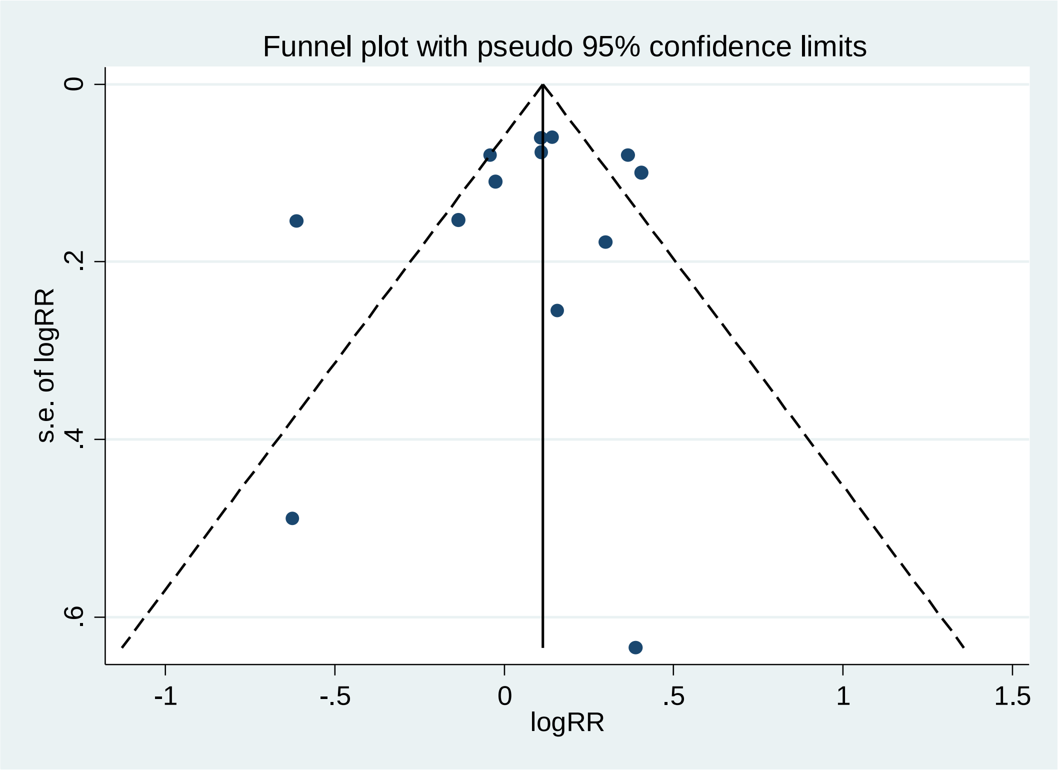

Supplementary Figure S30: Funnel plot showing study precision against the relative risk with 95% CIs for egg intake (dose-response meta-analysis) and risk of T2D. SE = Standard error

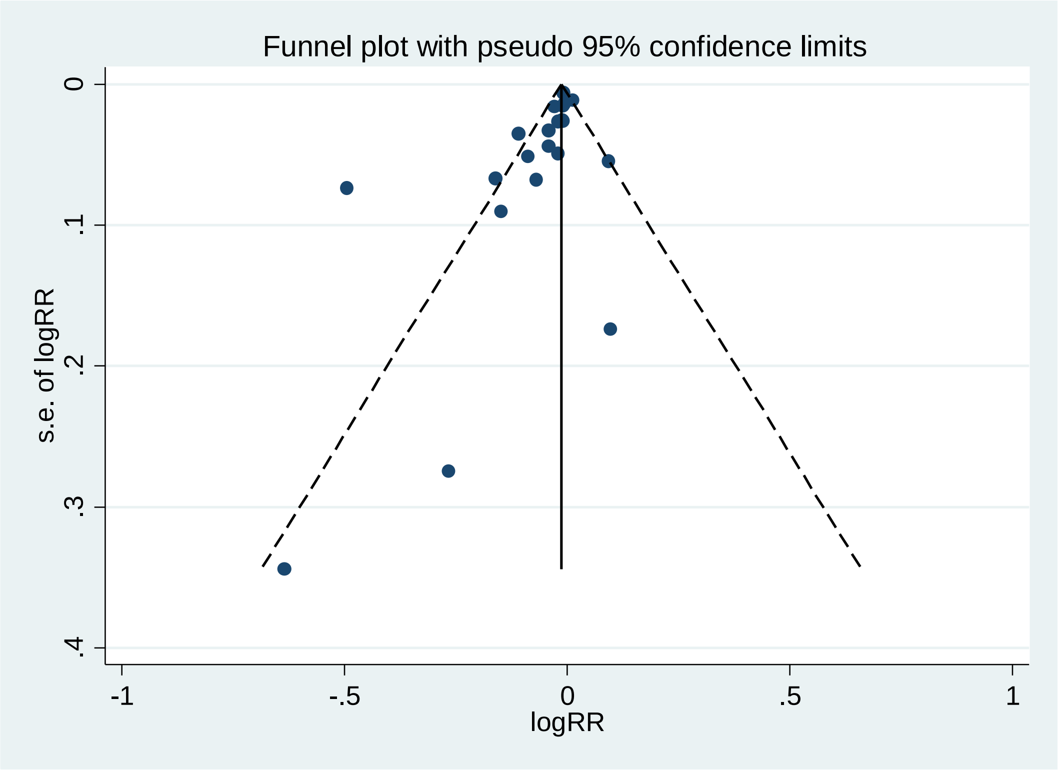

Supplementary Figure S31: Funnel plot showing study precision against the relative risk with 95% CIs for dairy intake (dose-response meta-analysis) and risk of T2D. SE = Standard error

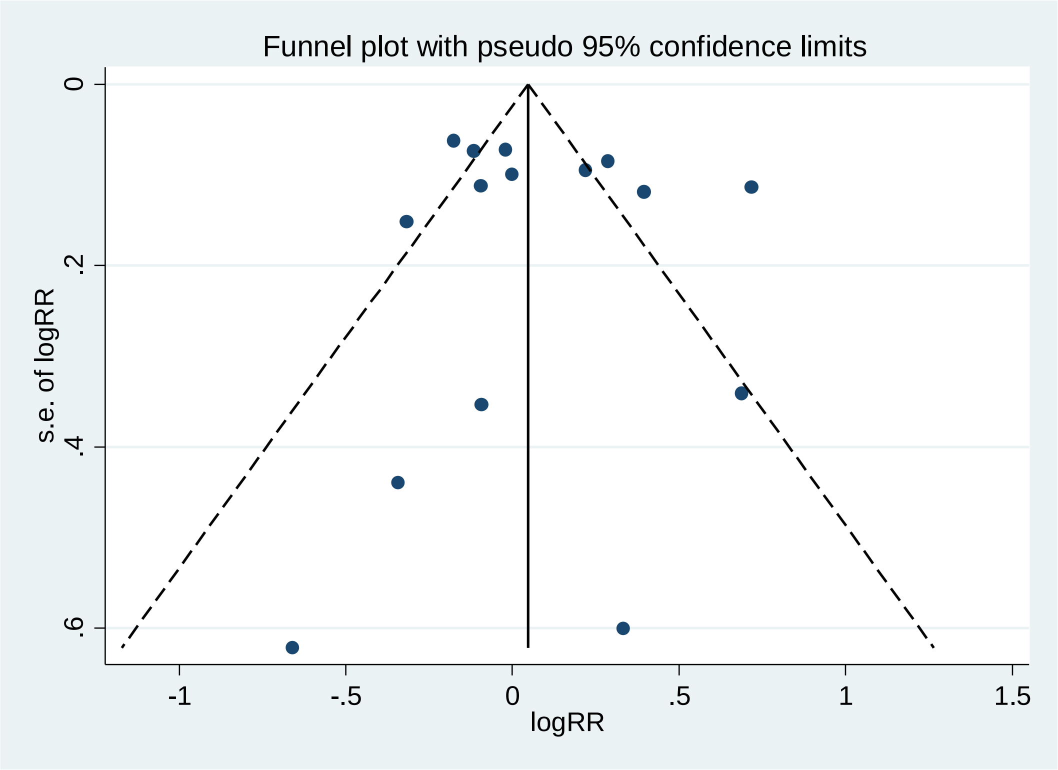

Supplementary Figure S32: Funnel plot showing study precision against the relative risk with 95% CIs for fish intake (dose-response meta-analysis) and risk of T2D. SE = Standard error

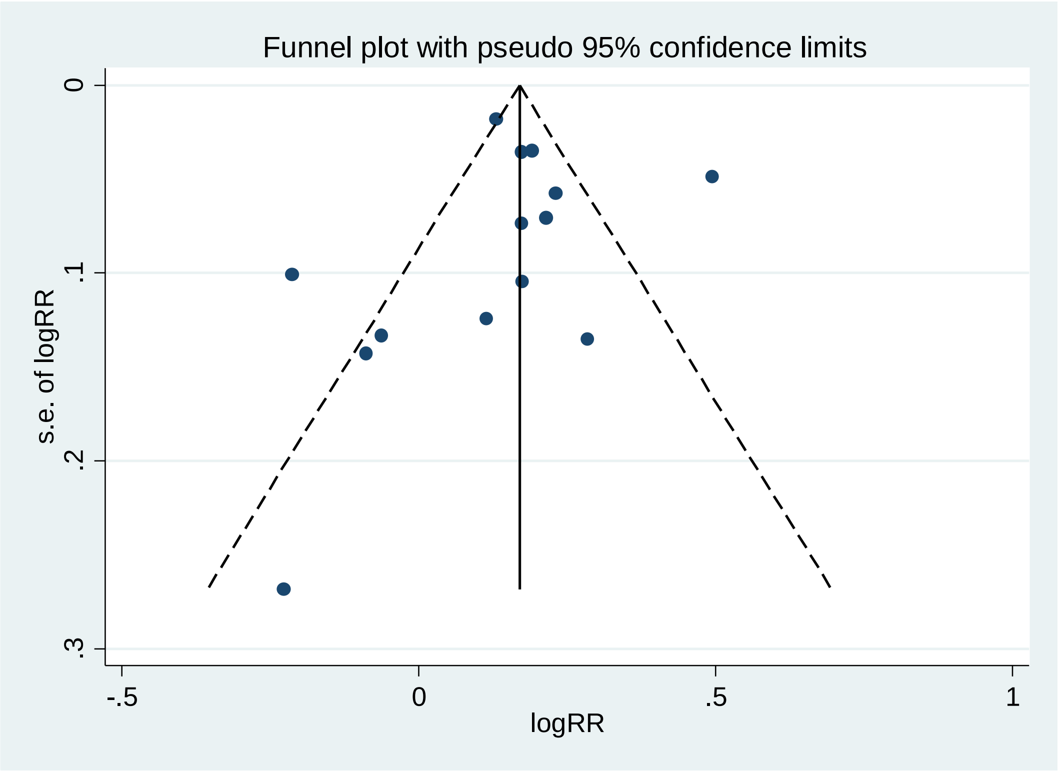

Supplementary Figure S33: Funnel plot showing study precision against the relative risk with 95% CIs for red meat intake (dose-response meta-analysis) and risk of T2D. SE = Standard error

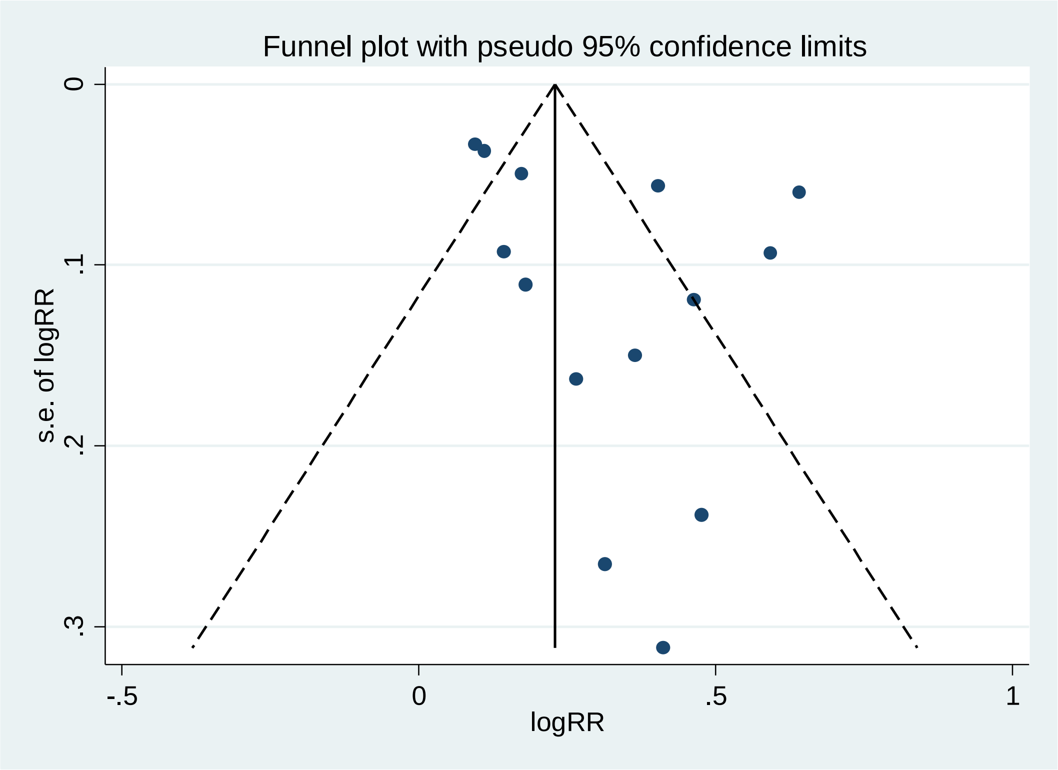

Supplementary Figure S34: Funnel plot showing study precision against the relative risk with 95% CIs for processed meat intake (dose-response meta-analysis) and risk of T2D. SE = Standard error

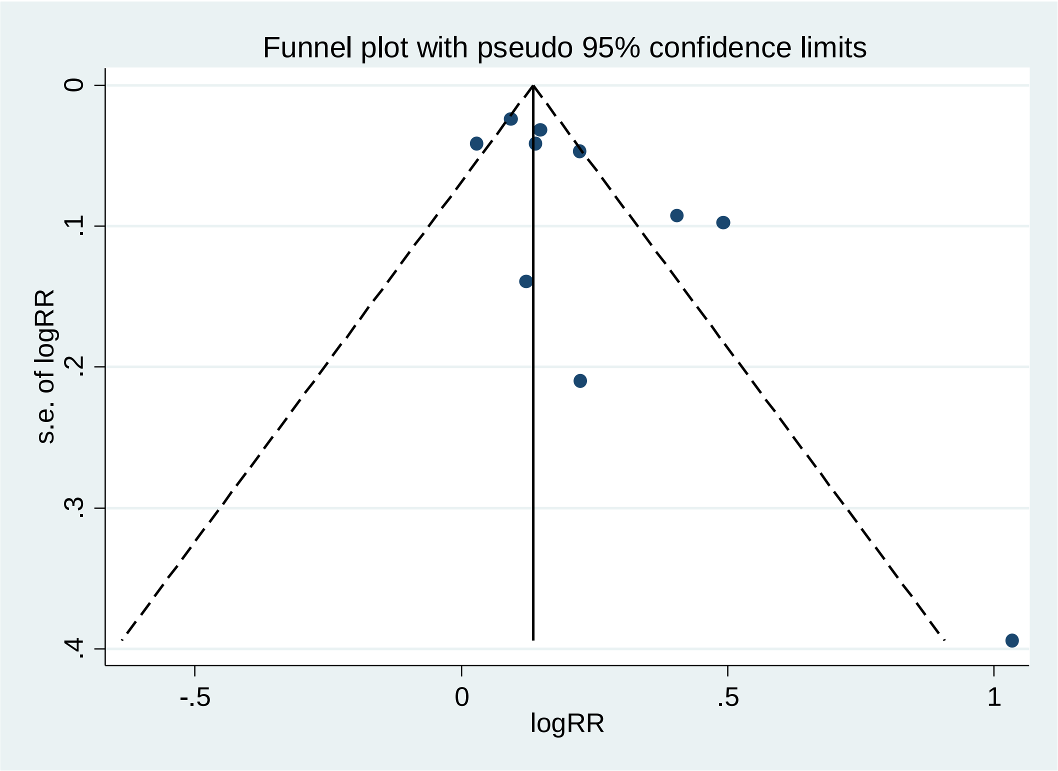

Supplementary Figure S35: Funnel plot showing study precision against the relative risk with 95% CIs for sugar sweetened beverages (dose-response meta-analysis) and risk of T2D. SE = Standard error

Supplementary References S1-88

[1-11]

[1-8, 12-15]

[1, 8, 16-25]

[1, 8, 16-21, 23-26]

[9, 27-32]

[1, 2, 8, 9, 22, 30, 33-36]

[9, 23, 37-46]

[11, 23, 37, 41, 47-61]

[17, 23, 37, 41, 62-70]

[23, 41, 71-79]

[23, 41, 71-78]

[80-88]

1. Meyer KA, Kushi LH, Jacobs DR, Jr., Slavin J, Sellers TA, Folsom AR. Carbohydrates, dietary fiber, and incident type 2 diabetes in older women. Am J Clin Nutr. 2000;71(4):921-30.
2. Ericson U, Sonestedt E, Gullberg B, et al. High intakes of protein and processed meat associate with increased incidence of type 2 diabetes. Br J Nutr. 2013;109(6):1143-53. doi:10.1017/s0007114512003017
3. Kochar J, Djousse L, Gaziano JM. Breakfast cereals and risk of type 2 diabetes in the Physicians' Health Study I. Obesity (Silver Spring, Md.). 2007;15(12):3039-44. doi:10.1038/oby.2007.362
4. Montonen J, Knekt P, Jarvinen R, Aromaa A, Reunanen A. Whole-grain and fiber intake and the incidence of type 2 diabetes. Am J Clin Nutr. 2003;77(3):622-9.
5. Nanri A, Mizoue T, Noda M, et al. Rice intake and type 2 diabetes in Japanese men and women: the Japan Public Health Center-based Prospective Study. Am J Clin Nutr. 2010;92(6):1468-77. doi:10.3945/ajcn.2010.29512
6. Parker ED, Liu S, Van Horn L, et al. The association of whole grain consumption with incident type 2 diabetes: the Women's Health Initiative Observational Study. Annals of epidemiology. 2013;23(6):321-7. doi:10.1016/j.annepidem.2013.03.010
7. Sun Q, Spiegelman D, van Dam RM, et al. White rice, brown rice, and risk of type 2 diabetes in US men and women. Arch Intern Med. 2010;170(11):961-9. doi:10.1001/archinternmed.2010.109
8. Hodge AM, English DR, O'Dea K, Giles GG. Glycemic index and dietary fiber and the risk of type 2 diabetes. Diabetes Care. 2004;27(11):2701-6.
9. von Ruesten A, Feller S, Bergmann MM, Boeing H. Diet and risk of chronic diseases: results from the first 8 years of follow-up in the EPIC-Potsdam study. Eur J Clin Nutr. 2013;67(4):412-9. doi:10.1038/ejcn.2013.7

10. Wirstrom T, Hilding A, Gu HF, Ostenson CG, Bjorklund A. Consumption of whole grain reduces risk of deteriorating glucose tolerance, including progression to prediabetes. Am J Clin Nutr. 2013;97(1):179-87. doi:10.3945/ajcn.112.045583
11. van Dam RM, Hu FB, Rosenberg L, Krishnan S, Palmer JR. Dietary calcium and magnesium, major food sources, and risk of type 2 diabetes in U.S. black women. Diabetes Care. 2006;29(10):2238-43. doi:10.2337/dc06-1014
12. Fung TT, Hu FB, Pereira MA, et al. Whole-grain intake and the risk of type 2 diabetes: a prospective study in men. Am J Clin Nutr. 2002;76(3):535-40.
13. Liu S, Manson JE, Stampfer MJ, et al. A prospective study of whole-grain intake and risk of type 2 diabetes mellitus in US women. Am J Public Health. 2000;90(9):1409-15.
14. Soriguer F, Colomo N, Olveira G, et al. White rice consumption and risk of type 2 diabetes. Clin Nutr. 2013;32(3):481-4. doi:10.1016/j.clnu.2012.11.008
15. Golozar A, Khalili D, Etemadi A, et al. White rice intake and incidence of type-2 diabetes: analysis of two prospective cohort studies from Iran. BMC Public Health. 2017;17(1):133. doi:10.1186/s12889-016-3999-4
16. Qiao Y, Tinker L, Olendzki BC, et al. Racial/ethnic disparities in association between dietary quality and incident diabetes in postmenopausal women in the United States: the Women's Health Initiative 1993-2005. Ethnicity & health. 2014;19(3):328-47. doi:10.1080/13557858.2013.797322
17. Lacoppidan SA, Kyro C, Loft S, et al. Adherence to a Healthy Nordic Food Index Is Associated with a Lower Risk of Type-2 Diabetes--The Danish Diet, Cancer and Health Cohort Study. Nutrients. 2015;7(10):8633-44. doi:10.3390/nu7105418
18. Mamluk L, O'Doherty MG, Orfanos P, et al. Fruit and vegetable intake and risk of incident of type 2 diabetes: results from the consortium on health and ageing network of cohorts in Europe and the United States (CHANCES). Eur J Clin Nutr. 2016. doi:10.1038/ejcn.2016.143
19. Mursu J, Virtanen JK, Tuomainen TP, Nurmi T, Voutilainen S. Intake of fruit, berries, and vegetables and risk of type 2 diabetes in Finnish men: the Kuopio Ischaemic Heart Disease Risk Factor Study. Am J Clin Nutr. 2014;99(2):328-33. doi:10.3945/ajcn.113.069641
20. Cooper AJ, Forouhi NG, Ye Z, et al. Fruit and vegetable intake and type 2 diabetes: EPIC-InterAct prospective study and meta-analysis. Eur J Clin Nutr. 2012;66(10):1082-92. doi:10.1038/ejcn.2012.85
21. Kurotani K, Nanri A, Goto A, et al. Vegetable and fruit intake and risk of type 2 diabetes: Japan Public Health Center-based Prospective Study. Br J Nutr. 2013;109(4):709-17. doi:10.1017/s0007114512001705
22. Bazzano LA, Li TY, Joshipura KJ, Hu FB. Intake of fruit, vegetables, and fruit juices and risk of diabetes in women. Diabetes Care. 2008;31(7):1311-7. doi:10.2337/dc08-0080
23. Montonen J, Jarvinen R, Heliovaara M, Reunanen A, Aromaa A, Knekt P. Food consumption and the incidence of type II diabetes mellitus. Eur J Clin Nutr. 2005;59(3):441-8. doi:10.1038/sj.ejcn.1602094
24. Liu S, Serdula M, Janket SJ, et al. A prospective study of fruit and vegetable intake and the risk of type 2 diabetes in women. Diabetes Care. 2004;27(12):2993-6.
25. Villegas R, Shu XO, Gao YT, et al. Vegetable but not fruit consumption reduces the risk of type 2 diabetes in Chinese women. J Nutr. 2008;138(3):574-80.
26. Muraki I, Imamura F, Manson JE, et al. Fruit consumption and risk of type 2 diabetes: results from three prospective longitudinal cohort studies. Bmj. 2013;347:f5001. doi:10.1136/bmj.f5001
27. Kochar J, Gaziano JM, Djousse L. Nut consumption and risk of type II diabetes in the Physicians' Health Study. Eur J Clin Nutr. 2010;64(1):75-9. doi:10.1038/ejcn.2009.121
28. Parker ED, Harnack LJ, Folsom AR. Nut consumption and risk of type 2 diabetes. Jama. 2003;290(1):38-9; author reply 9-40. doi:10.1001/jama.290.1.38
29. Pan A, Sun Q, Manson JE, Willett WC, Hu FB. Walnut consumption is associated with lower risk of type 2 diabetes in women. J Nutr. 2013;143(4):512-8. doi:10.3945/jn.112.172171
30. Villegas R, Gao YT, Yang G, et al. Legume and soy food intake and the incidence of type 2 diabetes in the Shanghai Women's Health Study. Am J Clin Nutr. 2008;87(1):162-7.

31. Buijsse B, Boeing H, Drogan D, et al. Consumption of fatty foods and incident type 2 diabetes in populations from eight European countries. Eur J Clin Nutr. 2015;69(4):455-61. doi:10.1038/ejcn.2014.249
32. Asghari G, Ghorbani Z, Mirmiran P, Azizi F. Nut consumption is associated with lower incidence of type 2 diabetes: The Tehran Lipid and Glucose Study. Diabetes & Metabolism. 2017;43(1):18-24. doi:http://dx.doi.org/10.1016/j.diabet.2016.09.008
33. Morimoto Y, Steinbrecher A, Kolonel LN, Maskarinec G. Soy consumption is not protective against diabetes in Hawaii: the Multiethnic Cohort. Eur J Clin Nutr. 2011;65(2):279-82. doi:10.1038/ejcn.2010.228
34. Nanri A, Mizoue T, Takahashi Y, et al. Soy product and isoflavone intakes are associated with a lower risk of type 2 diabetes in overweight Japanese women. J Nutr. 2010;140(3):580-6. doi:10.3945/jn.109.116020
35. Mueller NT, Odegaard AO, Gross MD, et al. Soy intake and risk of type 2 diabetes in Chinese Singaporeans [corrected]. Eur J Nutr. 2012;51(8):1033-40. doi:10.1007/s00394-011-0276-2
36. Ding M, Pan A, Manson JE, et al. Consumption of soy foods and isoflavones and risk of type 2 diabetes: a pooled analysis of three US cohorts. Eur J Clin Nutr. 2016. doi:10.1038/ejcn.2016.117
37. Vang A, Singh PN, Lee JW, Haddad EH, Brinegar CH. Meats, processed meats, obesity, weight gain and occurrence of diabetes among adults: findings from Adventist Health Studies. Ann Nutr Metab. 2008;52(2):96-104. doi:10.1159/000121365
38. Djousse L, Gaziano JM, Buring JE, Lee IM. Egg consumption and risk of type 2 diabetes in men and women. Diabetes Care. 2009;32(2):295-300. doi:10.2337/dc08-1271
39. Djousse L, Kamineni A, Nelson TL, et al. Egg consumption and risk of type 2 diabetes in older adults. Am J Clin Nutr. 2010;92(2):422-7. doi:10.3945/ajcn.2010.29406
40. Djousse L, Petrone AB, Hickson DA, et al. Egg consumption and risk of type 2 diabetes among African Americans: The Jackson Heart Study. Clin Nutr. 2016;35(3):679-84. doi:10.1016/j.clnu.2015.04.016
41. Ericson U, Hellstrand S, Brunkwall L, et al. Food sources of fat may clarify the inconsistent role of dietary fat intake for incidence of type 2 diabetes. Am J Clin Nutr. 2015;101(5):1065-80. doi:10.3945/ajcn.114.103010
42. Kurotani K, Nanri A, Goto A, et al. Cholesterol and egg intakes and the risk of type 2 diabetes: the Japan Public Health Center-based Prospective Study. Br J Nutr. 2014;112(10):1636-43. doi:10.1017/s000711451400258x
43. Lajous M, Bijon A, Fagherazzi G, Balkau B, Boutron-Ruault MC, Clavel-Chapelon F. Egg and cholesterol intake and incident type 2 diabetes among French women. Br J Nutr. 2015;114(10):1667-73. doi:10.1017/s0007114515003190
44. Virtanen JK, Mursu J, Tuomainen TP, Virtanen HE, Voutilainen S. Egg consumption and risk of incident type 2 diabetes in men: the Kuopio Ischaemic Heart Disease Risk Factor Study. Am J Clin Nutr. 2015;101(5):1088-96. doi:10.3945/ajcn.114.104109
45. Wallin A, Forouhi NG, Wolk A, Larsson SC. Egg consumption and risk of type 2 diabetes: a prospective study and dose-response meta-analysis. Diabetologia. 2016;59(6):1204-13.
46. Zazpe I, Beunza JJ, Bes-Rastrollo M, Basterra-Gortari FJ, Mari-Sanchis A, Martinez-Gonzalez MA. Egg consumption and risk of type 2 diabetes in a Mediterranean cohort; the sun project. Nutr Hosp. 2013;28(1):105-11. doi:10.3305/nh.2013.28.1.6124
47. Liu S, Choi HK, Ford E, et al. A prospective study of dairy intake and the risk of type 2 diabetes in women. Diabetes Care. 2006;29(7):1579-84. doi:10.2337/dc06-0256
48. Elwood PC, Pickering JE, Fehily AM. Milk and dairy consumption, diabetes and the metabolic syndrome: the Caerphilly prospective study. Journal of epidemiology and community health. 2007;61(8):695-8. doi:10.1136/jech.2006.053157
49. Kirii K, Mizoue T, Iso H, et al. Calcium, vitamin D and dairy intake in relation to type 2 diabetes risk in a Japanese cohort. Diabetologia. 2009;52(12):2542-50. doi:10.1007/s00125-009-1554-x

50. Soedamah-Muthu SS, Masset G, Verberne L, Geleijnse JM, Brunner EJ. Consumption of dairy products and associations with incident diabetes, CHD and mortality in the Whitehall II study. Br J Nutr. 2013;109(4):718-26. doi:10.1017/s0007114512001845
51. Margolis KL, Wei F, de Boer IH, et al. A diet high in low-fat dairy products lowers diabetes risk in postmenopausal women. J Nutr. 2011;141(11):1969-74. doi:10.3945/jn.111.143339
52. Grantham NM, Magliano DJ, Hodge A, Jowett J, Meikle P, Shaw JE. The association between dairy food intake and the incidence of diabetes in Australia: the Australian Diabetes Obesity and Lifestyle Study (AusDiab). Public Health Nutr. 2013;16(2):339-45. doi:10.1017/s1368980012001310
53. Sluijs I, Forouhi NG, Beulens JW, et al. The amount and type of dairy product intake and incident type 2 diabetes: results from the EPIC-InterAct Study. Am J Clin Nutr. 2012;96(2):382-90. doi:10.3945/ajcn.111.021907
54. Struijk EA, Heraclides A, Witte DR, et al. Dairy product intake in relation to glucose regulation indices and risk of type 2 diabetes. Nutr Metab Cardiovasc Dis. 2013;23(9):822-8. doi:10.1016/j.numecd.2012.05.011
55. Louie JC, Flood VM, Rangan AM, et al. Higher regular fat dairy consumption is associated with lower incidence of metabolic syndrome but not type 2 diabetes. Nutr Metab Cardiovasc Dis. 2013;23(9):816-21. doi:10.1016/j.numecd.2012.08.004
56. Zong G, Sun Q, Yu D, et al. Dairy consumption, type 2 diabetes, and changes in cardiometabolic traits: a prospective cohort study of middle-aged and older Chinese in Beijing and Shanghai. Diabetes Care. 2014;37(1):56-63. doi:10.2337/dc13-0975
57. Chen M, Sun Q, Giovannucci E, et al. Dairy consumption and risk of type 2 diabetes: 3 cohorts of US adults and an updated meta-analysis. BMC Med. 2014;12:215. doi:10.1186/s12916-014-0215-1
58. Diaz-Lopez A, Bullo M, Martinez-Gonzalez MA, et al. Dairy product consumption and risk of type 2 diabetes in an elderly Spanish Mediterranean population at high cardiovascular risk. Eur J Nutr. 2016;55(1):349-60. doi:10.1007/s00394-015-0855-8
59. Brouwer-Brolsma EM, van Woudenbergh GJ, Oude Elferink SJ, et al. Intake of different types of dairy and its prospective association with risk of type 2 diabetes: The Rotterdam Study. Nutr Metab Cardiovasc Dis. 2016. doi:10.1016/j.numecd.2016.08.003
60. Villegas R, Gao YT, Dai Q, et al. Dietary calcium and magnesium intakes and the risk of type 2 diabetes: the Shanghai Women's Health Study. Am J Clin Nutr. 2009;89(4):1059-67. doi:10.3945/ajcn.2008.27182
61. Moslehi N, Shab-Bidar S, Mirmiran P, Sadeghi M, Azizi F. Associations between dairy products consumption and risk of type 2 diabetes: Tehran lipid and glucose study. Int J Food Sci Nutr. 2015;66(6):692-9. doi:10.3109/09637486.2015.1034249
62. Patel PS, Sharp SJ, Luben RN, et al. Association between type of dietary fish and seafood intake and the risk of incident type 2 diabetes: the European prospective investigation of cancer (EPIC)-Norfolk cohort study. Diabetes Care. 2009;32(10):1857-63. doi:10.2337/dc09-0116
63. Kaushik M, Mozaffarian D, Spiegelman D, Manson JE, Willett WC, Hu FB. Long-chain omega-3 fatty acids, fish intake, and the risk of type 2 diabetes mellitus. Am J Clin Nutr. 2009;90(3):613-20. doi:10.3945/ajcn.2008.27424
64. van Woudenbergh GJ, van Ballegooijen AJ, Kuijsten A, et al. Eating fish and risk of type 2 diabetes: A population-based, prospective follow-up study. Diabetes Care. 2009;32(11):2021-6. doi:10.2337/dc09-1042
65. Krishnan S, Coogan PF, Boggs DA, Rosenberg L, Palmer JR. Consumption of restaurant foods and incidence of type 2 diabetes in African American women. Am J Clin Nutr. 2010;91(2):465-71. doi:10.3945/ajcn.2009.28682
66. Djousse L, Gaziano JM, Buring JE, Lee IM. Dietary omega-3 fatty acids and fish consumption and risk of type 2 diabetes. Am J Clin Nutr. 2011;93(1):143-50. doi:10.3945/ajcn.110.005603
67. Villegas R, Xiang YB, Elasy T, et al. Fish, shellfish, and long-chain n-3 fatty acid consumption and risk of incident type 2 diabetes in middle-aged Chinese men and women. Am J Clin Nutr. 2011;94(2):543-51. doi:10.3945/ajcn.111.013193

68. Nanri A, Mizoue T, Noda M, et al. Fish intake and type 2 diabetes in Japanese men and women: the Japan Public Health Center-based Prospective Study. Am J Clin Nutr. 2011;94(3):884-91. doi:10.3945/ajcn.111.012252
69. Wallin A, Di Giuseppe D, Orsini N, Akesson A, Forouhi NG, Wolk A. Fish consumption and frying of fish in relation to type 2 diabetes incidence: a prospective cohort study of Swedish men. Eur J Nutr. 2015. doi:10.1007/s00394-015-1132-6
70. Rylander C, Sandanger TM, Engeset D, Lund E. Consumption of lean fish reduces the risk of type 2 diabetes mellitus: a prospective population based cohort study of Norwegian women. PLoS One. 2014;9(2):e89845. doi:10.1371/journal.pone.0089845
71. Mannisto S, Kontto J, Kataja-Tuomola M, Albanes D, Virtamo J. High processed meat consumption is a risk factor of type 2 diabetes in the Alpha-Tocopherol, Beta-Carotene Cancer Prevention study. Br J Nutr. 2010;103(12):1817-22. doi:10.1017/s0007114510000073
72. Song Y, Manson JE, Buring JE, Liu S. A prospective study of red meat consumption and type 2 diabetes in middle-aged and elderly women: the women's health study. Diabetes Care. 2004;27(9):2108-15.
73. Villegas R, Shu XO, Gao YT, et al. The association of meat intake and the risk of type 2 diabetes may be modified by body weight. International journal of medical sciences. 2006;3(4):152-9.
74. Steinbrecher A, Erber E, Grandinetti A, Kolonel LN, Maskarinec G. Meat consumption and risk of type 2 diabetes: the Multiethnic Cohort. Public Health Nutr. 2011;14(4):568-74. doi:10.1017/s1368980010002004
75. Pan A, Sun Q, Bernstein AM, et al. Red meat consumption and risk of type 2 diabetes: 3 cohorts of US adults and an updated meta-analysis. Am J Clin Nutr. 2011;94(4):1088-96. doi:10.3945/ajcn.111.018978
76. Fretts AM, Howard BV, McKnight B, et al. Associations of processed meat and unprocessed red meat intake with incident diabetes: the Strong Heart Family Study. Am J Clin Nutr. 2012;95(3):752-8. doi:10.3945/ajcn.111.029942
77. van Woudenbergh GJ, Kuijsten A, Tigcheler B, et al. Meat consumption and its association with C-reactive protein and incident type 2 diabetes: the Rotterdam Study. Diabetes Care. 2012;35(7):1499-505. doi:10.2337/dc11-1899
78. Mari-Sanchis A, Gea A, Basterra-Gortari FJ, Martinez-Gonzalez MA, Beunza JJ, Bes-Rastrollo M. Meat Consumption and Risk of Developing Type 2 Diabetes in the SUN Project: A Highly Educated Middle-Class Population. PLoS One. 2016;11(7):e0157990. doi:10.1371/journal.pone.0157990
79. Lee DH, Folsom AR, Jacobs DR, Jr. Dietary iron intake and Type 2 diabetes incidence in postmenopausal women: the Iowa Women's Health Study. Diabetologia. 2004;47(2):185-94. doi:10.1007/s00125-003-1307-1
80. Bhupathiraju SN, Pan A, Malik VS, et al. Caffeinated and caffeine-free beverages and risk of type 2 diabetes. Am J Clin Nutr. 2013;97(1):155-66. doi:10.3945/ajcn.112.048603
81. Romaguera D, Norat T, Wark PA, et al. Consumption of sweet beverages and type 2 diabetes incidence in European adults: results from EPIC-InterAct. Diabetologia. 2013;56(7):1520-30. doi:10.1007/s00125-013-2899-8
82. Montonen J, Jarvinen R, Knekt P, Heliovaara M, Reunanen A. Consumption of sweetened beverages and intakes of fructose and glucose predict type 2 diabetes occurrence. J Nutr. 2007;137(6):1447-54.
83. Odegaard AO, Koh WP, Arakawa K, Yu MC, Pereira MA. Soft drink and juice consumption and risk of physician-diagnosed incident type 2 diabetes: the Singapore Chinese Health Study. Am J Epidemiol. 2010;171(6):701-8. doi:10.1093/aje/kwp452
84. Palmer JR, Boggs DA, Krishnan S, Hu FB, Singer M, Rosenberg L. Sugar-sweetened beverages and incidence of type 2 diabetes mellitus in African American women. Arch Intern Med. 2008;168(14):1487-92. doi:10.1001/archinte.168.14.1487
85. Paynter NP, Yeh HC, Voutilainen S, et al. Coffee and sweetened beverage consumption and the risk of type 2 diabetes mellitus: the atherosclerosis risk in communities study. Am J Epidemiol. 2006;164(11):1075-84. doi:10.1093/aje/kwj323

86. Sakurai M, Nakamura K, Miura K, et al. Sugar-sweetened beverage and diet soda consumption and the 7-year risk for type 2 diabetes mellitus in middle-aged Japanese men. Eur J Nutr. 2014;53(1):251-8. doi:10.1007/s00394-013-0523-9
87. Schulze MB, Manson JE, Ludwig DS, et al. Sugar-sweetened beverages, weight gain, and incidence of type 2 diabetes in young and middle-aged women. Jama. 2004;292(8):927-34. doi:10.1001/jama.292.8.927
88. Eshak ES, Iso H, Mizoue T, Inoue M, Noda M, Tsugane S. Soft drink, 100% fruit juice, and vegetable juice intakes and risk of diabetes mellitus. Clin Nutr. 2013;32(2):300-8. doi:10.1016/j.clnu.2012.08.003
